# Cortical organoids model early brain development disrupted by 16p11.2 copy number variants in autism

**DOI:** 10.1038/s41380-021-01243-6

**Published:** 2021-08-26

**Authors:** Jorge Urresti, Pan Zhang, Patricia Moran-Losada, Nam-Kyung Yu, Priscilla D. Negraes, Cleber A. Trujillo, Danny Antaki, Megha Amar, Kevin Chau, Akula Bala Pramod, Jolene Diedrich, Leon Tejwani, Sarah Romero, Jonathan Sebat, John R. Yates III, Alysson R. Muotri, Lilia M. Iakoucheva

**Affiliations:** 1 Department of Psychiatry, University of California San Diego, La Jolla, CA USA; 2 Department of Molecular Medicine, The Scripps Research Institute, La Jolla, CA USA; 3 Department of Cellular & Molecular Medicine, University of California San Diego, La Jolla, CA USA; 4 Department of Pediatrics/Rady Children’s Hospital San Diego, University of California, San Diego, La Jolla, CA USA; 5 University of California San Diego, Beyster Center for Psychiatric Genomics, La Jolla, CA USA; 6 University of California San Diego, Kavli Institute for Brain and Mind, La Jolla, CA USA; 7 Center for Academic Research and Training in Anthropogeny (CARTA), La Jolla, CA USA

**Keywords:** Stem cells, Neuroscience, Autism spectrum disorders, Biological techniques, Genetics

## Abstract

Reciprocal deletion and duplication of the 16p11.2 region is the most common copy number variation (CNV) associated with autism spectrum disorders. We generated cortical organoids from skin fibroblasts of patients with 16p11.2 CNV to investigate impacted neurodevelopmental processes. We show that organoid size recapitulates macrocephaly and microcephaly phenotypes observed in the patients with 16p11.2 deletions and duplications. The CNV dosage affects neuronal maturation, proliferation, and synapse number, in addition to its effect on organoid size. We demonstrate that 16p11.2 CNV alters the ratio of neurons to neural progenitors in organoids during early neurogenesis, with a significant excess of neurons and depletion of neural progenitors observed in deletions. Transcriptomic and proteomic profiling revealed multiple pathways dysregulated by the 16p11.2 CNV, including neuron migration, actin cytoskeleton, ion channel activity, synaptic-related functions, and Wnt signaling. The level of the active form of small GTPase RhoA was increased in both, deletions and duplications. Inhibition of RhoA activity rescued migration deficits, but not neurite outgrowth. This study provides insights into potential neurobiological mechanisms behind the 16p11.2 CNV during neocortical development.

## Introduction

Over the last decade, it has been convincingly demonstrated that deletions (DEL) and duplications (DUP) of large genomic regions, or copy number variants (CNVs), are associated with multiple neurodevelopmental disorders [1–4]. The DEL of a genomic region spanning 29 genes on human chromosome 16, 16p11.2 CNV, had been identified as one of the strongest risk factors for autism spectrum disorder (ASD) and intellectual disability (ID), whereas the DUP of the same region were associated with ASD, ID, schizophrenia (SCZ) and bipolar disorder (BD) [2, 3, 5–7]. Most importantly, DEL and DUP were associated with macrocephaly and microcephaly in human carriers, respectively [8, 9]. This phenotype, however, had not been fully recapitulated in mouse models at the whole-brain volume level, although some of the animal studies have reported a mirror effect of 16p11.2 CNV on regional brain volumes [10–12]. There was also little direct concordance in the brain cytoarchitecture, behavior and viability phenotypes between human and mouse models, and at least one of the mouse models observed phenotypes opposite to humans: DEL 16p11.2 mice were smaller and lean, whereas DUP 16p11.2 mice were larger and obese [12].

Significant progress has been made for implicating various biological mechanisms that may be impacted by the 16p11.2 CNV. RNA sequencing of cortex from 16p11.2 deletion and duplication mice identified altered expression of genes and networks that converged on general ASD-associated pathways including synaptic function, chromatin modification and transcriptional regulation [13]. Dysregulation of ciliopathy genes [14], ERK/MAPK signaling [15, 16], and metabotropic glutamate receptor 5 (mGluR5)-dependent synaptic plasticity and protein synthesis [17] in mouse models were also implicated. Transcriptome profiling of lymphoblastoid cell lines of 16p11.2 CNV human carriers identified expression dysregulation of the neuronal-related gene in deletion, but not in duplication [18]. Despite the progress made with regard to the understanding of the general mechanisms disrupted by the 16p11.2 CNV in animal models and non-neuronal human cells, the question of how 16p11.2 variants impact early human brain development remained unanswered.

Recent advances in stem cell technologies opened a window of opportunities for investigating brain disorders using human-based in vitro systems [19]. Patient-derived or CRISPR/Cas9 genome-edited induced pluripotent stem cells (iPSCs) reprogrammed into two-dimensional (2D) monolayer cultures are beginning to provide new insights into neurodevelopmental disorders [20, 21]. Such 2D models were recently used to investigate cellular phenotypes of the 16p11.2 CNV, and observed reduced synaptic density in both genotypes, as well as an impact of this CNV on neuronal size and dendrite length [22]. However, the 2D models are known to have certain limitations, such as the loss of the complex 3D heterotypic environment in which the cells normally reside in vivo, as well as limited cell-cell communications and cell–matrix mechanics. These shortcomings of 2D models are beginning to be addressed with 3D organoid models [23] that have proven advantages over 2D models for investigating human brain diseases [24–26]. Characterization of these models demonstrated that they closely resemble the human fetal brain, forming structures reminiscent of deeper cortical layers and sharing cell types and transcriptomic signatures with the fetal brain [27–30]. These models are particularly well-suited for investigating early-onset diseases because their maturity recapitulates fetal and early postnatal brain development [24–26, 30], despite noted certain limitations [31]. Many studies had used 3D cortical organoids to model lissencephaly [32, 33], non-syndromic autism [34], autosomal recessive primary microcephaly [23], and Timothy syndrome [35]. Here, we used patient-derived cortical organoids to perform 3D modeling of fetal brain development of the most common autism subtype associated with DEL and DUP of the 16p11.2 CNV.

**Fig. 1:**
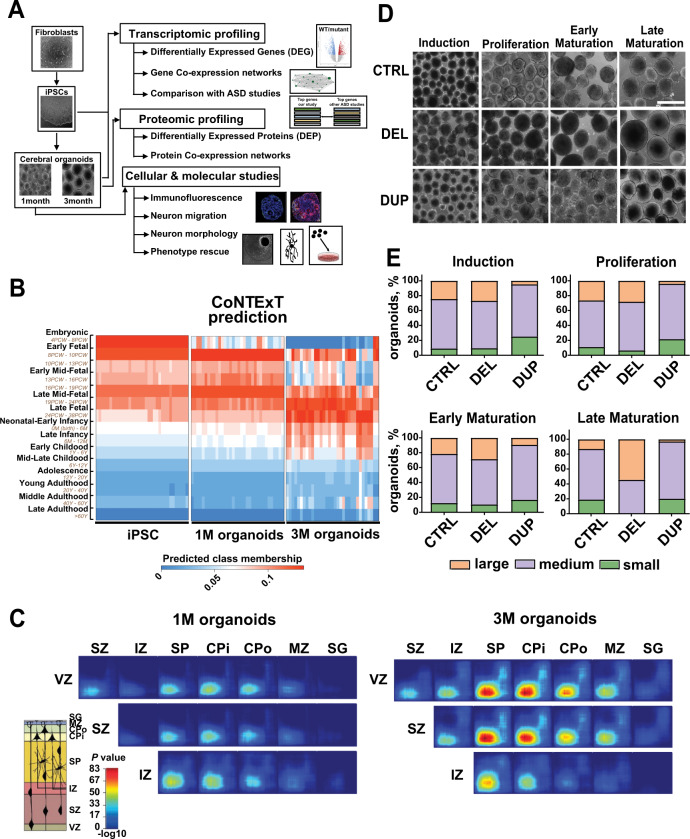
Generation and characterization of cortical organoids from 16p11.2 patient and control iPSCs. **A** Overview of the study design and analyses. **B** Comparison of iPSCs and organoids transcriptomes with the human developing brain transcriptome using CoNTExT [40]. The samples from three individuals of each genotype (CTRL, DEL, DUP), with two clones per individual and two replicates per clone (*n* = 36 datasets) are shown for each group (iPSC, 1 M and 3 M organoids). PCW post conception weeks, M months, Y years. **C** Predicted laminar transitions for 1 M and 3 M organoids using TMAP [40] and the transcriptome of laser capture microdissected cortical laminae from postmortem human fetal brain (15–21 PCW) [41]. Rank–rank hypergeometric overlap (RRHO) maps for CTRL organoids (*n* = 12 datasets) from 3 patients, 2 clones per patient, 2 replicates per clone are shown, with CTRL iPSCs (*n* = 12 datasets) used as a second time point. Each pixel represents the overlap between fetal brain and organoids transcriptome, color-coded according to the −log_10_ *p*-value of a hypergeometric test. On each map, the extent of shared upregulated genes is displayed in the bottom left corner, whereas shared downregulated genes are displayed in the top right corners. **D** Representative images of cortical organoids for each genotype (CTRL, DEL, DUP) at different time points of differentiation: induction (6 days of differentiation), proliferation (16 days of differentiation), early maturation (1 M of differentiation), and late maturation (3 M of differentiation). Scale bar: 1000 µm. **E** The analysis of size differences between cortical organoids of each genotype (CTRL, DEL, and DUP) at different time points of differentiation. Organoids’ (*n* > 100 for each genotype) diameter was measured using ImageJ, size distribution was built to define large and small organoids as mean ± SEM within each separate experiment, and the medium group was comprised of the remaining organoids. The average of all experiments is shown, individual bin comparison for each group (small, medium, and large) together with statistical analyses using one-way ANOVA with Tukey’s multiple comparisons are shown in Fig. S7 and Table S3.

In this study, we generated iPSCs and cortical organoids from the 16p11.2 DEL and DUP patient fibroblasts and unrelated healthy control (CTRL) individuals and investigated molecular and cellular processes that were disrupted by this genetic variant (Fig. 1A). We found that the size of deletion organoids is larger, and duplication organoids are smaller, recapitulating the mirror effect of 16p11.2 CNV on brain size in humans. Transcriptomic and proteomic profiling of organoids identified genes, proteins, and co-expression modules impacted by the 16p11.2 CNV. The results were validated by a panel of orthogonal assays. Cellular assays confirmed that 16p11.2 CNV impacts neuronal maturation, migration, morphology, and synaptic processes, implicating defects in neurogenesis. We identified multiple pathways disrupted by the 16p11.2 CNV, including cell locomotion and motility, ion channel activity, actin cytoskeleton, synaptic-related processes, along with Wnt and RhoA signaling. The activation of RhoA signaling was a likely contributor to defects in neuronal migration in both DELs and DUPs because of the inhibition of RhoA activity with Rhosin rescued migration deficits in both genotypes. Our study makes a significant contribution to the understanding of neurobiological mechanisms that may be disrupted during early human neocortical development in the 16p11.2 CNV carriers, and offers a potential path for therapeutic interventions.

## Results

### Cortical organoids maturation resembles stages of human brain development

To investigate how 16p11.2 CNV impacts early stages of human brain development, and what molecular pathways are dysregulated by this genetic variant, we generated cortical organoids from the 16p11.2 CNV carriers. We first obtained iPSCs by reprogramming patient- and control-derived fibroblasts using episomal transduction, and then differentiated iPSCs into cortical organoids as previously described [36].

We selected six male 16p11.2 CNV carriers with extreme head size phenotypes (age-normalized head circumference *Z*-score range from 2.51 to 4.32 in DELs; and from −0.69 to −1.63 in DUPs) for this study. We decided to focus on this phenotype, because previous studies from our and other laboratories using infection with Zika virus were able to successfully recapitulate microcephaly in organoid models [37, 38]. The restriction to only male gender was due to samples availability. The details of patients’ phenotypes are described in Table S1. Three gender-matched healthy unrelated individuals that did not carry 16p11.2 CNV were used as controls. We performed rigorous quality control assessment of reprogrammed iPSCs clones using immunofluorescence (Fig. S1) and real-time quantitative polymerase chain reaction (RT-qPCR) (Fig. S2) for pluripotency markers, by comparing them to parental fibroblasts using single-nucleotide polymorphism array genotyping (Fig. S3), and by RT-qPCR for cell type-specific markers across different developmental stages starting from iPSCs and ending with organoids (Fig. S4). After confirming the presence of 16p11.2 CNV in patient samples and ensuring that no additional CNVs were introduced by reprogramming, we selected two clones per individual for organoids production. We performed bulk RNA sequencing (RNA-seq) of a total of 108 samples derived from iPSCs, 1-month-old (1 M) and 3-month-old (3 M) organoids (36 samples at each time point). We sequenced two clones per individual, and two replicates per clone from for all three genotypes (3 DELs, 3 DUPs, and 3 CTRLs) (Fig. S5). RNA sequencing quality control parameters are shown in Table S2.

To investigate whether developmental maturity and laminar organization of produced organoids resembled the human brain, we compared transcriptional profiles of iPSCs and organoids with the atlas of the developing human brain [39] using CoNTExT [40]. Transcriptional profiles of iPSCs from all individuals closely matched those of embryonic (4–8 PCW) and early fetal (8–10 PCW) human brain, independently validating successful conversion of fibroblasts into a pluripotent state by reprogramming (Fig. 1B). Transcriptional profiles of 1-month-old organoids resembled those of early mid-fetal (13–16 PCW) through late mid-fetal (19–24 PCW) periods. Likewise, transcriptional profiles of 3-month-old organoids mostly recapitulated those of late mid-fetal (19–24 PCW) through neonatal-early infancy (birth to 6 months) developmental periods.

Next, we examined the degree of overlap between in vivo cortical development of prenatal human brain and our in vitro differentiated organoids using TMAP [40]. We compared transcriptional profiles of our organoids with those derived from laser capture microdissected cortical laminae of postmortem human fetal brain (15–21 PCW) [41]. TMAP performs serialized differential expression analysis between any two in vivo developmental periods and any two in vitro differentiation time points, followed by quantification of overlap [40]. Laminar matching by TMAP demonstrated transitions between proliferative layers (ventricular VZ, subventricular SZ, and intermediate IZ zones) and post mitotic upper layers for both, 1 M and 3 M old organoids (Fig. 1C). We observed that laminar transition into upper layers manifested a greater shift in 3 M organoids than in 1 M organoids. For example, greater correspondence to upper layers (subplate SP, cortical plate inner CPi and outer CPo layers and marginal zone MZ) was visible at 3 M compared to 1 M, consistent with the increased maturity at 3 M. We replicated this maturation shift using an additional independent dataset from the fetal human brain [42] (Fig. S6). Together, the results suggest that cortical organoids from DEL, DUP, and CTRL individuals mature over time, closely recapitulating human brain development in terms of temporal transitions and laminar organization. Furthermore, organoids between 1 M and 3 M of differentiation most closely resemble human mid-fetal brain development and represent suitable models for studying the molecular basis of neurodevelopmental disorders, considering a proven role of this period in ASD and SCZ pathogenesis [43–45]. These results are in agreement with a previous study that concluded that brain organoids faithfully recapitulate fetal development at the transcriptional level [46].

### Patient-derived organoids recapitulate macrocephaly and microcephaly phenotypes

Since our patients with 16p11.2 DELs and DUPs were selected based on the extreme head circumference phenotypes (Table S1), we investigated whether organoids recapitulate these phenotypes. We measured the diameter of 16p11.2 and control organoids at four time points, at day 6 (D6, neural induction), day 16 (D16, proliferation), 1 month (1 M, early maturation), and 3 months (3 M, late maturation). We observed a higher proportion of large organoids in DELs and a higher proportion of small organoids in DUPs for DELs vs. DUPs comparison at almost all time points (Fig. 1D, E and Fig. S7), with the differences reaching statistical significance at the late maturation stage for DELs vs. CTRL and at the induction and proliferation stages for DUPs vs. CTRL **(**Table S3). By three months, DELs were completely devoid of small organoids, and the proportion of large organoids in DUPs was very low. These results demonstrate that cortical organoids recapitulate patients’ brain size phenotypes.

### Differential gene expression analysis points to dysregulation of multiple pathways by 16p11.2 CNV

To understand molecular pathways dysregulated by the 16p11.2 CNV, we performed differential gene expression analyses of 108 transcriptomes derived from iPSCs, 1 and 3 M organoids (Materials and methods). Extensive quality control and normalization included sample outlier detection, principal component analyses, surrogate variable analysis, and covariates selection with MARS (Materials and methods and Fig. S8 and Fig. S9). For gene differential expression analyses, we implemented the limma-voom model with “duplicateCorrelation” function to account for duplicate samples (clones and replicas) from the same individuals, and to avoid pseudo-replication in the analyses [47].

**Fig. 2:**
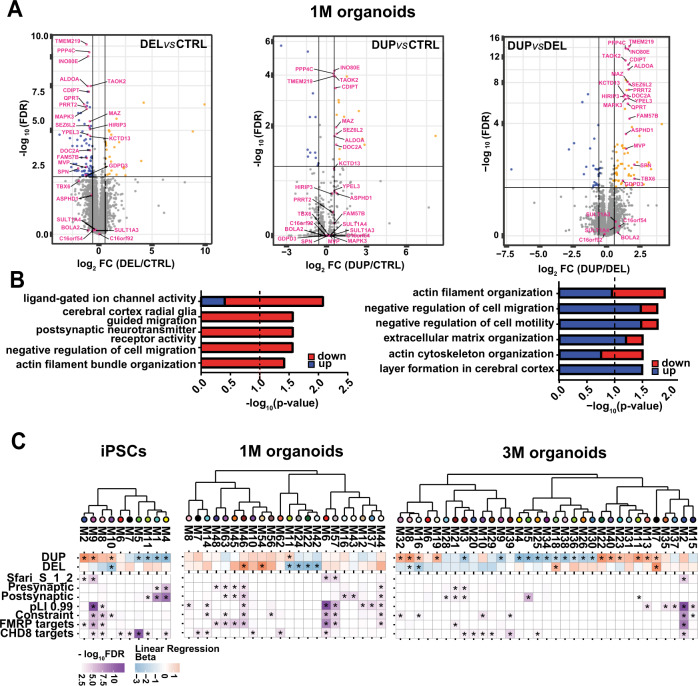
Differential gene expression and gene co-expression analyses in iPSCs and cortical organoids. **A** Volcano plots of differentially expressed genes for DEL vs. CTRL (left), DUP vs. CTRL (middle), and DUP vs. DEL (right) in 1 M organoids. Genes within 16p11.2 CNV *locus* are colored in pink. Genes colored in orange are upregulated; genes colored in blue are downregulated. **B** Gene Ontology enrichment analyses are shown as bar plots for DEL vs. CTRL (left) and DUP vs. DEL (right) comparisons. DUP vs. CTRL comparison did not identify significant GO terms. The contribution of upregulated or downregulated genes to specific GO terms are shown in blue and red, respectively. **C** Hierarchical clustering of gene co-expression modules by module eigengene. Modules that are statistically significantly associated with DEL and DUP genotypes at FDR < 0.1 are marked with asterisk (*). Module enrichment analyses against literature-curated gene lists with previous evidence for involvement in autism are shown at the bottom, asterisks (*) correspond to FDR < 0.05. The lists include syndromic and highly ranked (1 and 2) genes from SFARI Gene database (https://gene.sfari.org/database/gene-scoring/); pre- and post-synaptic genes from SynaptomeDB [110]; genes with probability of loss-of-function intolerance (pLI) > 0.99 as reported by the exome aggregation consortium [111]; constrained genes [112]; FMRP target genes [113], and CHD8 target genes [114]. Only the modules with at least one significant enrichments (i.e., at least one asterisk (*)) across all described analyses are shown.

We identified 185, 255, and 1044 differentially expressed genes (DEGs) in DELs vs. CTRLs, DUPs vs. CTRLs, and DUPs vs. DELs in iPSCs, respectively (Fig. S10); 132, 35, and 118 DEGs in DELs vs. CTRLs, DUPs vs. CTRLs, and DUPs vs. DELs in 1 M organoids, respectively (Fig. 2A); 52, 345, and 430 DEGs in DELs vs. CTRLs, DUPs vs. CTRLs, and DUPs vs. DELs in 3 M organoids, respectively (Fig. S10) at 10% false-discovery rate (FDR) (Table S4). The majority of the genes from the 16p11.2 *locus* were most significantly dysregulated in all datasets, confirming the expected *cis*-effect of CNV on gene expression. In addition, 16p11.2 CNV had a significant effect on the expression of many genes outside of the *locus*. Gene Ontology (GO) analyses of DEGs in DELs vs. CTRLs in 1 M organoids revealed significant enrichment in “ligand-gated ion channel activity”, “cerebral cortex radial glia (RG)-guided migration”, “postsynaptic neurotransmitter receptor activity”, and multiple other migrations and motility-related processes, such as “negative regulation of cell migration”, “negative regulation of cell motility”, “negative regulation of locomotion”, and “cerebral cortex cell migration” (Fig. 2B and Table S5). The GO analyses of DUPs vs. CTRLs did not reveal any enriched GO functions, likely due to a limited number of DEGs (e.g., 35 DEGs); whereas DUPs vs. DELs comparison identified DEGs with many functions related to the actin cytoskeleton, “extracellular matrix organization”, and “layer formation in the cerebral cortex” (Fig. 2B and Table S5). In agreement with DELs vs. CTRLs results, DUPs vs. DELs also identified “cerebral cortex RG-guided migration”, “regulation of cell migration”, and “neuron migration” functions (Table S5).

In iPSCs, the majority of GO functions for DELs vs. CTRLs and DUPs vs. CTRLs were related to potassium and sodium ion transport and homeostasis, whereas for DUPs vs. DELs, in addition to these functions, “cell motility”, “regulation of locomotion”, and “cell migration” were also observed (Fig. S10 and Table S5). In 3 M organoids, DELs vs. CTRLs comparison revealed GO functions related to ion channel regulation and transport, as well as synaptic and *trans*-synaptic signaling; the DUPs vs. CTRLs DEGs were enriched in “translation”, “intracellular protein transport”, and “long-term synaptic depression” GO functions among others (Fig. S10 and Table S5), whereas DUPs vs. DELs DEGs were enriched in “nervous system development”, synaptic and neuron differentiation-related functions, as well as “generation of neurons” and “regulation of neuron migration” among many others (Fig. S10 and Table S5). Although many different GO functions were identified, with some functions unique to individual datasets (such as “translation” for DUPs vs. CTRLs in 3 M organoids), there were also some overlapping functions (such as those related to cell migration, motility, and locomotion) among the datasets.

### Gene co-expression analyses identifies migration and synaptic/neuronal modules

To further characterize signaling pathways and molecular processes dysregulated by the 16p11.2 CNV, we performed weighted gene co-expression network analysis (WGCNA) [48] that identified modules of genes with correlated expression in DEL, DUP, and CTRL samples (Materials and methods and Fig. S11). Overall, we identified 11, 63, and 41 modules in iPSC, 1 M and 3 M organoids, respectively (Table S6). When these modules were statistically tested for association with DEL and DUP genotypes, thirty-five modules (7 in iPSC, 6 in 1 M, and 21 in 3 M organoids) were detected as significantly positively or negatively associated with genotypes at 10% FDR (Fig. 2C and Fig. S12). We detected a single module in each dataset that contained 16p11.2 CNV genes (*10purple* in iPSCs, *11greenyellow* in 1 M, and *16lightcyan* in 3 M organoids), and these modules were positively associated with DUPs and negatively associated with DELs at each of the time points (Fig. S13). GO annotation of genetype-associated modules revealed interesting biological functions that covered a wide range of processes (Table S7). Notable GO functions included cell migration and motility (*22darkgreen* in 1 M and *32violet* in 3 M organoids), synaptic signaling and neuron differentiation (*46brown4* in 1 M and *25orange* in 3 M organoids), chromatin organization (*11greenyellow* in iPSCs), cilium assembly (*19lightyellow* in 3 M organoids), and mitochondrial respiration (*3brown* in 3 M organoids) (Table S7). Whereas some module GO functional annotations were unique, modules with migration and synaptic/neuronal functions were shared between 1 M and 3 M organoids. Interestingly, the modules with these GO functions were associated with genotypes in the opposite directions—migration module had negative association with DELs (1 M *22darkgreen*) and positive association with DUPs (3 M *32violet*), while synaptic/neuronal module had positive association with DELs (1 M *46brown4*) and negative with DUPs (3 M *25orange*) (Fig. 2C and Fig. S12). This suggests that 16p11.2 CNV differently impacts migration and neurogenesis functions in organoids.

To further investigate how co-expression modules and their function contribute to existing knowledge of ASD genetics, we performed statistical enrichment analyses of co-expression modules against curated gene lists with previous evidence for the involvement in autism (Materials and methods). We observed one module in each dataset with similar enrichment signatures (M9- *9magenta* in iPSCs, M6—*6red* in 1 M and M2—*2blue* in 3 M organoids) (Fig. 2C). These modules were enriched in highly confident ASD risk genes, constrained and highly intolerant to mutations (pLI > 0.99) genes, as well as CHD8 and FMRP target genes in all datasets. GO analyses of these modules revealed shared biological functions related to histone modification and chromatin organization, with many ASD risk genes found within these modules. Chromatin-modifying and remodeling genes (CHD8, ARID1B, ASH1L, KMT2A, and SETD5) are known to be frequently mutated in ASD patients, suggesting that 16p11.2 CNV impacts gene regulatory networks that overlap with other ASD (and NDD) genes. We also observed several modules enriched in presynaptic or postsynaptic genes. In summary, both DEG and WGCNA analyses in organoids, suggest that the processes dysregulated by the 16p11.2 CNV at the transcriptome level converge on migration, synaptic/neuronal, and chromatin-related functions.

### The 16p11.2 CNV impacts organoids proteome

In addition to impacting the organoid’s transcriptome, the deletion and duplication of 29 genes within 16p11.2 CNV could have profound impact at the post-transcriptional level. To fully characterize the impact of the 16p11.2 CNV and to detect underlying molecular mechanisms, we performed proteomic profiling of organoids with tandem mass tag mass spectrometry (TMT-MS), from the same samples as those used for RNA-seq experiments (Fig. S14). We detected a total of 6126 proteins in 1 M and 5481 proteins in 3 M organoids, with 13 and 11 proteins from within 16p11.2 CNV, respectively.

**Fig. 3:**
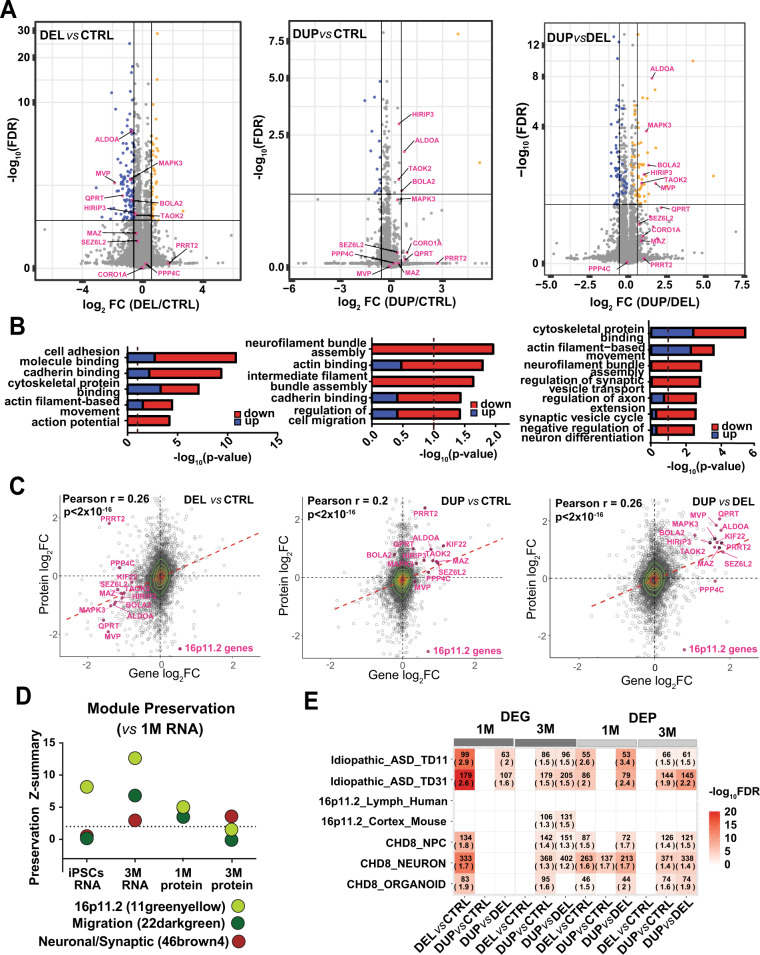
Proteomic analyses of cortical organoids and correlation between transcriptomes and proteomes. **A** Volcano plots of differentially expressed proteins for DEL vs. CTRL (left), DUP vs. CTRL (middle), and DUP vs. DEL (right) in 1 M organoids. Proteins within 16p11.2 CNV *locus* are colored in pink. Proteins colored in orange are upregulated; proteins colored in blue are downregulated. **B** Gene Ontology enrichment analyses are shown as bar plots for DEL vs. CTRL (left), DUP vs. CTRL (middle), and DUP vs. DEL (right) comparisons. The contribution of upregulated or downregulated proteins to specific GO terms are shown in blue and red, respectively. **C** Correlation of entire transcriptomes *vs* proteomes in DEL vs. CTRL (left), DUP vs. CTRL (middle), and DUP vs. DEL (right) comparisons for 1 M organoids. Genes/proteins within 16p11.2 CNV *locus* are colored in pink. **D** Module preservation analyses for 16p11.2, migration, and neuronal/synaptic modules detected in transcriptomic profiling of 1 M organoids as compared to other datasets (iPSC and 3 M transcriptomic, and 1 M and 3 M proteomic datasets). The neuronal/synaptic module vs. 1 M protein is located behind the 16p11.2 module and is not visible. **E** Comparison of differentially expressed genes and proteins from this study with differentially expressed genes from other relevant studies. Idiopathic_ASD_TD11 and Idiopathic_ASD_TD31 are differentially expressed genes from organoids derived from individuals with idiopathic ASD at 11th and 31st day of differentiation from the Mariani study [34]; 16p11.2_Lymph_Human and 16p11.2_Cortex_Mouse have differentially expressed genes from human lymphoblastoid cell lines of ASD patients with 16p11.2 CNV and 16p11.2 deletion mouse cortex, respectively, from the Blumenthal study [13]; CHD8_NPC, CHD8_NEURON, and CHD8_ORGANOID are differentially expressed genes from isogenic CRISPR/Cas9 generated heterozygous CHD8 neural progenitors, monolayer neurons and organoids from the Wang study [50]. Number of overlapped genes and odds ratio (in parenthesis) are indicated inside each cell, and provided only for FDR ≤ 0.05 and OR > 1.

We identified 517, 100, and 305 differentially expressed proteins (DEPs) in DELs vs. CTRLs, DUPs vs. CTRLs, and DUPs vs. DELs in 1 M organoids, respectively (Fig. 3A); 64, 1108, and 970 DEPs in DELs vs. CTRLs, DUPs vs. CTRLs, and DUPs vs. DELs in 3 M organoids, respectively (Fig. S15) at 10% FDR (Table S8). In proteomic data, the *cis*-effect of 16p11.2 CNV was weaker than in RNA-seq, possibly due to the lower dynamic range between RNA and protein detectability in transcriptomic *vs* proteomic experiments [49]. Specifically, out of 29 16p11.2 proteins, only 13 were detectable in our proteomics experiments, pointing to lower coverage by proteomics. However, the number of detected DEPs were *on par* or even greater than DEGs. Furthermore, patterns of proteome-wide effect of the 16p11.2 CNV on proteins outside of the *locus* were similar to the transcriptome-wide effect, with greater number of DEPs observed in 3 M organoids compared to 1 M organoids.

GO annotations of DEPs in 1 M organoids identified actin cytoskeleton-related functions shared across all genotype comparisons (Fig. 3B and Table S9), with additional GO functions such as “action potential” in DELs vs. CTRLs; “regulation of cell migration”, “regulation of locomotion”, “regulation of cell motility” in DUPs vs. CTRLs; and “signal release from synapse”, “neurotransmitter secretion”, “negative regulation of neurogenesis”, and “regulation of synaptic vesicle exocytosis” in DUPs vs. DELs (Table S9). In 3 M organoids, cytoskeletal-related GO processes were enriched in DELs vs. CTRLs; axon and neuron development and differentiation functions were enriched in DUPs vs. CTRLs; and cytoskeletal, neuronal, and locomotion functions were enriched in DUPs vs. DELs (Fig. S15). The DEPs shared by 1 M and 3 M organoids included synaptic (SYN1, STX1B, and SYNJ1), cytoskeletal (MAPT, TUBB4A, and TRIO) and cell adhesion (NCAM and CNTN1) proteins that were downregulated in DUPs and upregulated in DELs. Similar trends were observed for several high-confident autism-associated proteins (ANK2, DPYSL2, STXBP1, and DYNC1H1). This suggests that 16p11.2 CNV impacts proteins outside of the *locus*, with particular effect on cytoskeletal, synaptic, and autism-relevant proteins.

To further investigate how protein co-expression modules are impacted by the 16p11.2 CNV in DEL and DUP patient-derived cortical organoids, we performed weighted protein co-expression network analysis (WPCNA) using TMT-MS proteomic data (Materials and methods and Fig. S16). We identified 21 and 17 protein co-expression modules in 1 M and 3 M organoids, respectively (Table S10). Twelve modules (5 in 1 M and 7 in 3 M organoids) were significantly associated with DEL or DUP genotypes at 10% FDR (Fig. S17). The significant modules included those enriched in RNA splicing and chromatin organization (*5green* in 1 M organoids), ribosome and translation (*2blue* in 3 M organoids), cytoskeleton and microtubule (*7black* in 3 M organoids), and mitochondrial respiration (*8pink* in 3 M organoids) GO functions (Table S11). One module detected in 3 M organoids by WPCNA, *1turquoise* (M1), was enriched in pre- and postsynaptic, constrained and FMRP target proteins (Fig. S18). It included proteins involved in processes related to neuron differentiation and neurogenesis, neuron projection development, synaptic signaling, cytoskeleton organization, actin filament processes, as well as migration and locomotion (Table S11). Many of these functions were also identified by RNA-seq profiling, pointing to the convergence of molecular processes at the transcriptome and proteome levels.

### Biological convergence of organoids transcriptome and proteome

To determine the extent of convergence between organoids transcriptomes and proteomes, we calculated correlation coefficient of expression levels for genes and proteins (Fig. 3C). Globally, we observed positive correlation between transcriptomes and proteomes in 1 M organoids (DELs vs. CTRLs Pearson *r* = 0.26; DUPs vs. CTRLs *r* = 0.2; DUPs vs. DELs *r* = 0.26; *p* < 2 × 10^−16^) (Fig. 3C) and in 3 M organoids (DELs vs. CTRLs Pearson *r* = 0.17; DUPs vs. CTRLs *r* = 0.19; DUPs vs. DELs *r* = 0.1; *p* < 2 × 10^−16^) (Fig. S19). The correlation increased further (Pearson *r* = 0.79, *p* < 2 × 10^−16^) when we combined DEGs vs. proteome and DEPs vs. transcriptome, either with or without 16p11.2 genes/proteins (Fig. S20). We then carried out module preservation analyses to identify conserved modules across these two levels of regulation. This analysis demonstrated a high degree of preservation (*Z*-summary > 2) at the RNA and protein level for most modules that were significantly associated with genotype in 1 M organoids (Fig. S21), and slightly lower degree of preservation in 3 M organoids (Fig. S21). The 16p11.2, migration and neuronal/synaptic modes had a high degree of preservation in both, 1 M organoids (Fig. 3D) and 3 M organoids (Fig. S21). The module containing 16p11.2 genes had the highest preservation at the transcriptional level (Fig. 3D). Overall, we observed that organoids transcriptomes and proteomes have a positive correlation, especially when normalized by the coverage (Fig. S20); gene and protein co-expression modules associated with DEL and DUP genotypes demonstrate a high degree of preservation, especially in 1 M organoids; the DEGs and DEPs, as well as transcriptomic and proteomic modules, share GO functional annotations related to actin cytoskeletal processes, migration, and motility, neuronal/ synaptic and other functions.

To put our results into the context of previous studies, we performed enrichment analyses our DEGs and DEPs against other datasets with relevance to ASD. Specifically, our DEGs and DEPs were compared with transcriptomes of 16p11.2 patients’ lymphoblast lines and cerebral cortex of 16p11.2 mice [13], idiopathic ASD patient-derived organoids [34], and CHD8 KO organoids, NPCs, and neurons [50] (Fig. 3E). Overall, we observed a greater overlap of our DEGs and DEPs with DEGs identified in idiopathic ASD organoid models, suggesting that 16p11.2 organoids share transcriptomic signatures with other ASD subtypes. There were no overlap of our datasets with the transcriptomes from 16p11.2 patients’ lymphoblastoid cell lines, and a very limited overlap (only for 3 M DEGs) with the 16p11.2 mouse cortex. We observed a good overlap of our data with CHD8 neurons, NPCs, and organoids. In summary, we observed greater overlap of our 16p11.2 organoid data with organoid models of idiopathic ASD and other ASD genes (i.e., CHD8), than with 16p11.2 models from human lymphocytes or mouse brain. These results highlight the importance of using human-derived models for investigating neurodevelopmental disorders and suggest similarities between different genetic subtypes of ASD.

### The dosage of 16p11.2 CNV alters cell type composition of organoids

Transcriptomic and proteomic analyses identified molecular processes that were disrupted by the 16p11.2 CNV in the context of fetal brain development. Given complex cell type composition of human brain, these signatures may be in part related to effects of the CNV on cell-type composition of the organoids. To better understand how 16p11.2 dosage may impact cell type composition of organoids, we performed cell type enrichment analyses of organoid transcriptomes using single-cell RNA-seq (scRNA-seq) from the developing human neocortex [51].

**Fig. 4:**
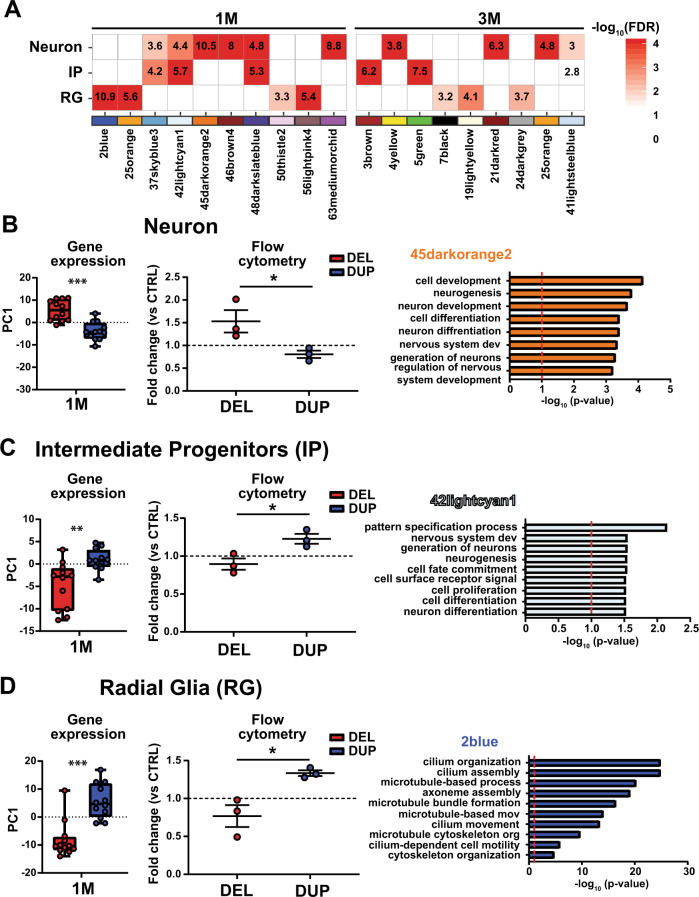
Cell type enrichment analyses of 1 M and 3 M organoid transcriptomes. **A** Cell type enrichment analyses of RNA-seq co-expression modules from 1 M and 3 M old organoids using cell types from scRNA-seq of fetal neocortex [51]. Only the modules significantly enriched in at least one of the three cell types are shown. “Neuron” category includes combination of excitatory and interneurons; IP intermediate progenitors, RG radial glia. Enrichment was evaluated using bootstrapping. *Z*-score was estimated by the distance of the mean expression of the target gene set from the mean expression of bootstrapping replicates and is shown inside each cell. P-values were corrected for multiple comparisons using FDR. **B**–**D** Left panel: principal component 1 (PC1) of enriched organoid modules at 1 M plotted by genotype. PC1 was computed for a union of genes from all modules significantly enriched in a specific cell type. All comparisons between DELs and DUPs are significant using t-test statistics. ***p < 0.001, **p < 0.01. Middle panel: Flow cytometry analysis of the dissociated cerebral organoids. Graphs represent quantification of the percentage of each cell population compared to CTRL. The data shown are representative of three independent experiments (*p ≤ 0.05). Right panel: GO terms for one representative most enriched module from 1 M dataset were obtained using g:Profiler [109].

We have previously demonstrated by scRNA-seq that at 1 M, organoids primarily consist of progenitor cells, with smaller fractions of glutamatergic neurons, glial cells, and intermediate progenitors (IPs) [36]. Here, we used recent scRNA-seq data from fetal human neocortex [51] to identify cell types significantly enriched in 1 M and 3 M old organoids. We observed significant enrichment of different cell types in co-expression modules for 1 M and 3 M organoids (Fig. 4A). Further analyses revealed that at 1 M DEL organoids were enriched in neuronal cell types (Fig. 4B), whereas DUPs were enriched in IPs and RG (Fig. 4C, D). In support of cell-type enrichment results, GO functions of most enriched modules reflected processes typically associated with corresponding cell types (Fig. 4B–D). For example, GO functions for 1 M *45darkorange2* “Neuron” cell type module included “neurogenesis”, “neuron development” and “neuron differentiation”. The GO functions for the “IP” *42lighcyan1* module included “pattern specification process”, “nervous system development”, and “generation of neurons”, along with differentiation- and proliferation-related functions that are relevant to primary function of IP in producing cortical neurons. The GO functions for “RG” *2blue* module captured cilium that is frequently found in radial glial cells, and microtubule-based processes. These results support a hypothesis that 16p11.2 copy number has a quantitative effect on the ratio of neurons to progenitor cells, with DUPs having a reduced proportion of neurons and DELs having an excess.

We sought to test this hypothesis directly by flow cytometry analysis on 1 M dissociated cerebral organoids (Materials and methods). Single-cell suspensions were labeled with NeuN, TBR2, and SOX2 for neurons, IP, and RG, respectively, and the percentages of positive cells were quantified (Fig. S22). We observed that the percentages of positively labeled cells between genotypes from flow cytometry experiments correlated well with single-cell enrichment analyses from gene expression. The percentage of NeuN^+^ cells was significantly higher in DELs compared to DUPs, suggesting an increase in the number of neurons, in agreement with cell-type enrichment analyses (Fig. 4B). In contrast, the percentages of TBR2^+^ and SOX2^+^ cells were significantly higher in DUPs, suggesting an increase in progenitor populations of RG and IP (Fig. 4C, D). These results point to potentially increased neuronal maturation in DEL organoids, and the opposite effect in DUP organoids. Overall, cell type enrichment and flow cytometry results provided further insight into cell-type composition of organoids and correlated with previous findings from ASD brain. For instance, excess neuron number has been observed as a hallmark of brain overgrowth in ASD patients during first year of life [52, 53], supporting “Neuron” cell type enrichment and macrocephaly phenotype in DELs.

### Increased neuronal maturation in 16p11.2 DEL organoids

**Fig. 5:**
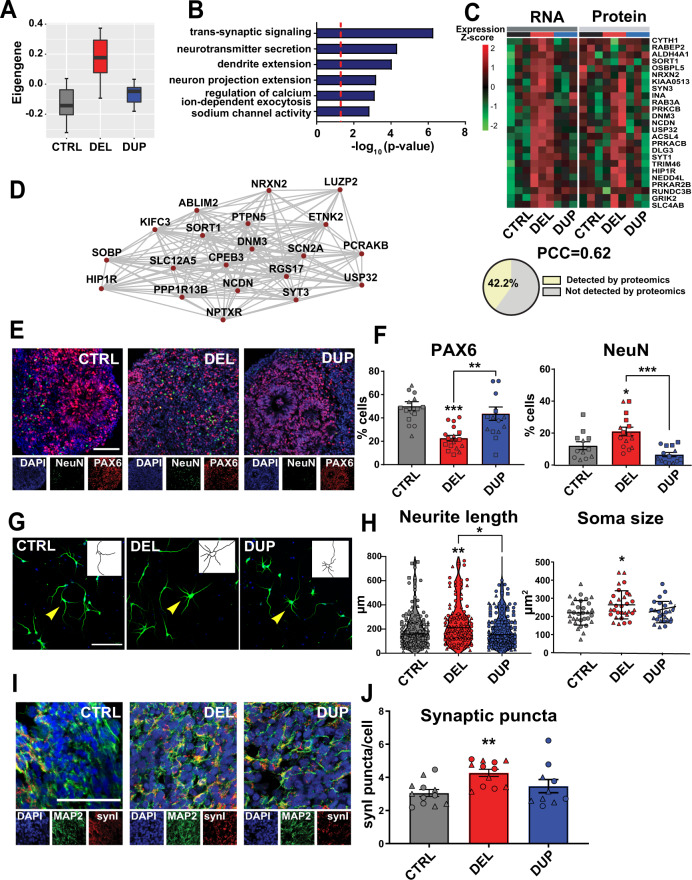
Altered neuronal maturation, morphology, and synaptic defects in 16p11.2 cortical organoids. **A**, **B** Module eigengene (**A**) and GO functional annotations (**B**) for *46brown4* transcriptomic module from 1 M organoids. The module eigengene was quantified from 12 data points (*n* = 12) corresponding to replicates derived from 3 patients (CTRL, DEL, or DUP), 2 clones per patient, and 2 replicates per clone. Two replicates were removed from CTRL before the analyses during outliers detection procedure (see Materials and methods). **C** Heat plot representing gene (RNA) and protein expression from *46brown4* module. Pearson correlation coefficient between RNA and protein expression and the proportion of genes whose protein products were also detected by proteomic profiling are shown below the plot. **D** Twenty top hub genes from *46brown4* co-expression module. Edges represent co-expression. **E** Representative images of 1 M organoid slices (CTRL, DEL, and DUP) immunostained with DAPI, PAX6, and NeuN. Scale bar: 100 µm. **F** Quantification of the percentage of positive cells for Pax6 (left) and NeuN (right) markers. Symbols represent organoids from the same differentiation batch, where batch is defined as CTRL, DEL, and DUP from one patient per genotype, one clone, and one replica. Data are presented as mean ± SEM (*n* = 2 patients per genotype, at least 4 organoids per patient). Significance was calculated using one-way ANOVA with Tukey’s multiple comparisons; ***p < 0.001, **p < 0.01, *p ≤ 0.05. The significance above bars represents comparison against CTRL. **G** Representative images of neurons from dissociated 1 M organoids immunostained with DAPI (blue) and MAP2 (green). Scale bar: 100 µm. Insets show a representative example of neurite tracing. Yellow arrows point to the neurons that were traced. **H** Quantification of total neurite length (left) and soma size (right). Symbols represent neurons derived from organoids from the same differentiation batch. Data is presented as mean ± SEM (*n* = 2 patients per genotype, at least 15 neurons per patient). Significance was calculated using one-way ANOVA with Tukey’s multiple comparisons; *p ≤ 0.05. The significance above bars represents a comparison against CTRL. **I** Representative images of 1 M organoid slices immunostained with DAPI, MAP2, and SynI. Scale bar: 50 µm. **J** Quantification of the total Synapsin I to estimate synaptic puncta. Symbols represent organoids from the same differentiation batch. Data are presented as mean ± SEM (*n* = 2 patients per genotype, at least 3 organoids per patient). Significance was calculated using one-way ANOVA with Tukey’s multiple comparison; ***p < 0.001. Significance above bars represents comparison against CTRL.

Transcriptome signatures and cell type specific analysis suggest that the 16p11.2 copy number could impact the proportion of neurons and neural progenitor populations. In addition, transcriptomic module *46brown4* in 1 M organoids is enriched in “Neuron” cell type, among other neuron-enriched modules (Fig. 4A). This module is significantly upregulated in DELs (Fig. 5A), and contained genes with neuronal and synaptic GO functions (Fig. 5B and Table S7). The expression levels of genes from this module highly correlate with corresponding protein expression (Pearson correlation coefficient (PCC) = 0.62), with 42.2% of genes within this module also detected by the proteomics (Fig. 5C). One of the high-confidence autism risk genes, *SCN2A* [54], is a highly connected hub in this module (Fig. 5D).

To validate these findings experimentally and to better understand the cellular basis of neuronal dysregulation in organoids, we quantified neural progenitors and neurons by immunohistochemistry in 1 M organoid slices. We observed significant depletion of neural progenitors (Pax6^+^) and significant enrichment of neurons (NeuN^+^) in DELs vs. CTRLs (Fig. 5E, F), and a mirror phenotype in DUPs vs. DELs, although DUPs vs. CTRLs did not reach statistical significance (Fig. 5F). These results were consistent with the flow cytometry results that pointed to the enrichment of DELs and depletion of DUPs in neurons (Fig. 4). These findings confirm that DEL organoids mature faster than DUP organoids, and that progenitor proliferation and differentiation dynamics could be disrupted by the 16p11.2 CNV. A higher number of neurons in DELs is also in agreement with the increased expression of synaptic genes that we have observed by the transcriptomic profiling. The DEL organoids also had decreased proliferation rate, most likely due to the depletion of the progenitor pool by 1 M (Fig. S23). Given an increased number of neurons in DELs at 1 M, it is plausible that increased proliferation prior to 1 M could lead to depletion of progenitors by 1 M. Indeed, accelerated proliferation of neural progenitors from iPSCs has been previously quantified at a much earlier time point than 1 M in other models [55], suggesting that at 1 M we may be capturing later or even terminal stages, at which progenitor pool in DELs has already been depleted. Cell cycle exit determined by the ratio of Edu^+^ and Ki67^−^ cells was not affected in DEL or DUP organoids at 1 M. The summary of this and all follow-up experiments by clones and replicate is shown in Table S12.

### Neuronal morphology and synaptic defects in 16p11.2 organoids

Neuronal maturation defects in organoids, along with differences in their size suggest that neuronal morphology could be affected by the 16p11.2 CNV. To test this hypothesis and to replicate previous observations from 2D neuronal cultures of the 16p11.2 carriers [22], we investigated neuron morphology by measuring neurite length and soma size in the dissociated 1 M organoids stained with MAP2 neuronal marker (Materials and methods). The total neurite length was increased in DEL vs. CTRL (*p* = 0.009, one-way ANOVA), and in DEL vs. DUP (*p* = 0.025, one-way ANOVA), with a trend for decreased neurite length in DUPs vs. CTRL that did not reach statistical significance. We also observed increased soma size in DEL organoids compared to CTRL (*p* = 0.034, one-way ANOVA) (Fig. 5G, H). These results suggest soma size and neurite length are phenotypes impacted by the 16p11.2 CNV in DELs.

Changes in neuronal morphology together with altered neuronal maturation could impact synaptogenesis in organoids. We therefore analyzed synaptic puncta by co-staining 1 M organoid slices with presynaptic marker Synapsin-I (SynI) and neuronal marker MAP2. We observed significant increase in the number of synaptic puncta normalized against the cell number in DEL organoids compared to CTRL (*p* = 0.008, one-way ANOVA) (Fig. 5I, J). This result is in agreement with the increased number of neurons, and with the upregulation of neuronal/synaptic transcriptomic module in DELs (Fig. 5A).

### Severe neuronal migration defects in 16p11.2 organoids

**Fig. 6:**
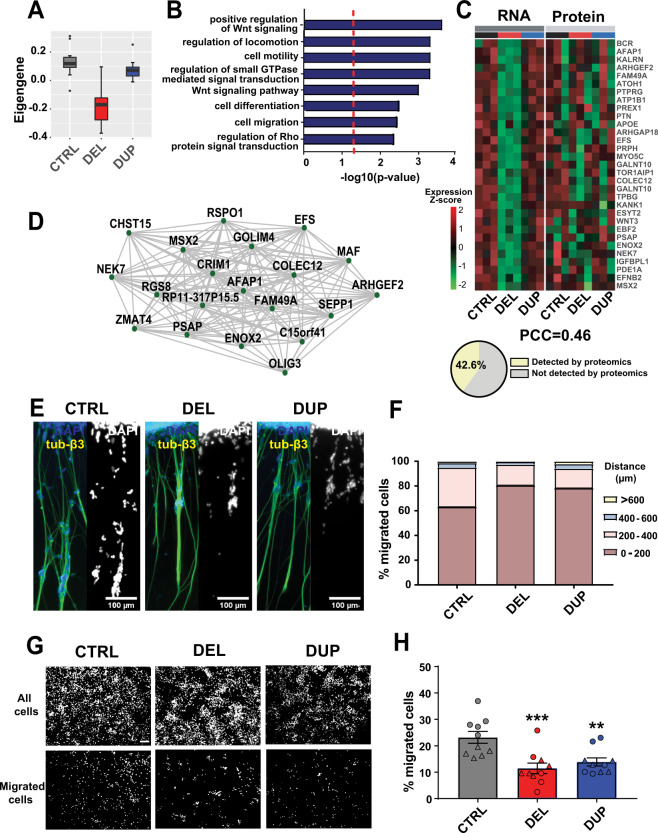
Neuronal migration defects in 16p11.2 cortical organoids. **A**, **B** Module eigengene (**A**) and GO functional annotations (**B**) for *22darkgreen* co-expression transcriptomic module from 1 M organoids. The module eigengene was quantified from 12 data points (*n* = 12) corresponding to replicate derived from 3 patients (CTRL, DEL, or DUP), 2 clones per patient, and 2 replicates per clone. Two replicates were removed from CTRL before the analyses during outlier detection procedure (see Materials and methods). **C** Heat plot representing gene (RNA) and protein (Protein) expression from *22darkgreen* co-expression module. Pearson correlation coefficient between RNA and protein expression and the proportion of genes whose protein products were also detected by proteomics are shown below the plot. **D** Twenty top hub genes from *22darkgreen* co-expression module. Edges represent co-expression. **E** Representative images of 1 M cortical organoids 72 h after attachment to Matrigel-coated plates, immunostained with DAPI and β-tubulin III. **F** Quantification of the percentages of migrating cells to a distance with 200 μm bins of displacement (*n* = 2 patients per genotype, at least 5 organoids per patient). The average of all experiments is shown, individual comparison for distance bins 0–200 and 200–400 together with statistical analyses using one-way ANOVA with Tukey’s multiple comparison is shown in Figs. S25 and S26. **G** Representative images of cells migrating from 1 M dissociated organoids in Boyden chamber experiment. Scale bar: 100 µm. Immunostaining was performed with DAPI. **H** Quantification of the percentages of migrated cells. Symbols represent cells derived from organoids from the same differentiation batch. Data are presented as mean ± SEM (*n* = 2 patients per genotype, at least 5 images per patient). Significance was calculated using one-way ANOVA with Tukey’s multiple comparisons; ***p < 0.001, **p < 0.01. The significance above bars represents a comparison against CTRL.

Neuronal migration during early fetal brain development could be one of the mechanisms that is disrupted in neurodevelopmental disorders [56]. Here, we observed that gene sets and modules involved in neuronal migration and locomotion were dysregulated across 16p11.2 transcriptomes and proteomes. For example, both DEGs (Fig. 2 and Table S5) and DEPs (Fig. 3 and Table S9) were enriched in neuron migration-related functions. In addition, the transcriptomic *22darkgreen* module from 1 M organoids was significantly downregulated in DELs and annotated with locomotion, migration, and motility GO functions (Fig. 6A, B and Table S7). Other highly enriched GO functions within this module included Wnt signaling (“regulation of Wnt signaling pathway”, “canonical Wnt signaling pathway”, “regulation of non-canonical Wnt signaling pathway”), a crucial pathway during early neurogenesis [57–59], that also impacts neuron migration [60]. In addition to *22darkgreen* module, Wnt signaling-related GO functions were also found in two iPSC transcriptomic modules (*4yellow* and *9magenta*), two additional 1 M organoids transcriptomic modules (*6red* and *42lightcyan*), four 3 M organoid transcriptomic modules (*2blue, 4yellow, 26darkorange*, and *13salmon*), and one proteomic 3 M organoids module (*1turquoise*) (see Table S7 and Table S11). Specifically, many genes from the Wnt signaling pathway were strongly downregulated in DEL organoids (Fig. S24).

Interestingly, “regulation of Rho protein signal transduction” and other Rho-related GO terms were also enriched in the migration-related *22darkgreen* module. RhoA is known to regulate neuronal migration, actin dynamics and neurite outgrowth during brain development [61–63]. Among the genes identified in this module, many are involved in the RhoA signaling. These genes include KANK1 that regulates actin-mediated migration and RhoA activity [64]; ApoE, a well-studied gene in the context of Alzheimer disease, that mediates cerebrovascular pericyte mobility through RhoA [65]; and PREX1, a guanine nucleotide exchange factor for the RHO family that functions as RAC 1 activator and is also involved in neuronal migration [66]. Interestingly, other guanine nucleotide exchange factors, such as SOS2, ARHGAP18, and ARHGEF2, known to be involved in cell migration and Rho signaling, are also present in this module. The expression levels of the genes from the *22darkgreen* module were positively correlated with the corresponding protein expression (PCC = 0.46) (Fig. 6C), with many network hub genes related to cytoskeletal, migration, and Wht/Rho signaling-related functions (Fig. 6D). This suggests that Wnt and RhoA signaling along with neuronal migration may be dysregulated by the 16p11.2 CNV. Specifically, the noncanonical Wnt signaling pathway that regulates actin cytoskeletal dynamics and cell migration [67] relies on the cross-talk between Wnt and Rho signaling [68, 69].

To investigate migration defects in the 16p11.2 organoids, we performed two orthogonal in vitro migration assays as previously described [33, 70]. First, we seeded organoids onto matrigel-coated plates, and quantified the number of migrated neurons and migration distance 72 h after seeding. Within the first 24 h after plating, protrusions of RG fibers from the organoid edges were observed. Then, neurons started to migrate along these fibers. While about 40% of neurons migrated to a distance of over 200 μm along the fibers in the CTRL organoids, only about 20% of neurons migrated to the same distance from the DEL or DUP organoids in this experiment (DEL vs. CTRL *p* = 0.038; DUP vs. CTRL *p* = 0.073, one-way ANOVA) (Fig. 6E, F and Fig. S25). Live imaging further confirmed that migration distance is shorter for both, DEL and DUP organoids (Fig. S26 and Movie S1). We verified by immunostaining that the fibers consist of neurites and RG bundles and that the migrating cells are neurons as opposed to neural progenitors (Fig. S27). The orthogonal Boyden chamber assay in the dissociated organoids (Materials and methods) demonstrated a lower proportion of migrating cells in DEL and DUP organoids, further validating migration defects by an independent method (Fig. 6G, H). These results suggest that neuron migration defects are observed in both, DEL and DUP organoids, and that these abnormalities could be present in 16p11.2 carriers during fetal brain development.

### Inhibition of RhoA activity rescues migration defects in 16p11.2 organoids

Rho signaling is one of the pathways enriched in the migration *22darkgreen* gene co-expression module (Fig. 6B). As we have hypothesized previously, 16p11.2 CNV may impact RhoA signaling through the KCTD13–Cul3 complex, because RhoA is a substrate of the Cul3 ubiquitin ligase, and KCTD13 serves as an adapter protein for Cul3 [45]. Dysregulation of RhoA has previously been observed in KCTD13 [71], TAOK2 [72], and recently in Cul3 [73] mouse models, supporting our hypothesis [45]. Thus, RhoA signaling may be one of the pathways contributing to the neuronal migration defects observed in organoids.

**Fig. 7:**
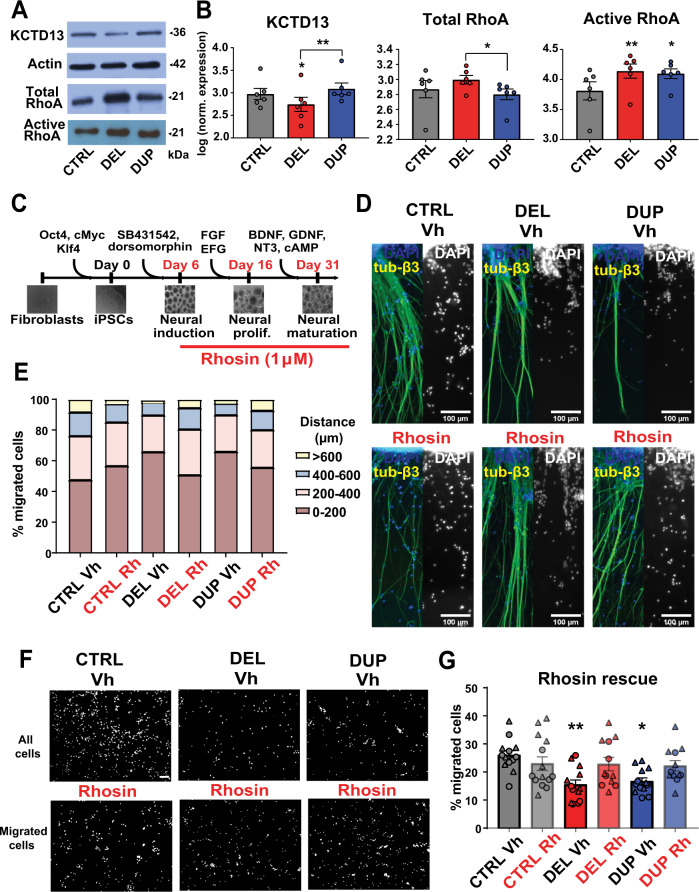
Active RhoA upregulation and Rhosin treatment rescue of neuronal migration deficits in 16p11.2 cortical organoids. **A** Representative images of Western Blot analysis of 1 M organoids for KCTD13, total RhoA, actin as loading control, and active RhoA (RhoA-GTP). All Western Blots used for quantification are shown in Fig. S28. **B** Densitometry analysis of Western Blot. Data are represented as mean ± SEM (*n* = 6 differentiation batches with at least one batch for each patient or control for KCTD13, total RhoA, and active RhoA). Significance was calculated using one-way ANOVA with Tukey’s multiple comparisons; **p < 0.01, *p ≤ 0.05. The significance above bars represents comparison against CTRL. **C** Schematic representation of constitutive Rhosin treatment of organoids during differentiation. **D** Representative images of 1 M vehicle- or Rhosin-treated cortical organoids 72 h after attachment to Matrigel-coated plates, immunostained with DAPI and β-tubulin III. **E** Quantification of the percentages of migrating cells to a distance with 200-μm bins of displacement (*n* = 2 patients per genotype, at least 5 organoids per patient). The average of all experiments is shown, individual comparison for distance bins 0–200 and 200–400 together with statistical analyses using two-way ANOVA with Tukey’s multiple comparisons are shown in Figs. S29 and S30. **F** Representative images of cells migrating from vehicle- or Rhosin-treated 1 M dissociated organoids in Boyden chamber experiment. Scale bar: 100 µm. Immunostaining was performed with DAPI. **G** Quantification of the percentages of migrated cells. Symbols represent cells derived from organoids from the same differentiation batch. Data are presented as mean ± SEM (*n* = 2 patients per genotype, at least five images per patient). Significance was calculated using two-way ANOVA with Tukey’s multiple comparisons; **p < 0.01, *p ≤ 0.05. Significance above bars represents comparison against CTRL Vh. Statistical analyses by batch are shown in Fig. S30.

We tested by Western Blot whether RhoA is dysregulated in 16p11.2 organoids (Fig. 7A, B and Fig. S28). KCTD13 protein level was significantly decreased in DELs vs. CTRLs (*p* = 0.018, one-way ANOVA), had an increasing but not significant trend in DUPs vs. CTRL (*p* = 0.25, one-way ANOVA), and had a significant opposing effect in DELs vs. DUPs comparison (*p* = 0.0013, one-way ANOVA), in agreement with the 16p11.2 CNV dosage. Although KCTD13 protein was not detected in our proteomics experiments, its levels in iPSC and organoids’ transcriptomes were also significantly dysregulated (decreased at <1% FDR in DELs vs. CTRLs in all datasets, and increased at <11% FDR in DUP vs. CTRL in all datasets) in the same direction as the 16p11.2 CNV dosage (Table S4), consistent with the Western Blot results. Total RhoA level, estimated from the Western Blot, was significantly changed in DELs vs. DUPs comparison (*p* = 0.01, one-way ANOVA), in agreement with its inverse trend with the 16p11.2 and KCTD13 dosage, as we have previously hypothesized [45]. However, active GTP-bound form of RhoA (RhoA-GTP) was significantly upregulated in organoids of both genotypes (DEL vs. CTRL *p* = 0.005, one-way ANOVA; DUP vs. CTRL *p* = 0.01, one-way ANOVA) (Fig. 6E–H). These results indicate that the active form of RhoA is significantly upregulated in both DELs and DUPs, which is consistent with the observed decreased neuron migration in both genotypes. RhoA overactivation was previously shown to lead to stalled neuronal migration in mouse cortex electroporated with spontaneously activated “fast-cycling” mutant RhoA [62]. These results suggest putative dysregulation of the RhoA signaling pathway, either directly by the 16p11.2 CNV, or by other genes outside of the *locus* that this CNV is impacting.

We then tested whether inhibition of RhoA activity could rescue neuron migration. We constitutively treated intact organoids with RhoA inhibitor Rhosin [74] starting from 6th day of differentiation until 30 days (Fig. 7C), and then performed migration experiments using two orthogonal methods as described above. The migration defects in both DEL and DUP were rescued by Rhosin to the levels indistinguishable from CTRL (DEL_Rh vs. CTRL_Vh *p* = 0.85, two-way ANOVA; DUP_Rh vs. CTRL_Vh *p* = 0.73, two-way ANOVA) (Fig. 7D, E, Figs. S29 and S30). The orthogonal Boyden chamber experiments replicated migration phenotype rescue by the Rhosin treatment (Fig. 7F, G and Fig. S30). However, the increased neurite length in DELs was not rescued by the Rhosin treatment (Fig. S31), likely due to a known Rhosin function in promoting neurite outgrowth, whereas inhibition of neurite outgrowth would be required to rescue this DEL phenotype. This suggests that RhoA signaling may be one of the pathways that are contributing to decreased migration but not to the neurite length and points to other potential pathways that may be involved in 16p11.2-impacted phenotypes.

## Discussion

Patient-derived and CRISPR/Cas9 genome-engineered isogenic brain organoids are becoming popular models for investigating molecular mechanisms underlying neurodevelopmental disorders [24, 75]. Given the lack of fetal brain tissues from ASD patients, there are numerous advantages in using brain organoids to model patient phenotypes. In the present study, we model molecular and cellular mechanisms of ASD risk attributable to rare DEL and DUP of the 16p11.2 *locus* using cortical organoids derived from skin fibroblasts of ASD patients with macrocephaly or microcephaly, respectively.

Organoid models of 16p11.2 CNV exhibit defects in neuronal maturation, migration, morphology, and synaptic abnormalities. Accelerated neuronal maturation in DEL, along with the impaired neuron migration in DEL and DUP, are novel mechanisms that have not been previously implicated in 16p11.2-linked autism. Our study makes a significant contribution to a mechanistic understanding of cellular and molecular processes that may be disrupted during early neocortical development in the 16p11.2 CNV carriers.

In this study, we perform an in-depth characterization of organoids’ transcriptomes and proteomes in parallel, from the same samples, at different developmental time points. This strategy provides the leverage for comparing phenotypes of DELs and DUPs at two levels of regulation, transcriptional, and translational. RNA-seq and quantitative proteomics identify DEGs and proteins, as well as co-expression modules that are impacted by the 16p11.2 CNV. We observe convergence at the level of biological processes and pathways for some functions, in addition to identifying unique GO terms from each approach.

At the molecular level, we observe perturbations of transcriptional programs associated with key processes involved in neurodevelopment. Transcriptional dysregulation of genes related to actin cytoskeleton and neuron migration were observed in 1 M organoids, whereas genes related to ion channels, synaptic signaling and nervous system development were observed in 3 M organoids (Fig. 2 and Fig. S10). This suggests that disruption of neural processes, which may not be apparent at the earlier developmental time point at 1 M, becomes more pronounced during later organoids maturation time point. The examples of the unique enriched GO terms are those related to translational dysregulation, observed in DUPs vs. CTRLs comparison at 3 M (Fig. S10 and Table S5). Many ribosomal subunit genes involved in translation initiation, mRNA catabolism, and protein transport were detected to be dysregulated in this analysis (Table S5).

The global transcriptional signatures of one to three month old organoids recapitulate those of the late mid-fetal human brain development (Fig. 1B), the most critical period for establishing network connectivity among 16p11.2 CNV genes [45]. This period was also implicated in ASD by other studies [43, 76]. Most importantly, transcriptional co-expression modules associated with neuronal/synaptic functions (Fig. 5A–D) and neuronal migration/Wnt/RhoA-signaling (Fig. 6A–D) identified in organoids were preserved at the proteomic level. Preservation of transcriptional signatures at the translational level further reinforces and validates our findings.

Our results are in agreement with other studies that include either organoids produced from idiopathic ASD individuals [34], or CHD8 organoids [50]. Significant overlap between DEGs and proteins from our study with DEGs from these studies is observed (Fig. 3E), suggesting shared signatures among different genetic subtypes of ASD. Importantly, synaptic gene co-expression module is also dysregulated in organoids from idiopathic ASD patients. The overlap of our data with DEGs from organoids engineered to knockdown CHD8, a top autism gene [50] is also apparent (Fig. 3E). However, no overlap was observed with 16p11.2 carriers’ lymphoblastoid cell lines [13], potentially emphasizing different transcriptional signatures captured by brain- and blood-derived models.

As observed previously in other brain diseases, organoid models can recapitulate patient’s microcephaly [23, 33, 37] and macrocephaly [50, 77] phenotypes. Here, we demonstrate that dosage changes of the same genetic variant could lead to opposite trends of organoids sizes (Fig. 1E and Fig. S7). In addition, we replicate altered neuronal morphology in DELs that was previously noted in 2D models [22]. Aberrant control of cell proliferation and excess neuron number has been previously hypothesized to cause early brain overgrowth in ASD patients [53, 78]. Consistent with this hypothesis, we observe excess of neurons and depletion of neural progenitors in DEL organoids, and a mirror phenotype in DUP organoids, in the DEL vs. DUP comparison. We also found decreased proliferation in 1-M-old DEL organoids. Interestingly, previous ASD studies either did not find differences in proliferation [22], or demonstrated accelerated proliferation of progenitors in ASD [34]. It is possible that decreased proliferation at 1 M in our study is a result of the accelerated progenitor proliferation at earlier time points that leads to premature depletion of neural progenitor pool by 1 M, and a subsequent decreased proliferation that we observed here. Further investigation of proliferation rates at various developmental time points (iPSCs, NPCs, and early maturation in organoids) is needed, and could uncover time-dependent mechanisms of proliferation defects in ASD.

One of the most important findings from our study is impaired cortical neuron migration in 16p11.2 organoids (Fig. 6E–H). We detected many DEGs and/or proteins that are involved in the regulation of neuronal migration from our DEG (Table S5) and DEP (Table S9) GO analyses. They included COL3A1 and GPR56, a ligand–receptor pair that regulates cortical development and migration through activation of the RhoA pathway [79]; LAMB1, a laminin subunit beta, implicated in Lissencephaly 5 (LIS5) [80] that mediates the attachment, migration, and organization of cells into tissues during embryonic development; and SRGAP2C, a SLIT-ROBO Rho GTPase activating protein 2C, that is involved in neuronal migration and dendritic spine maturation through inhibition of SRGAP2 [81]. We confirmed reduced migration in DEL and DUP organoids by two orthogonal methods, recordings from intact organoids (Fig. 6E, F), and experiments in dissociated organoids (Boyden chamber) (Fig. 6G, H). Previously, neuronal migration defects have also been observed in organoids derived from patients with lissencephaly [33], periventricular heterotopia [82], and in CHD8 deficient mice [83]. Our results suggest that aberrant neuronal migration may be present in the brains of subjects with 16p11.2 CNV during early neurogenesis. Notably, observations from post-mortem ASD brains show patches of disorganized cortical neurons that may not be migrating properly during early brain development [52].

Another important finding from our study is dysregulation of RhoA signaling by the 16p11.2 CNV. As noted above, many DEGs and DEPs with migration-related functions are also involved in RhoA signaling. For example, PHLDB2, also known as LL5beta, regulates microtubule and focal adhesion (FA) dynamics [84, 85] by forming a complex at the edges of the cell, and microtubule polymerization and disassembly of FA points are processes that are regulated by Rho GTPases [86]. MEF2C is a transcription factor that plays important role in neuronal survival and differentiation [87]. Dysregulation of this gene has been linked to neurodevelopmental disorders, including mental retardation [88, 89], and the transcription activity of MEF2C is regulated by RhoA signaling in a kinase cascade that also involves ERK6 [90]. Our observation linking RhoA activity in 16p11.2 organoids with defects in neuron migration is also consistent with neuron migration phenotypes observed in mouse conditional knockout of RhoA [62]. This model revealed that RhoA-depleted neurons migrated faster and reached cortical plate sooner than control neurons in the cerebral cortex of E14 mouse embryos. In addition, electroporation of spontaneously activated (“fast-cycling”) mutant of RhoA caused slower neuronal migration in the condition of activated RhoA. Thus, over-activation of RhoA stalls migration of neurons, in agreement with our results from the 16p11.2 CNV human organoid model. Our data are also in agreement with previous work that demonstrated the rescue of delayed neuronal migration by inactivation of RhoA or inhibition of ROCK, a direct target of RhoA [91–93]. Here, we show that inhibition of activated RhoA with Rhosin rescues the delayed migration in 16p11.2 DEL and DUP organoids.

There is a myriad of biological pathways that could be dysregulated by the 16p11.2 CNV in ASD [94, 95]. Due to its polygenic nature, with 29 genes within the *locus* and hundreds of genes impacted outside of the *locus*, as demonstrated here, genetic and epistatic interactions among these genes are likely responsible for neuroanatomical and cellular phenotypes observed in the patients and animal models [94, 96]. Investigation of this CNV creates apparent challenges in implicating a specific pathway, mostly due to the combinatorial and synergistic effect of multiple genes [97]. Rather, dysregulation of multiple pathways could lead to the observed cellular and molecular phenotypes. For example, a number of genes within 16p11.2 CNV (MAPK3, MVP and TAOK2), are involved in MAPK/ERK and phosphatidylinositol 3-kinase PI3K/AKT signaling pathways. These pathways, regulating cell cycle and proliferation of neural progenitors, were shown to be dysregulated in the 16p11.2 deletion mouse model [15], and are likely to also be impacted by this CNV. Here, we identified modules with genes and proteins involved in Wnt signaling, suggesting that 16p11.2 CNV may also impact this pathway. Given the cross-talk between Rho and Wnt signaling pathways [68, 98] that are both involved in the regulation of neuronal cytoskeleton during axon and dendrite growth, along with synapse formation, it is plausible that Wnt signaling could contribute to the neurite length phenotype in our 16p11.2 CNV organoid model. This possibility need to be investigated in the follow-up studies. Finally, as we demonstrated here, RhoA signaling is likely regulating neuronal migration in our 16p11.2 organoid model, and inhibition of RhoA activity rescues migration deficits. Thus, pleiotropy and epistasis of 16p11.2 CNV genes at the pathway level is a hallmark of its functional impact. Future studies using organoid models or fetal brain tissues from 16p11.2 CNV carriers are required to untangle the complexity of the phenotype-pathway relationships in ASD.

## Materials and methods

### Study design

The aim of this study was to investigate the impact of the autism-associated 16p11.2 CNV on early brain development using human-derived models. Specifically, our goal was to detect molecular pathways dysregulated by the dosage changes (i.e. DEL and DUP) of this CNV comprising 29 genes. To address this question, we generated cortical organoids derived from fibroblasts, reprogrammed into iPSCs, of 16p11.2 patients and healthy controls. We selected three patients of each genotype (three DEL, three DUP, and three CTRL), on the basis on the extreme head size phenotype, ASD diagnosis, and samples availability (Table S1). Due to limited availability, our study has been restricted to the males. To investigate changes in RNA and protein expression caused by the 16p11.2, bulk RNA sequencing and quantitative label-free TMT-MS proteomics experiments were performed. We profiled two clones per patient, and two replicas per clone, at three developmental stages (iPSCs, 1 and 3-month-old organoids), in 9 patients, for a total of 108 transcriptomes and 72 proteomes (iPSCs were not profiled by proteomics). The number of samples analyzed and the pipeline for the analysis are shown in Figs. S5 and S14. Changes in cell populations and neuron morphology were examined by immunostaining, and neuron migration phenotype observed in the transcriptomic/proteomics experiment was validated using in vitro experiments. Finally, RhoA and KCTD13 levels were examined using Western Blot. For all quantifiable experiments, investigators were blinded for the analyses. Different numbers of samples and replicates were used for different experiments, as specified in the Figure legends and Table S12. Raw data for all figures are provided in Table S13.

### Tissue collection

Skin fibroblasts of three patients with 16p11.2 DEL and three patients with 16p11.2 DUP were obtained from the Simons Searchlight https://www.sfari.org/resource/simons-searchlight/; formerly Simons Variation in Individuals Project or Simons VIP). Patients were selected based on fibroblasts availability, head circumference, ASD diagnosis, and were gender and age-matched (see detailed information about the patients in Table S1). De-identified patients tissue samples are distributed to Simons Investigators following approved IRB protocol to Simons Foundation through Columbia University Medical Center (PIs Drs. Gerald Fischbach and Wendy Chung). Collection and use for research of fibroblasts from three de-identified control individuals (CTRL) were approved by UCSD IRB. Skin fibroblasts were maintained in DMEM F-12 (Life Technologies) containing 10% FBS.

### Generation and maintenance of iPSCs

To generate iPSCs, skin fibroblasts were infected with Sendai virus vectors containing coding sequences of human OCT4, SOX2, KLF4, and c-MYC (Cytotune reprogramming kit, Thermo Fisher). Four days post-infection, fibroblasts were trypsinized to single cells, plated on the inactivated mouse embryonic fibroblast feeders, and cultured using a human embryonic stem cell medium (Gibco). After 3–4 weeks, iPSC clones were manually picked and propagated clonally on feeders. After 8–10 passages, iPSCs were transferred to a feeder-free system and grown on matrigel-coated dishes (Corning) in mTeSR1 media (StemCell Technologies). The cells were passaged by manually picking colonies.

### Quality control of generated iPSC clones

The generated iPSC clones were examined for genomic integrity by microarray genotyping. Parental fibroblasts and eight iPSC clones for each patient were genotyped using BeadChip Illumina microarray platform. CNVs were called using PennCNV (v1.0.3) [99] with default parameters. DEL or DUP were stitched as previously described [100, 101]. Briefly, variants were joined if the gap between two CNVs of the same type was less than 50% of the number of markers within the larger CNV. This rule was applied recursively until no more CNVs could be stitched. Only CNVs of over 100 kbp in size were retained for the subsequent analysis. In addition, if over 50% of the CNV overlapped with the regions that can confound CNV calling (such as assembly gaps, segmental DUP, centromeres, and telomeres), they were omitted from the analyses. We also removed CNVs if the number of markers supporting the call was less than 8 and/or if the PennCNV confidence score was less than 20. After applying these filters, we confirmed the presence of 16p11.2 DELs or DUPs in all fibroblast and iPSC clones. We then sought to remove those iPSC clones, for which CNV burden was significantly greater than that of parental fibroblasts. To compare iPSC clones and fibroblasts, we defined CNV burden as a total sum of base pairs that are copy number variables (excluding 16p11.2 CNV). For each patient, we defined the mean CNV burden as the CNV burden in fibroblasts, and standard deviation as the burden in all iPSC clones from the same patient. We then compared CNV burden between fibroblasts and iPSC clones for each patient, and discarded clones with the *Z*-scores > 1.5 SD. Most clones passed these filtering steps. Two iPSC clones with CNV burden closest to the parental fibroblasts of each patient were used for subsequent experiments.

### Generation of cortical organoids

To generate cortical organoids from iPSCs, we used the protocol described in Trujillo et al. [36]. Briefly, feeder-free iPSCs at passage 15 or later were fed daily with mTeSR1 for at least 7 days before differentiation. Colonies were dissociated using Accutase (Life Technologies) in PBS (1:1) for 10 min at 37 °C and centrifuged for 3 min at 100 × *g*. The cell pellet was resuspended in mTeSR1 supplemented with 10 μM SB431542 (SB, Tocris) and 1 μM Dorsomorphin (Dorso, R&D Systems). Approximately, 5 × 10^6^ cells were transferred to each well of a 6-well plate and kept in suspension under rotation (95 rpm) in the presence of 5 μM ROCK inhibitor (StemCell Technologies) for 24 h to form free-floating spheres. Then, the media was replaced with mTeSR1 for additional 48 h. After 72 h, Media1 [Neurobasal (Life Technologies) supplemented with Glutamax, 2% Gem21 NeuroPlex (Gemini Bio-Products), 1% N2 NeuroPlex (Gemini Bio-Products), 1% MEM nonessential amino acids (NEAA, Life Technologies), 1% penicillin/streptomycin (PS, LifeTechnologies), 10 μM SB and 1 μM Dorso] was used for maintenance for 7 days, with media changes every other day. Subsequently, Media1 was replaced with Media2 [Neurobasal with Glutamax, 2% Gem21 NeuroPlex, 1% NEAA and 1% PS] supplemented with 20 ng/mL FGF2 (Life Technologies) for additional 7 days. Then, Media2 was supplemented with both 20 ng/mL FGF2 and 20 ng/mL EGF (Life Technologies) and spheres were cultured for additional 7 days with media changes every other day. Next, organoids were transferred into Media3 [Media2 supplemented with 10 ng/mL BDNF, 10 ng/mL GDNF, 10 ng/mL NT-3 (all from Life Technologies), 200 μM l-ascorbic acid (Tocris), and 1 mM dibutyryl-cAMP (StemCell Technologies)] for another 7 days with media changes every other day. After 28 days, cortical organoids were maintained in Media2 for as long as needed, with media changes every 3–4 days. All organoids were generated, grown, and used for all experiments in the same plate with one DEL, one DUP and one CTRL (called a “batch” thereafter) to reduce batch effect from genotypes.

### Mycoplasma testing

All iPSC and organoid cultures were routinely tested for mycoplasma by PCR. Media supernatants (with no antibiotics) were collected, centrifuged, and resuspended in saline buffer. Ten microliters of each sample were used for Mycosplama testing using a commercially available LookOut Mycoplasma PCR Detection Kit (Sigma Aldrich) following the manufacturer’s instructions.

### Organoid size analyses

The diameter of individual organoids was measured using ImageJ software. Size measurements for organoid batches (1 DEL, 1 DUP, and 1 CTRL) followed a normal distribution, as verified with Prism software (GraphPad). For size comparison, the “large” group was defined as a proportion of organoids with size higher than 1 standard deviation (SD) within the batch; the “small” group as a proportion of organoids with a size lower than 1 SD within the batch, and the “medium” group comprised the remaining organoids. The proportions for each batch were averaged for final quantification.

### Immunofluorescence staining

Cortical organoids were fixed in 4% paraformaldehyde (PFA) overnight. Next morning they were washed in PBS, transferred to a 30% sucrose solution, and kept at 4 °C. After the 3D structures sink, they were transferred into Tissue-Tek OCT medium (Sakura). Subsequently, 20 μm thick sections were obtained using a cryostat. For immunostaining of iPSC clones, cells were grown directly on Matrigel-coated coverslips.

Slides containing organoid slices were air-dried and then washed with PBS to remove excess OCT. Permeabilization and blocking was performed with 4% fetal bovine serum (FBS, Gibco), 0.1% Triton X-100 (Sigma Aldrich) diluted in PBS for one hour at room temperature. The slides were then incubated overnight at 4 °C with primary antibodies diluted in a solution containing 4% FBS and 0.1% Triton X-100. PBS was used to wash the primary antibodies and the slides were incubated with secondary antibodies in solution containing 4% FBS for 1 h at room temperature. The following primary antibodies were used for immunostaining: NANOG (goat, 1:500, R&D Systems), Oct4 (mouse, 1:500, Abcam), Tra-1-60 (mouse, 1:500, Abcam), Lin28 (rabbit, 1:500, Abcam), PAX6 (mouse, 1:300; DSHB), NeuN (mouse, 1:500, Millipore), NeuN (rabbit, 1:500, Cell Signaling), MAP2 (chicken, 1:2000; Abcam), Ki67 (rabbit, 1:1000, Abcam), β-tubulin III (mouse, 1:500, Abcam), Synapsin I (rabbit, 1:500, Abcam), SOX2 (rabbit, 1:500, Abcam). Alexa Fluor Dyes (Abcam) were used at 1:1000 dilution as secondary antibodies. Nuclei were visualized with Hoechst 33258 (1:25,000, Life Technologies). EdU was visualized using the Edu Staining Kit (Abcam) following manufacturer’s instructions. Slides were mounted using ProLong Gold antifade reagent (Invitrogen) and analyzed under a fluorescence microscope (Leica SP8). Image analysis was performed with ImageJ software. All cells expressing a particular marker were counted on sections and normalized to the total number of cells.

### Flow cytometry analysis

Cortical organoids were first dissociated to a single cell suspension. Then, cells were fixed for 15 min in ice-cold 1% PFS in PBS, washed twice with cold PBS, and incubated for 2 h at room temperature with primary antibodies for specific cell markers (NeuN, SOX2, TBR2; Abcam) at 1:500 dilutions. Following a washing step with PBS, cells were incubated with 1:500 Alexa 488-conjugated antibodies (Abcam) for 20 min at room temperature. Analysis was performed on a flow cytometer (Accuri C6, BD Biosciences). Fifty thousand events were acquired for each sample with fluorescence measured in logarithmic scale. Background fluorescence was measured using cells labeled only with secondary antibody alone and used to set the gating parameters between positive and negative cell populations. Forward and side light-scatter gates were used to exclude cell aggregates and small debris. Data were analyzed using the FlowJo software and plotted in a histogram format. All histograms were smoothed by the software. Fluorescence gates were set below 2% of blank histogram and events corresponding to a fluorescence signal exceeding this percentage were considered as positive events.

### Cell migration assay

For the in vitro migration assay, intact organoids were seeded in Matrigel-coated 24-well plates (3–4 organoids per well), using Media2. Organoids were allowed to attach to the bottom of the plate for 24 h, then media was replaced with fresh Media2 carefully not to disrupt organoids attachment. After 72 h, immunostaining was performed. Images were taken using EVOS FL Cell Imaging System. Cell counting and image analysis were performed with ImageJ software.

For live-imaging, intact organoids were seeded in Matrigel-coated p35 glass-bottom culture dishes (Greiner). After 24 h, pictures were taken every 15 min using a LEICA SP8 microscope. Videos were mounted using LEICA SP8 software.

### Organoids dissociation

Cortical organoids were dissociated into single-cell suspension using Accumax (Sigma Aldrich) for 30 min at 37 °C with rotation (95 rpm). Then, organoids were disaggregated using a 1000 µl pipette tip, incubated for another 10 min at 37 °C in suspension with rotation (95 rpm), and centrifuged for 3 min at 100 × *g*. The cell pellet was resuspended in Media2 containing 5 μM of ROCK inhibitor, filtered through a 100 µm mesh (Gibco) and centrifuged again for 3 min at 100 × *g*. To further remove un-dissociated organoid tissue, the procedure was repeated but with filtering through the 40 µm mesh (Gibco). Cells from suspension were counted using a Bio-Rad TC10 Cell Counter.

### Boyden chamber migration assay

Approximately, 3 × 10^5^ cells from the dissociated organoids were seeded on top of a Millicell Cell Culture 8 µm Insert (Millipore) in 24-well plates. The bottom of the Cell Culture Insert was filled with 500 µl of Media2 supplemented with 20 ng/mL of FGF2 and 10 ng/mL of BDNF as chemo-attractants. Cells were left to freely migrate for 24 h, then washed with PBS and fixed with 4% PFA for immunostaining.

After immunostaining, images were taken using EVOS FL Cell Imaging System, visualizing all cells on the Cell Culture Inserts. Then, cells on the top of the Cell Culture Insert were removed using a cell scrapper. After three washes with PBS, only cells on the bottom of the insert were visualized. Cell counting was performed with ImageJ software.

### Pharmacological treatment of cortical organoids

For phenotype rescue experiments, organoids were grown in Rhosin-treated media. Rhosin (Tocris) was added to the media during the differentiation stage starting from day 6 (Rhosin was first added to second Media1 to the final concentration of 1 µM). The same amount of Rhosin was added during all subsequent media changes. The organoids were grown for 1 month, at which cell migration assays were carried out. An equivalent amount of vehicle (0.1% Dimethylsulfoxide, DMSO) was added to grow untreated CTRL, DEL, and DUP organoids.

### Neuronal morphology analysis

Cortical organoids were dissociated and approximately 3 × 10^5^ cells per well were seeded on a 24-well plate coated with poly-ornithine (Sigma Aldrich) and Laminin (Invitrogen). Media2 was changed after 24 h to remove the ROCK inhibitor, and a second media change was performed after 3 days. Cells were fixed seven days after seeding for immunostaining. Images were taken using a LEICA SP confocal microscope and analyzed with ImageJ software. For soma area calculation, the perimeter of the MAP2-positive cell body was manually outlined and measured. For total dendrite length, each dendrite or its branch was traced separately, and the dendrite length was calculated by adding individual lengths for every neuron.

### Synaptic puncta quantification

Three-channel z-stack images of organoid slices were taken using an oil-inverted 60× objective. Then, an average projection image of each stack was generated. At least six randomly selected image fields for each genotype from two different batches were used for quantification of number of synapses with Synapsin I staining. Only puncta overlapping MAP2-positive processes were scored. The number of Synapsin I puncta was quantified using a plug-in Puncta Analyzer from the Fiji analysis software platform [102].

### Western blot

Cortical organoids from a quarter to a half of a well were washed once with ice-cold PBS (w/o Ca^2+^/Mg^2+^). Proteins were extracted using lysis buffer (20 mM Tris, pH 7.4, 140 mM NaCl, 10% glycerol, 2 mM EDTA, 1 mM EGTA, and 1% Triton X-100) supplemented with EDTA-free Complete protease inhibitor cocktail (Roche) and Phosphatase Inhibitor cocktail (Sigma Aldrich). The suspension was centrifuged at 16,000 × *g* at 4 °C for 30 min, and supernatants were collected. Protein concentration was quantified by a modified Lowry assay (DC protein assay; Bio-Rad). Cell lysates were resolved by Sodium dodecyl-sulfate polyacrylamide gel electrophoresis and transferred onto polyvinylidene fluoride Immobilon-P membranes (Millipore). After blocking with 1× TBS, 0.1% Tween-20 containing 5% nonfat dry milk for 1 h at room temperature, membranes were first probed with primary antibodies, and then after 1 h of incubation with corresponding peroxidase-conjugated secondary antibody (Abcam). Membranes were developed using the EZ-ECL chemiluminescence detection kit (Denville Scientific). The following primary antibodies were used as follows: anti-KCTD13 (1:500; Atlas Antibodies), anti-RhoA (1:1000; Cell Signaling), and anti-β-actin (1:5000; Sigma Aldrich) as a loading control. Quantification was performed by densitometry with ImageJ software.

### RNA isolation for RNA-Seq and qPCR

Total RNA was extracted from undifferentiated iPSCs or cortical organoids at 1 and 3 months of differentiation. Two clones from each patient were used for RNA isolation, for each time point analyzed. Total RNA was extracted using the QIAGEN RNAeasy isolation kit (QIAGEN) following manufacturer’s instructions. RNA sequencing was performed using the same input amount of total RNA for each sample. RNA samples were ribodepleted using Ribo-Zero rRNA Removal Kit (Illumina) and library preparation was performed using the TrueSeq Stranded Total RNA kit for Illumina Sequencing according to the manufacturer’s instructions. Paired-end RNA sequencing with 100 bp reads was performed on an Illumina HiSeq4000 to an average depth of 40 M reads per sample.

For qPCR experiments, cDNA was synthesized, starting from 100 ng of total RNA with the SuperScript III First-Strand Synthesis kit and random hexamers (Invitrogen). qPCR was performed using the CFX96 Touch^™^ Real-Time PCR Detection System (Bio Rad) using Power SYBR Green PCR Master Mix (Applied Biosystems). HPRT1 and β-actin were used as housekeeping genes for normalization. Fold change in expression was calculated using the ΔΔCt method.

### RNA-sequencing data processing pipeline

All 108 FASTQ files (36 iPSC, 36 one-month organoids, and 36 three months organoids paired-end fastq) (Fig. S5) were run through a unified RNA-Seq processing pipeline. Pipeline source code can be found on https://github.com/IakouchevaLab/16p11.2. All fastq were trimmed for adapter sequence and low base call quality (Phred score < 30 at ends) using Cutadapt (v1.14). Trimmed reads were then aligned to the GRCH37.p13 (hg19) reference genome via STAR (2.5.3a) using comprehensive gene annotations from Gencode (v19) (Fig. S8). Gene-level quantifications were calculated using RSEM (v1.3). Quality control metrics were calculated using RNA-SeQC (v1.1.8), featureCounts (v1.6.), PicardTools (v2.12), and Samtools (v1.3) (Fig. S8 and Table S2).

### RNA-Seq quality control and normalization

Expected counts were compiled from gene-level RSEM quantifications and imported into R for downstream analyses. Expressed genes were defined as genes with TPM > 0.5 in at least 80% of samples from each genotype (CTRL, DEL, or DUP). A total of 15,788; 13,348, and 13,723 expressed genes from iPSC, 1 M old organoids, and 3 M old organoids, respectively, were used in the downstream analysis. Outliers were defined as samples with standardized sample network connectivity *Z* scores < −2 [103], and were removed (Fig. S8). Highly variable genes between clones from the same individual were filtered out using the Variance Partition (v3.5) R package [104].

### Covariate selection

We compiled a set of 197 RNA-Seq quality control metrics from the outputs of cutadapt, STAR, RNA-SeQC, featureCounts and PicardTools (CollectAlignmentSummaryMetrics, CollectInsertSizeMetrics, CollectRnaSeqMetrics, CollectGcBiasMetrics, MarkDuplicates) for each group of samples (iPSCs, 1-M-old organoids and 3-M-old organoids) (Table S2 and Fig. S8). These measures were summarized by the top principal components, which explained the majority of total variance of each group (Fig. S9). Batch effects and possible hidden confounding factors were detected using the Surrogate Variable Analysis (SVA) [105]. Multivariate adaptive regression splines (MARS) implemented in the earth package in R was used to determine which covariates to include in the final differential expression model (Fig. S9). The potential covariates included: run/batch, RIN, clone, seqPCs, and SVs (Fig. S9). These covariates were inputted into the MARS model along with gene expression data (limma voom normalized, centered, and scaled). The model was run using linear predictors and otherwise default parameters. MARS selected SV1 as a covariate for iPSC, SV1–SV5 as covariates for 1-M old organoids, and SV1–SV6 as covariates for 3-M-old organoids (Fig. S9).

### Differential gene expression

Differential gene expression analyses were performed using limma-voom with “duplicateCorrelation” function to account for duplicate samples (clones and replicas) from the same individuals, and to avoid pseudo-replication in the analyses [47]. Covariates were included as fixed effects in the model. The biomaRt [106, 107] package in R was used to extract gene names, gene biotypes and gene descriptions. Differential gene expression analyses were performed using all three datasets (CTRL, DEL, and DUP) for all time points. The volcano plots for iPSCs and 3 M organoids are shown in Fig. S10, and DEGs from these datasets are listed in Table S4.

### WGCNA on RNA-seq data

We used weighted gene co-expression network analysis (WGCNA) [48] to define modules of co-expressed genes from RNA-seq data (Fig. S11). All covariates except for genotype at the 16p11.2 locus were first regressed out from the expression datasets. The co-expression networks and modules were estimated using the blockwiseModules function with the following parameters: corType = bicorr; networkType = signed; pamRespectsDendro = F; mergeCutHeight = 0.1. Some parameters were specific for each dataset. For iPSC data: power = 14; deepSplit = 0; minModuleSize = 100. For 1 M old organoid data: power = 16; deepSplit = 2; minModuleSize = 50. For 3 M old organoid data: power = 19; deepSplit = 2; minModuleSize = 70. The soft threshold power was chosen to correspond to the scale-free topology fit index of 0.8 or higher (Fig. S11). Module eigengene-genotype associations were calculated using a linear mixed-effects model, using a random effect of individual, to account for multiple clones and replicas derived from the same patient. P-values were FDR-corrected to account for multiple comparisons. Genes within each module were prioritized based on their module membership (kME), defined as correlation to the module eigengene. For selected modules, the top hub genes are shown in Figs. 5B and 6B. Module preservation was tested using the modulePreservation function from the WGCNA package in R.

### Enrichment analysis of GO functions and literature curated gene sets

Enrichment for GO (Biological Process and Molecular Function) was performed using gProfileR R package [108, 109]. Background was restricted to the expressed set of genes by group (iPSC—15,757, 1 M organoids—11,880, and 3 M organoids—13,555). 16p11.2 genes were excluded from GO analyses. Only DEGs with <10% FDR were selected for GO analyses. An ordered query was used, ranking genes by FDR-corrected *p*-value for DGE analyses and by kME for WGCNA analyses.

Enrichment analyses were also performed using several established, hypothesis-driven gene sets including syndromic and highly ranked (1 and 2) genes from SFARI Gene database (https://gene.sfari.org/database/gene-scoring/); pre- and post-synaptic genes from SynaptomeDB [110]; genes with loss-of-function intolerance (pLI) > 0.99 as reported by the Exome Aggregation Consortium [111]; highly constrained genes [112]; FMRP targets [113] and CHD8 targets [114]. Statistical enrichment analyses were performed using permutation test. One thousand simulated lists with a similar number of genes, gene length distribution, and GC-content distribution as the target gene list were generated, and the overlaps between each of the simulated lists and the hypothesis-driven gene sets were calculated to form the null distribution. Significance p-value was calculated by comparing the actual overlap between target list and hypothesis-driven gene sets to the null distribution. All results were FDR-corrected for multiple comparisons.

### Cell type enrichment analysis

Cell-type enrichment analysis for each co-expression module was performed using the expression weighted cell type enrichment package in R [115]. Cell type-specific gene expression data was obtained from single-cell sequencing (scRNA-seq) studies of the human fetal neocortex [51]. The specificity metric of each gene for each cell type was computed as described [115]. “Neuron” cell type includes a union of ExcNeu (excitatory neurons) and IntNeu (interneurons). Enrichment was evaluated using bootstrapping. *Z*-score was estimated by the distance of the mean expression of the target gene set from the mean expression of bootstrapping replicates. *p*-Values were corrected for multiple comparisons using FDR.

### CoNTExT analyses

Regional and temporal identify of organoids was assessed using CoNTExT [40] (https://context.semel.ucla.edu/).

### Sample preparation, protein identification, and quantification by TMT-MS

TMT-MS experiments were performed on the organoid samples from the same well as those used for RNA-seq, by splitting the content of each well into two approximately equal amounts (Fig. S14). Organoids were lysed in 100 mM TEAB with 1% SDS, protease inhibitor cocktails (Sigma) and PhosSTOP (Sigma) by 2–3 times of brief probe sonication and then centrifuged at 18,000 × *g* for 15 min at 4 °C. Supernatants were reduced (10 mM TCEP at 55 °C for 20 min) and alkylated (50 mM chloroacetamide at room temperature in the dark for 20 min), and then MeOH/CHCl_3_ precipitation was performed. Pellets were dissolved by adding 6 M urea in 50 mM TEAB, and then LysC/Tryp (Promega) was added by 1:25 (w/w) ratio to the peptides. After 3–4 h incubation at 37 °C, the reaction mixture was diluted with 50 mM TEAB for urea to be less than 1 M. After the o/n digestion, peptide concentration was estimated by colorimetric peptide BCA assay (Thermo), and the peptides were labeled with TMT 10-plex reagents (Thermo) for one hour, followed by 15 min quenching with hydroxylamine according to the manufacturer’s protocol. Equal amount of reaction mixtures for each channel were pooled together and dried using SpeedVac.

Since the total number of samples exceeded the maximum number of TMT channels, samples were divided into multiple sets (one replicate per set). To compare and normalize different sets of TMT-labeled samples, pooled peptides were labeled with 131 N and 131 C as duplicates, and these samples were commonly included in all sets within each age (1 M and 3 M old organoids) set. A total of 100 μg of peptides were fractionated using Pierce^™^ High pH reversed-phase peptide fractionation kit (Thermo) and then dried in SpeedVac. Dried peptides were dissolved with buffer A (5% acetonitrile, 0.1% formic acid), and half of each fraction was injected directly onto a 25 cm, 100 μm-ID column packed with BEH 1.7 μm C18 resin (Waters). Samples were separated at a flow rate of 300 nL/min on nLC 1000 (Thermo). A gradient of 1–25% B (80% acetonitrile, 0.1% formic acid) over 200 min, an increase to 50% B over 120 min, an increase to 90% B over another 30 min and held at 90% B for a final 10 min of washing was used for 360 min total run time. The column was re-equilibrated with 20 μL of buffer A prior to the injection of the sample. Peptides were eluted directly from the tip of the column and nanosprayed directly into the mass spectrometer Orbitrap Fusion by application of 2.8 kV voltage at the back of the column.

Fusion was operated in a data-dependent mode. Full MS1 scans were collected in the Orbitrap at 120k resolution. The cycle time was set to 3 s, and within this 3 s the most abundant ions per scan were selected for CID MS/MS in the ion trap. MS3 analysis with multi-notch isolation (SPS3) [116] was utilized for detection of TMT reporter ions at 60k resolution. Monoisotopic precursor selection was enabled, and dynamic exclusion was used with an exclusion duration of 10 s. Tandem mass spectra were extracted from the raw files using RawConverter [117] with monoisotopic peak selection. The spectral files from all fractions were uploaded into one experiment on Integrated Proteomics Applications (IP2, Ver.6.0.5) pipeline. Proteins and peptides were searched using ProLuCID [118] and DTASelect 2.0 [119] on IP2 against the UniProt *H. sapiens* protein database with reversed decoy sequences (UniProt_Human_reviewed_05-05-2016_reversed.fasta). Precursor mass tolerance was set to 50.0ppm, and the search space allowed all fully-tryptic and half-tryptic peptide candidates without limit to internal missed cleavage and with a fixed modification of 57.02146 on cysteine and 229.1629 on N-terminus and lysine. Peptide candidates were filtered using DTASelect parameters of -p 2 (proteins with at least one peptide are identified) -y 1 (partial tryptic end is allowed) –pfp 0.01 (protein FDR < 1%) -DM 5 (highest mass error 5 ppm) -U (unique peptide only). Quantification was performed by Census [120] on IP2.

### Differential protein expression

Proteomics data was first summarized to peptide level by adding up the intensities of constituting spectra. Quantitation results from different TMT runs were merged and normalized using the pooled samples channel which was present in all runs. For each peptide, multiple measurements from the same subject were collapsed to one measurement by taking the median of all measurements. The data was then log2 transformed. Differential protein expression was calculated by fitting a linear mixed-effects model for each protein, using the lme4 package in R [121]. Genotype was included as fixed effect in the model. We included a random effect term for each peptide to account for the fact that different peptides from the same protein are not entirely independent. Significance *p* values were calculated using lmerTest package in R [122]. The resulting *p*-values were FDR-corrected using the Benjamini–Hochberg method to control for multiple comparisons. The volcano plots for 3 M organoids are shown in Fig. S15, and DEPs from these datasets are listed in Table S8. GO analyses was performed as described above for DEGs, with the background restricted to the expressed set of proteins by group (1 M organoids—6113, and 3 M organoids—5470).

### Weighted protein co-expression network analysis

Proteomics data was first summarized to protein level by adding up the intensities of constituting peptides. Quantitation results from different TMT runs were merged and normalized using the pooled samples channel which was present in all runs. The merged data was then log2 transformed. Outlier samples detection, highly variable proteins removal, surrogate variables calculation and covariates selection were subsequently performed using the same methods as described for RNA-seq data processing. All covariates except for genotype at the 16p11.2 locus were first regressed out from the expression datasets. Protein co-expression networks and modules were estimated using the blockwiseModules function with the following parameters: corType = bicorr; networkType = signed; pamRespectsDendro = F; mergeCutHeight = 0.1. Some parameters were specific for each dataset. For 1 M old organoid data: power = 13; deepSplit = 3; minModuleSize = 40; and for 3 M old organoid data: power = 17; deepSplit = 2; minModuleSize = 10. The soft threshold power was chosen to correspond to the scale-free topology fit index of 0.8 or higher (Fig. S16). Module eigengene-genotype associations were calculated as described for the RNA-seq WGCNA. Module preservation was tested using the modulePreservation function from the WGCNA package in R.

### Quantification and statistical analyses

The statistical analyses for above experiments were performed using Prism software (GraphPad). In most experiments, when comparison of several genotypes against each other (CTRL, DEL, and DUP) was required, the one-way ANOVA with Tukey correction to account for multiple comparisons was used. In all Rhosin rescue experiments, two-way ANOVA with Tukey correction to account for multiple comparisons was used. Statistical tests used and exact values of *n* are described in Figure legends. Significance was defined as *p* < 0.05(*), *p* < 0.01(**), or *p* < 0.001(***). Blinded measurements were performed for any comparison between control and 16p11.2 genotypes. The samples used for each type of experiments are shown in Table S12.

## Supplementary information

Supplementary Figures

Suppl Movie 1

Table S1

Table S2

Table S3

Table S4

Table S5

Table S6

Table S7

Table S8

Table S9

Table S10

Table S11

Table S12

Table S13

Suppl Figures and Tables legends

## Data Availability

Source RNA-seq data is available at GEO repository accession number GSE142174. Source proteomics data is available from the public repository MassIVE (Mass Spectrometry Interactive Virtual Environment), a part of the ProteomeXchange consortium, with the identifier MSV000084727 (and PXD016855 for ProteomeXchange).

## Materials availability

New iPSCs lines generated in this study will be deposited after publication to Rutgers repository by the Simons Foundation for Autism Research that funded this study.

## Code availability

The code used for the RNAseq and proteomics analysis generated during this study is available from GitHub (https://github.com/IakouchevaLab/16p11.2).
