# Supplementary material for: Cortical organoids model early brain development disrupted by 16p11.2 copy number variants in autism
Source: Mol Psychiatry. 2021 Aug 26;26(12):7560–80. doi: 10.1038/s41380-021-01243-6 (PMC8873019; doi:10.1038/s41380-021-01243-6)

Supplementary Fig. S1

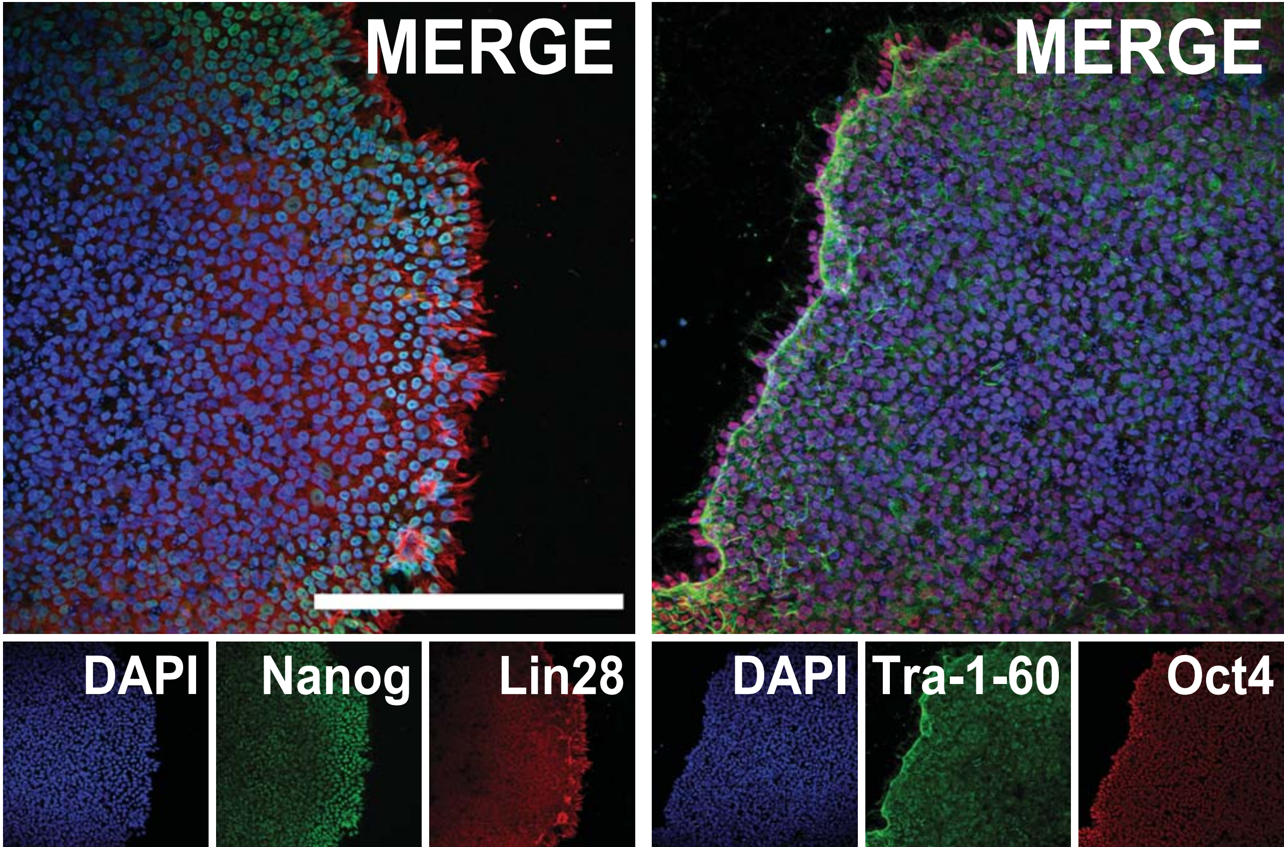

Supplementary Fig. S2

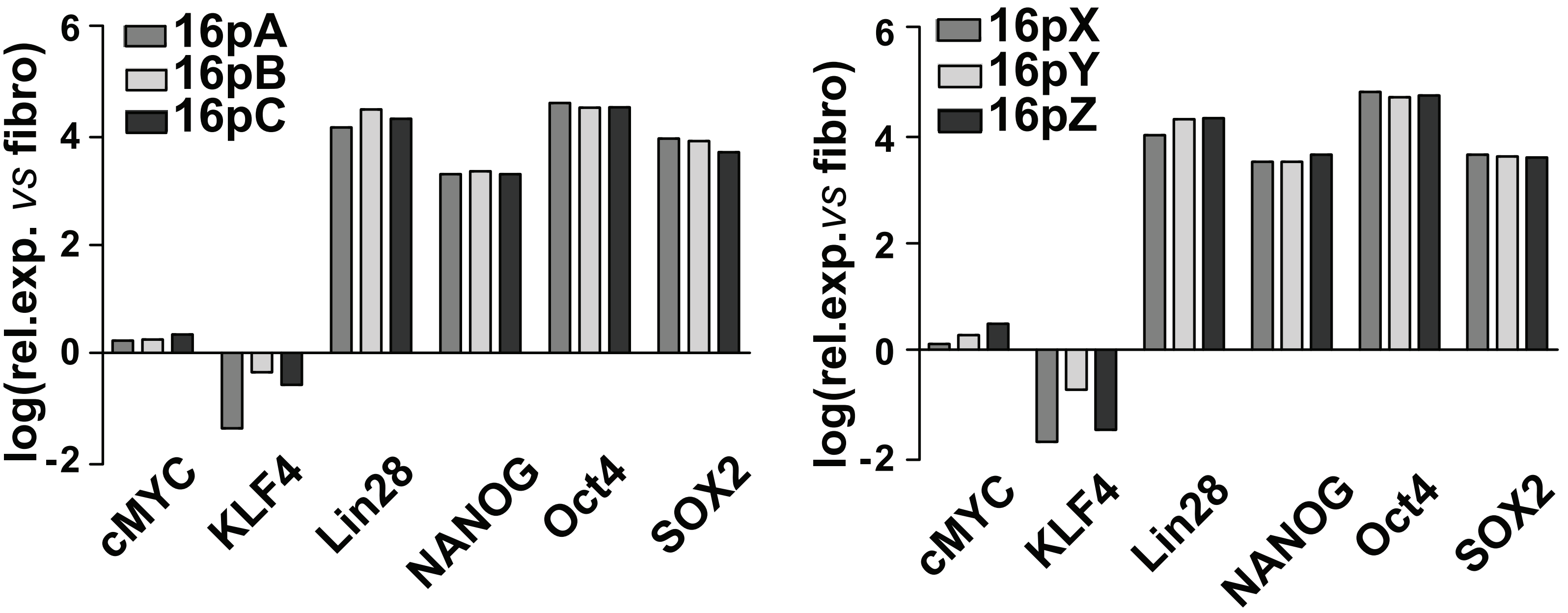

Supplementary Fig. S3

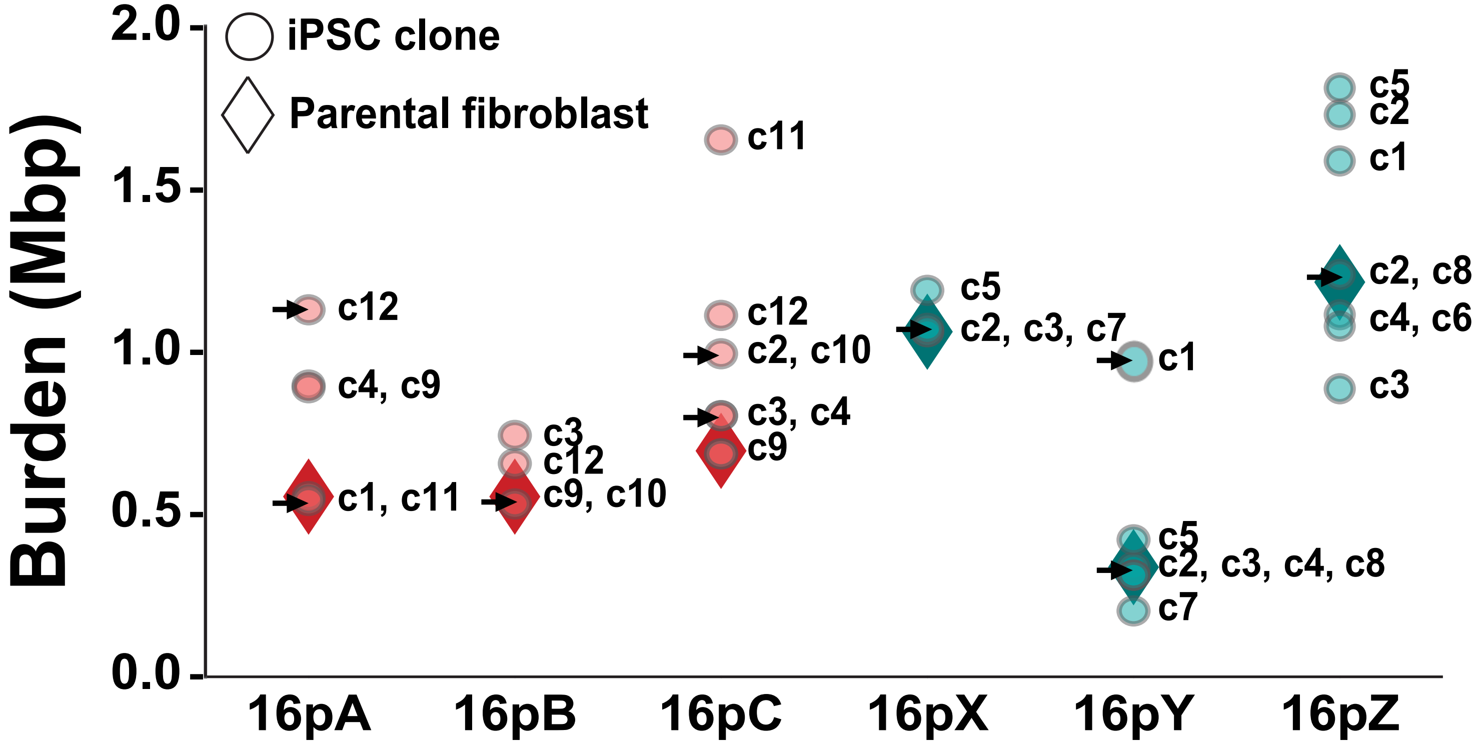

Supplementary Fig. 4

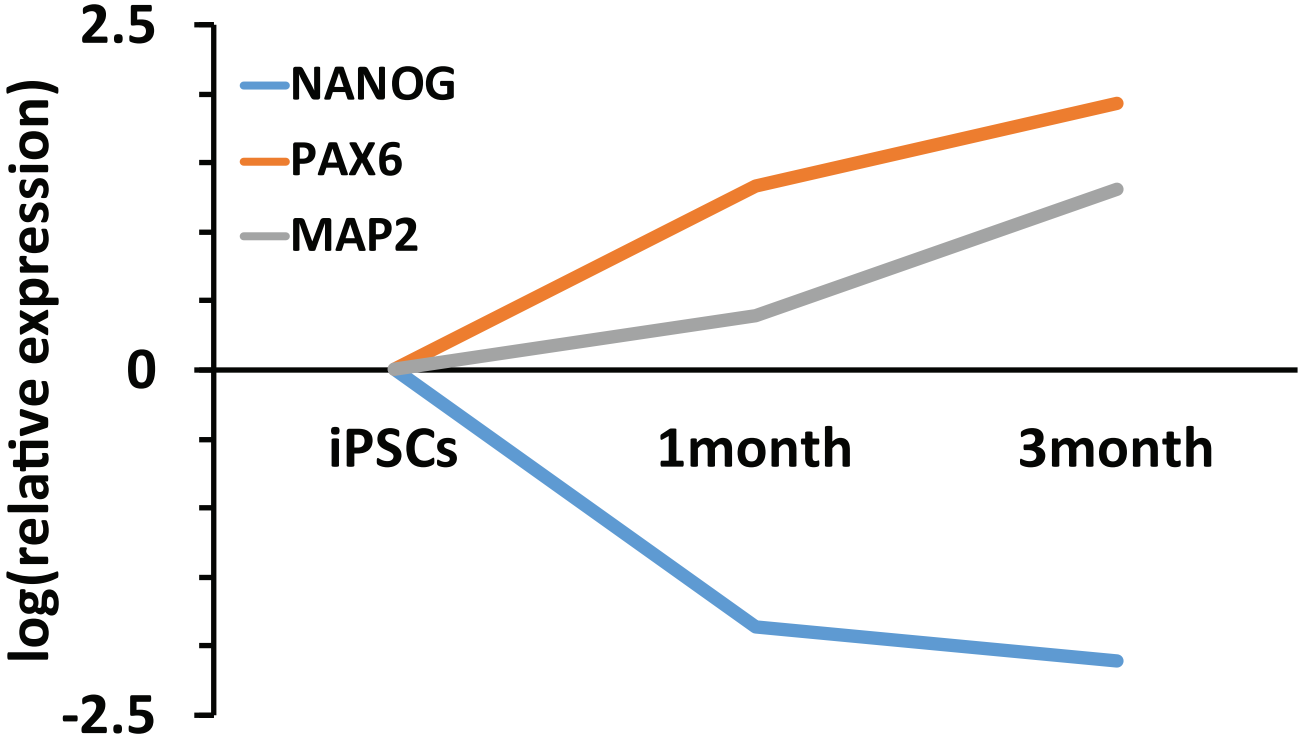

Supplementary Fig. S5

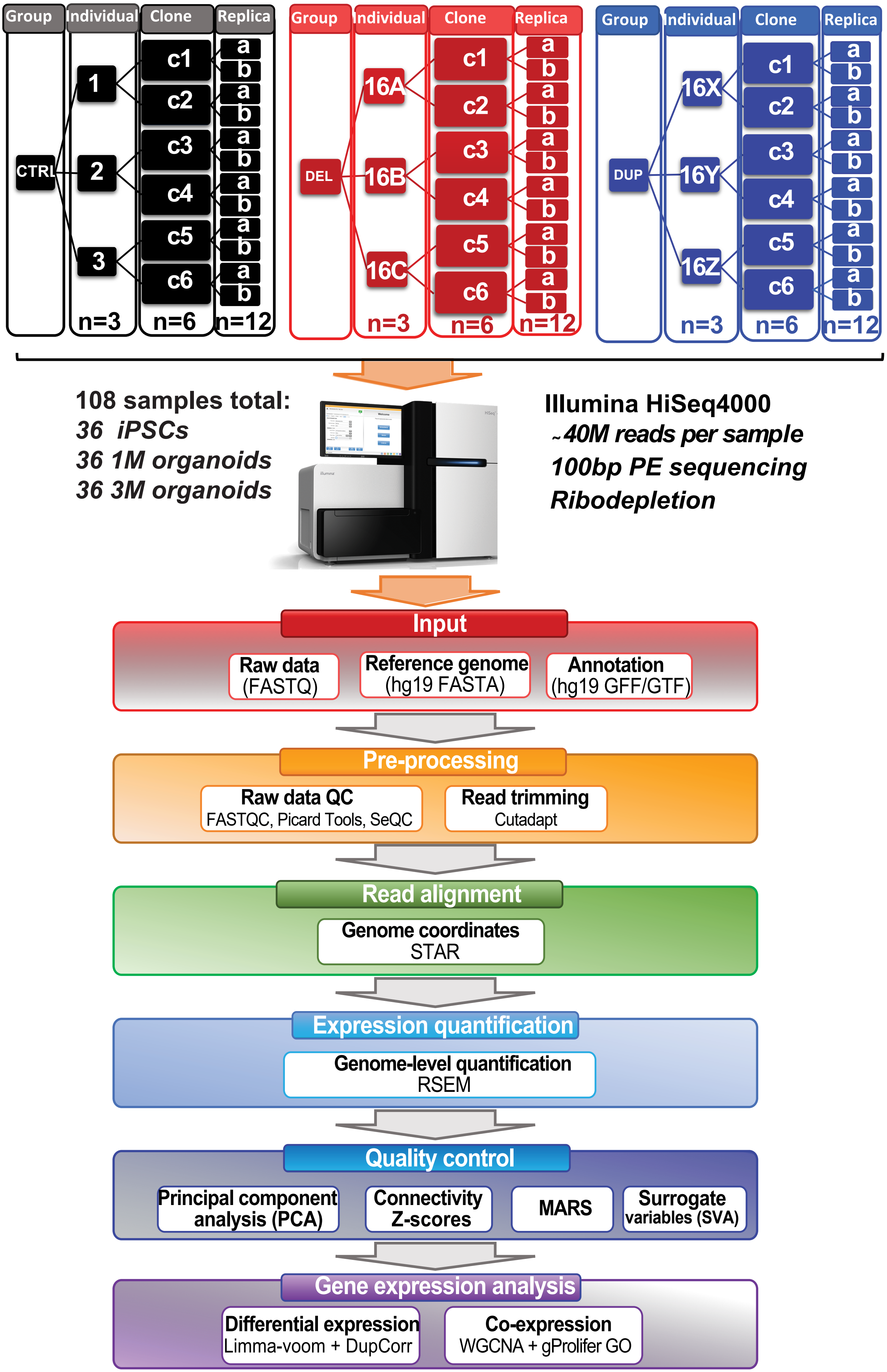

Supplementary Fig. S6

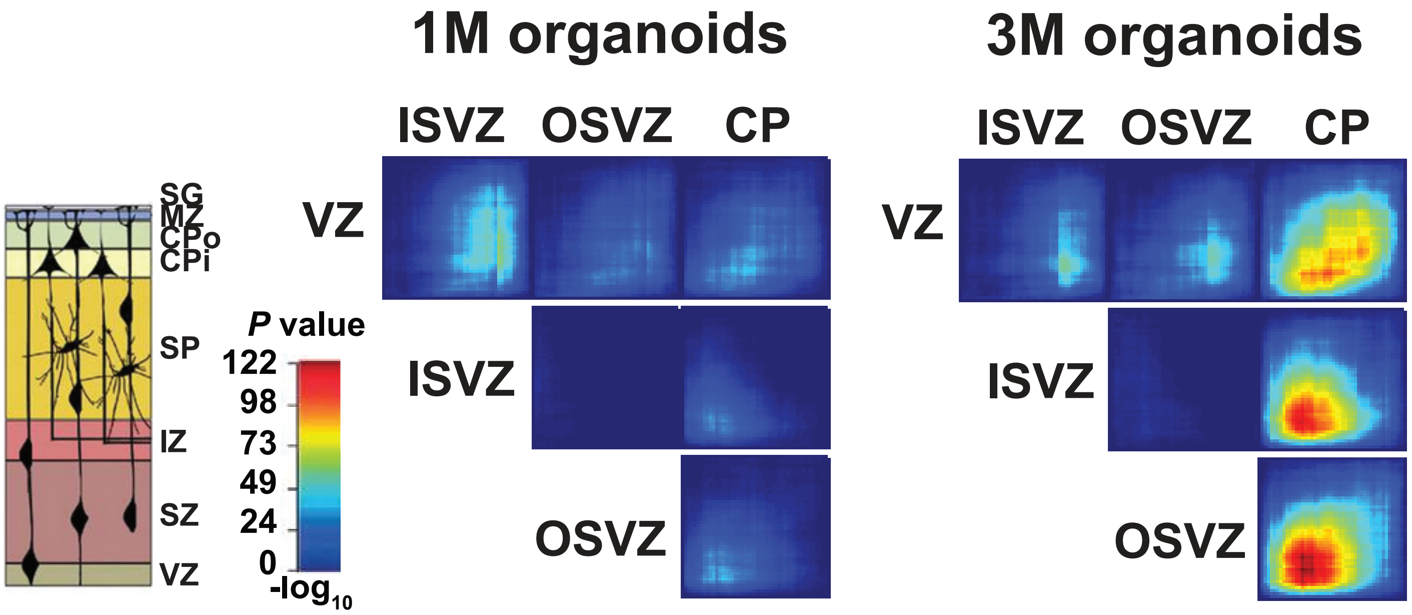

Supplementary Fig. S7

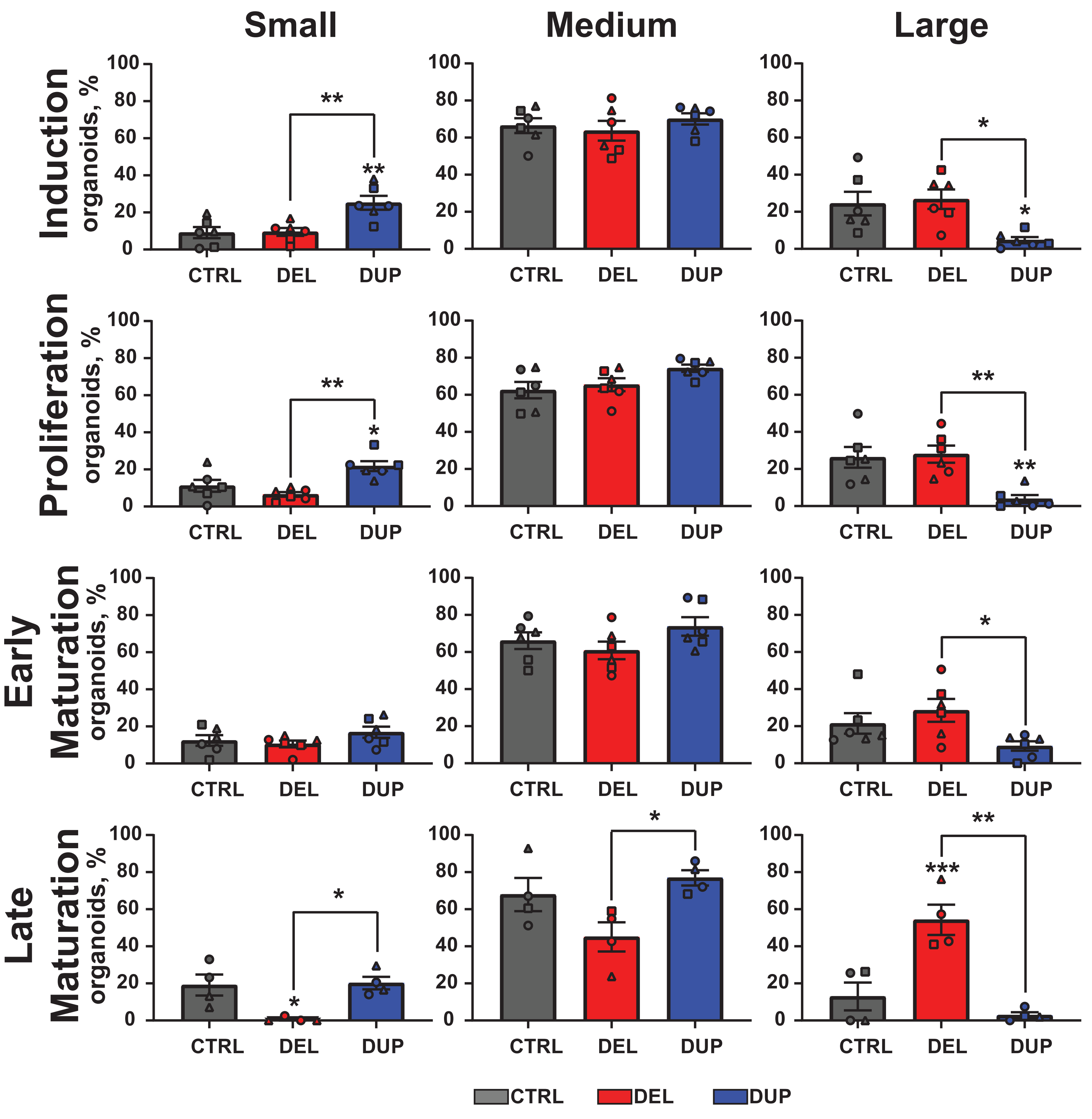

Supplementary Fig. S8

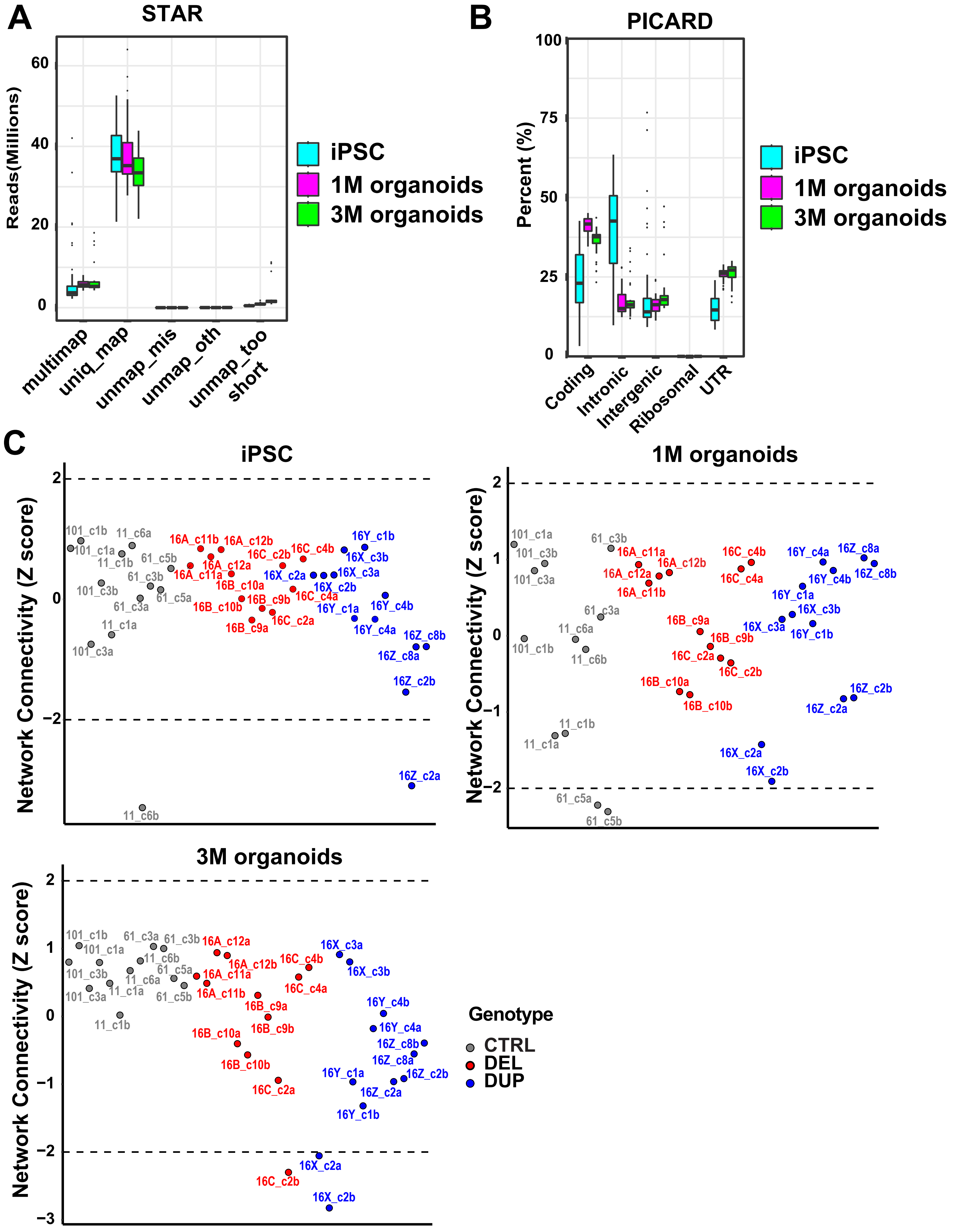

Supplementary Fig. S9

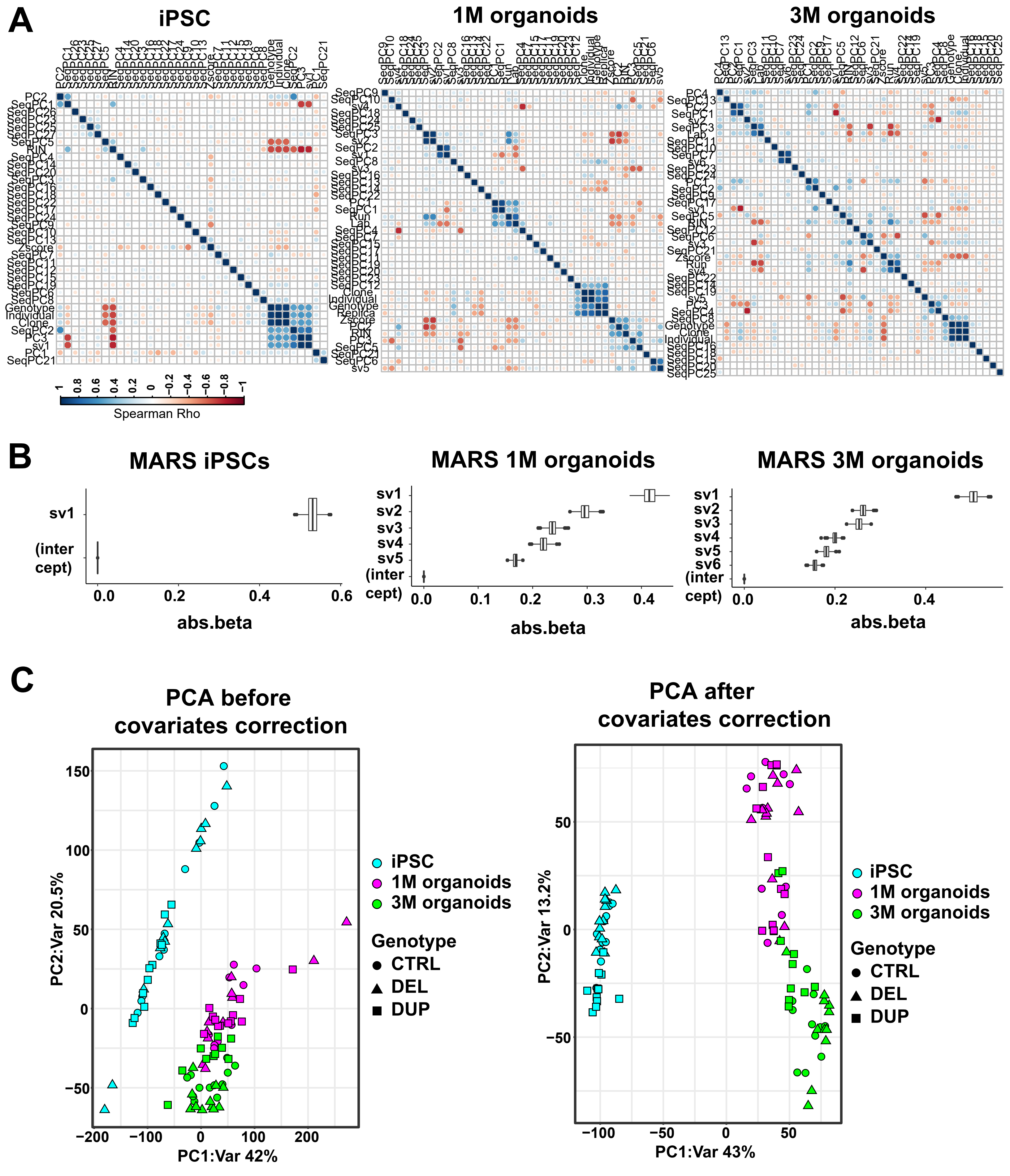

Supplementary Fig. S10

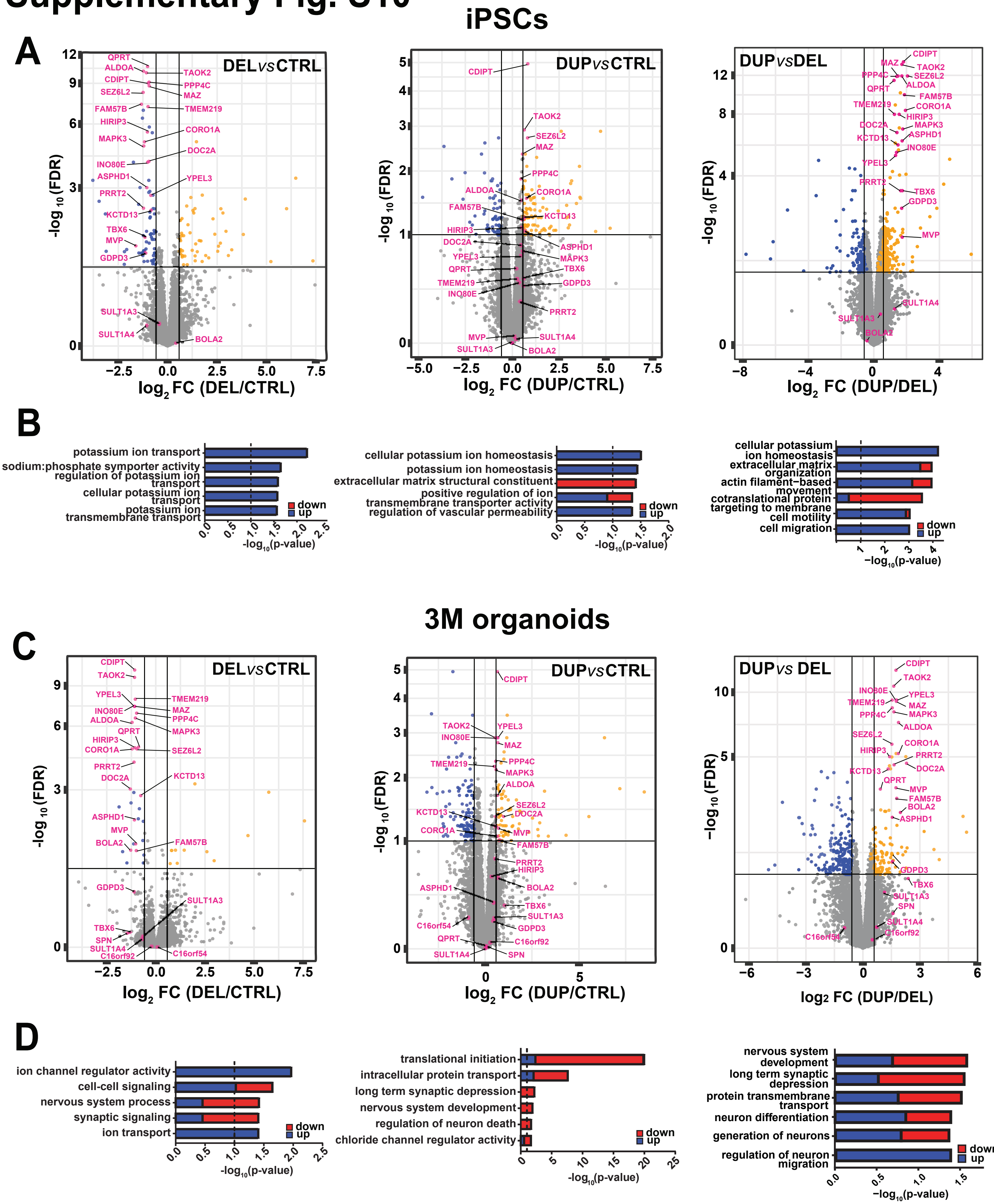

Supplementary Fig. S11

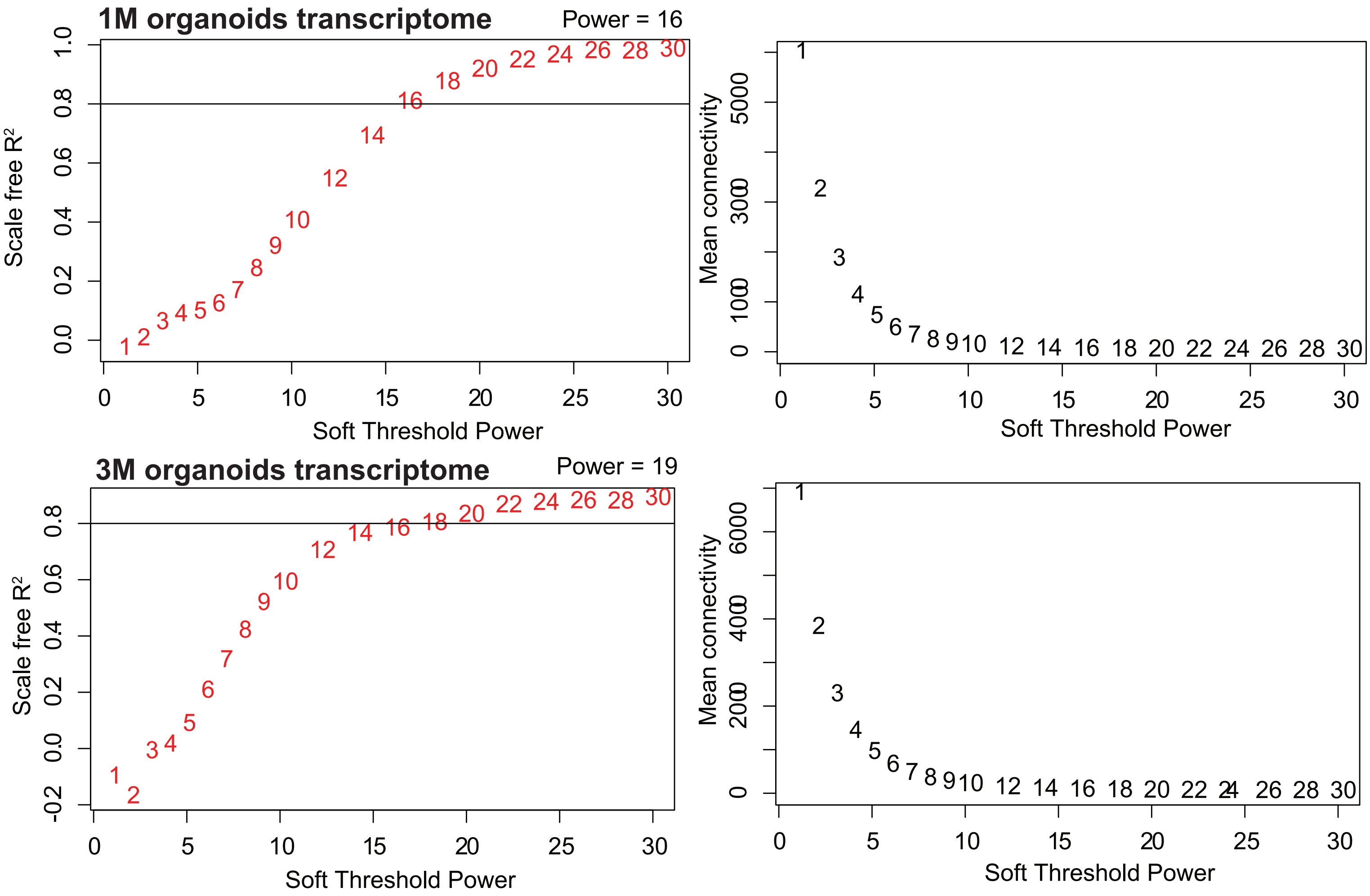

Supplementary Fig. S12

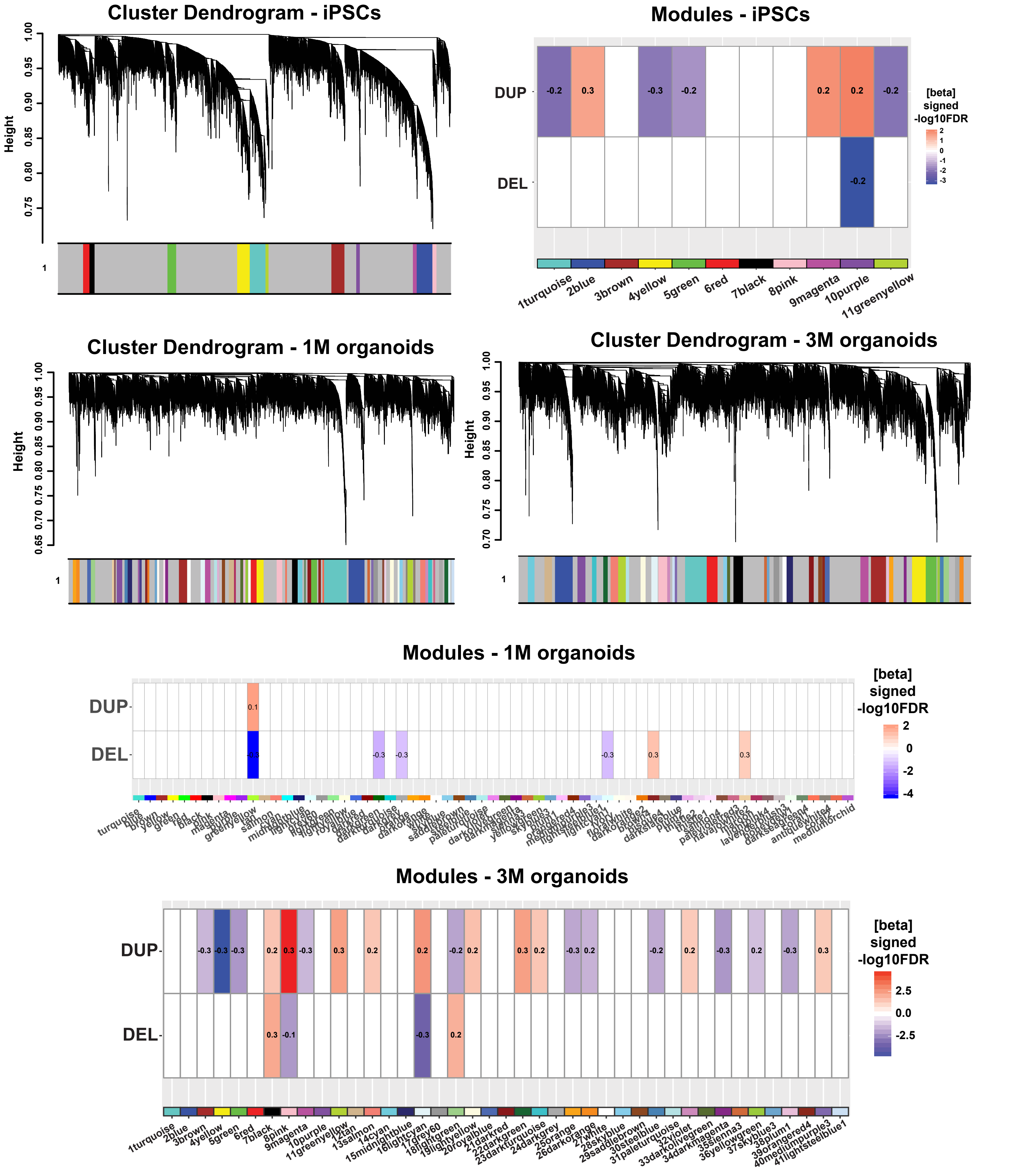

Supplementary Fig. S13

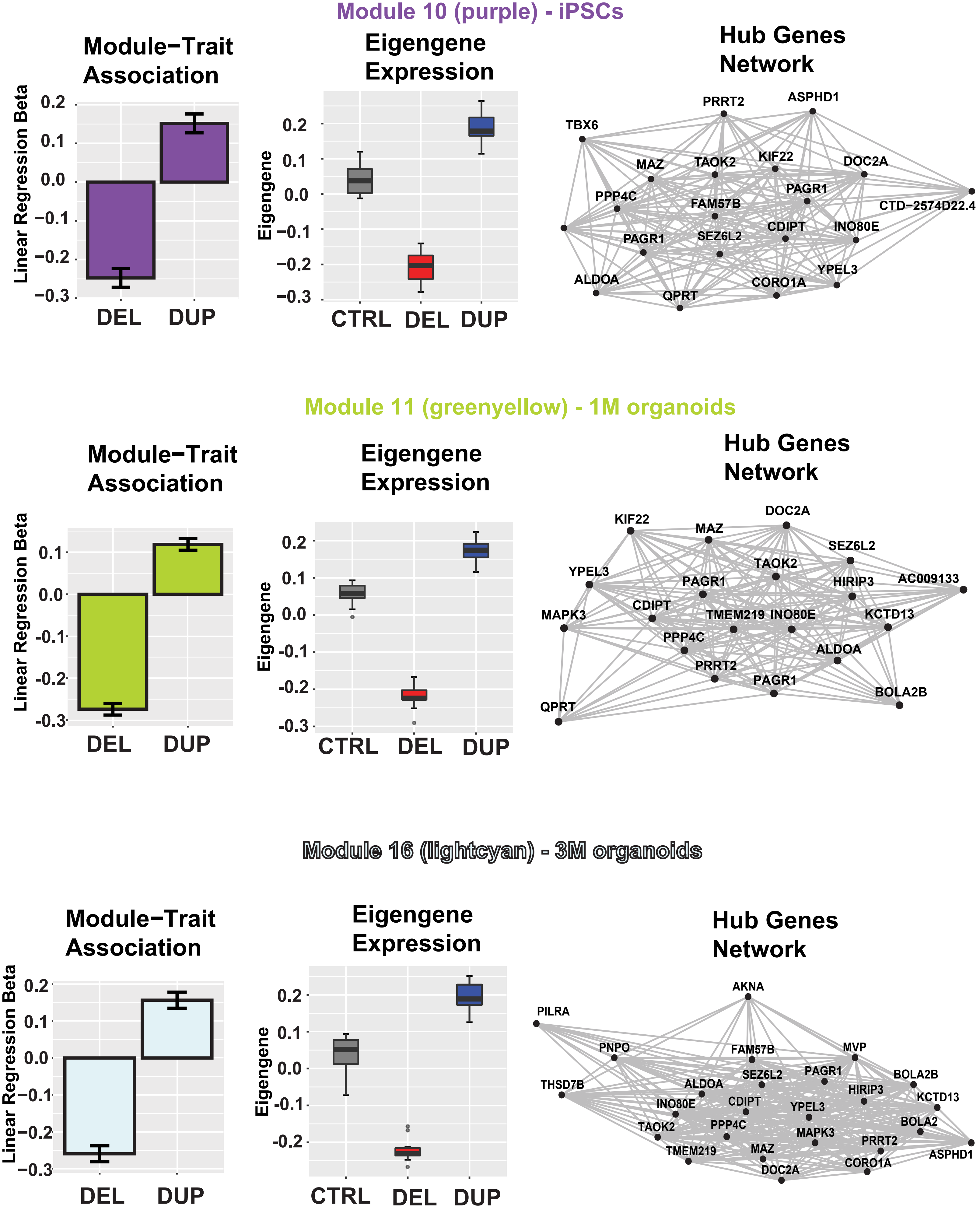

Supplementary Fig. S14

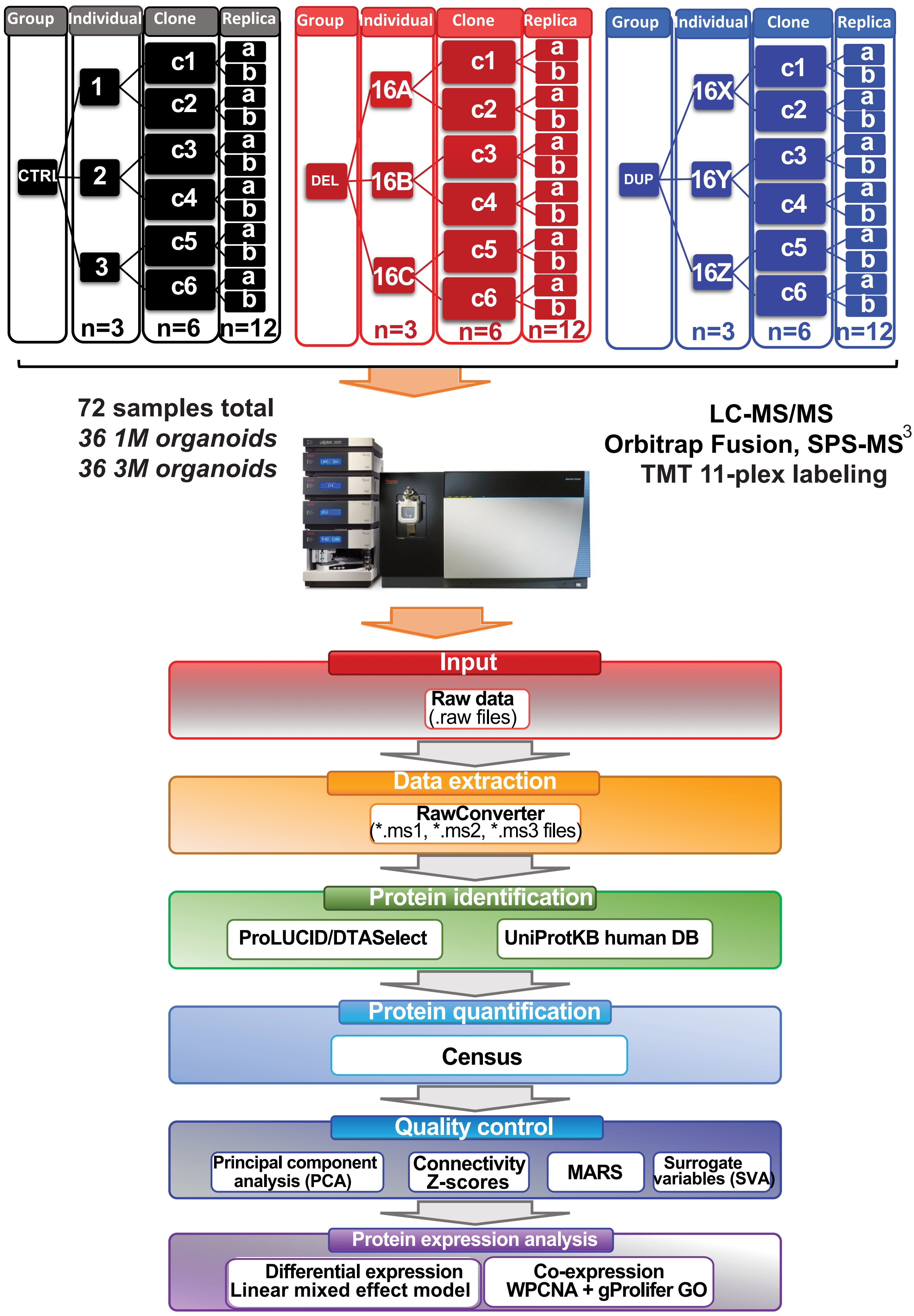

Supplementary Fig. S15

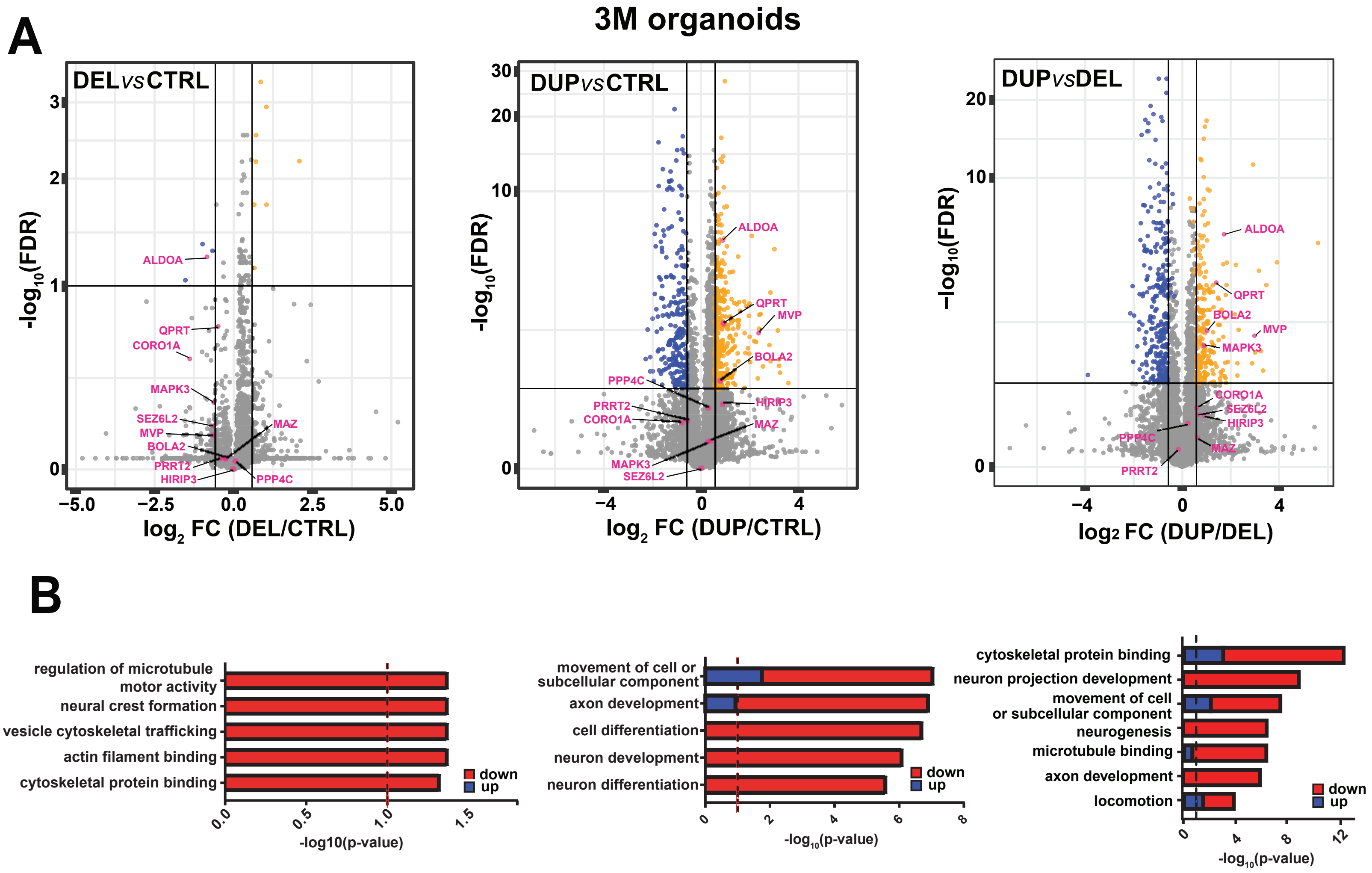

Supplementary Fig. S16

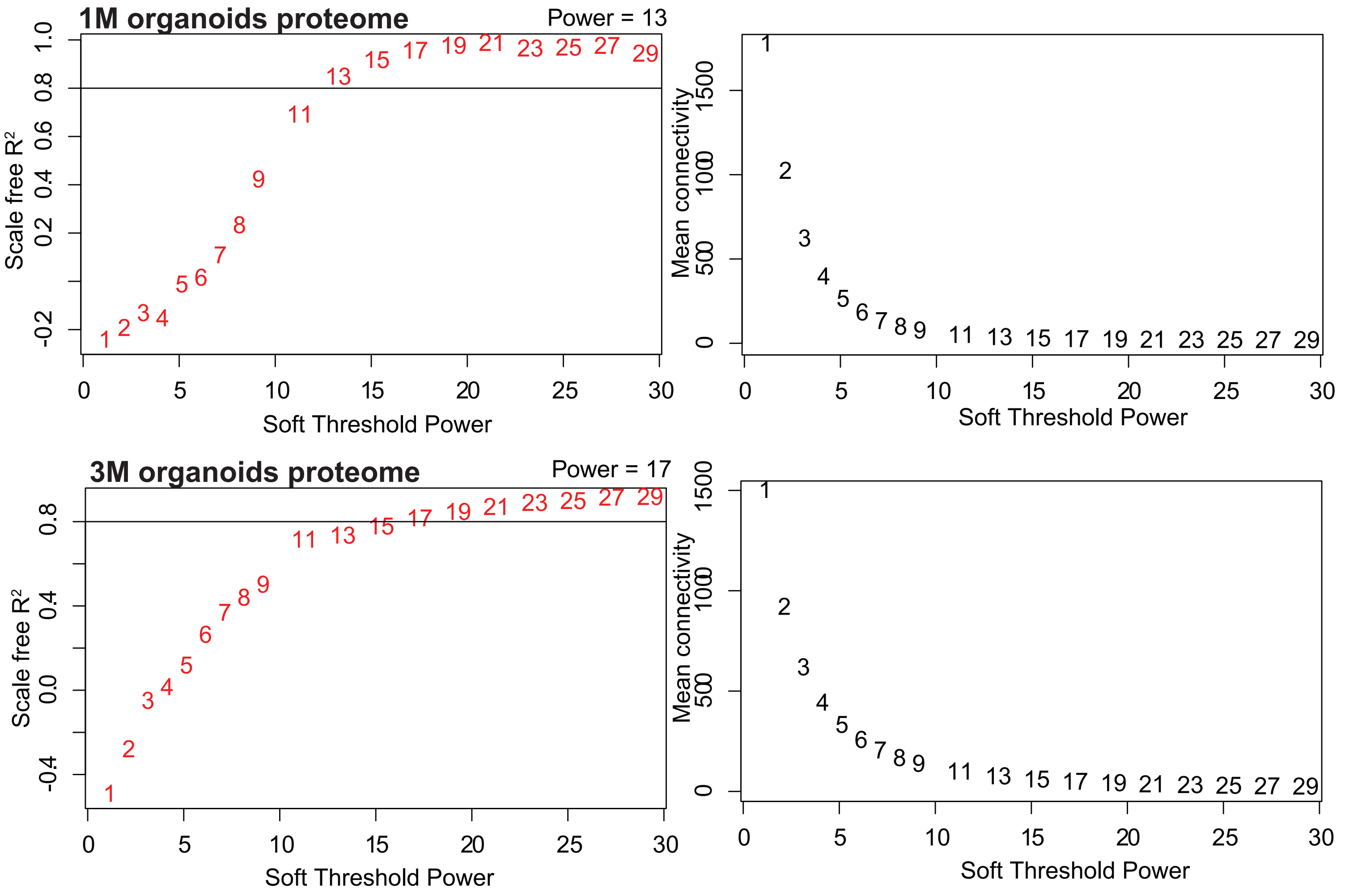

Supplementary Fig. S17

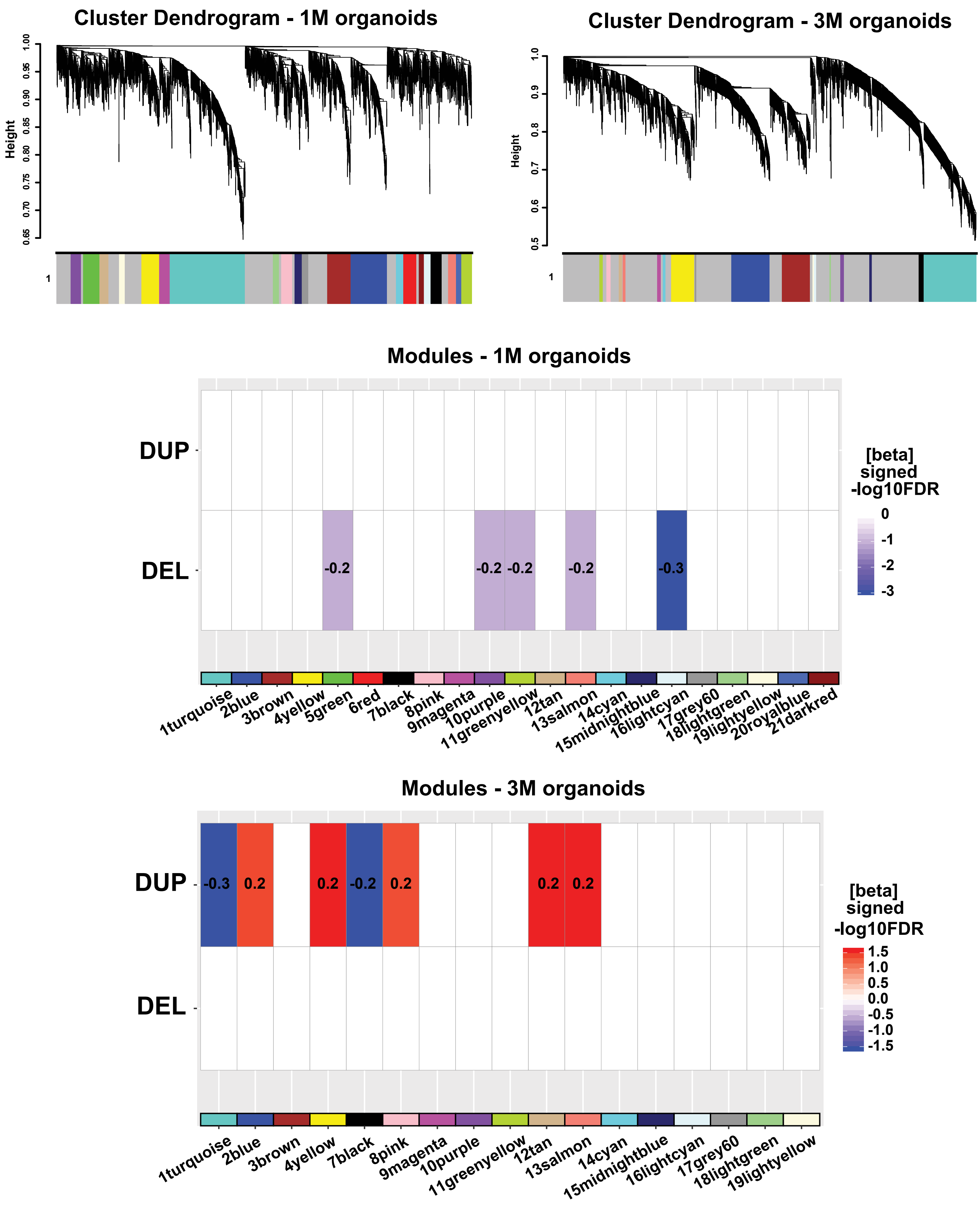

Supplementary Fig. S18

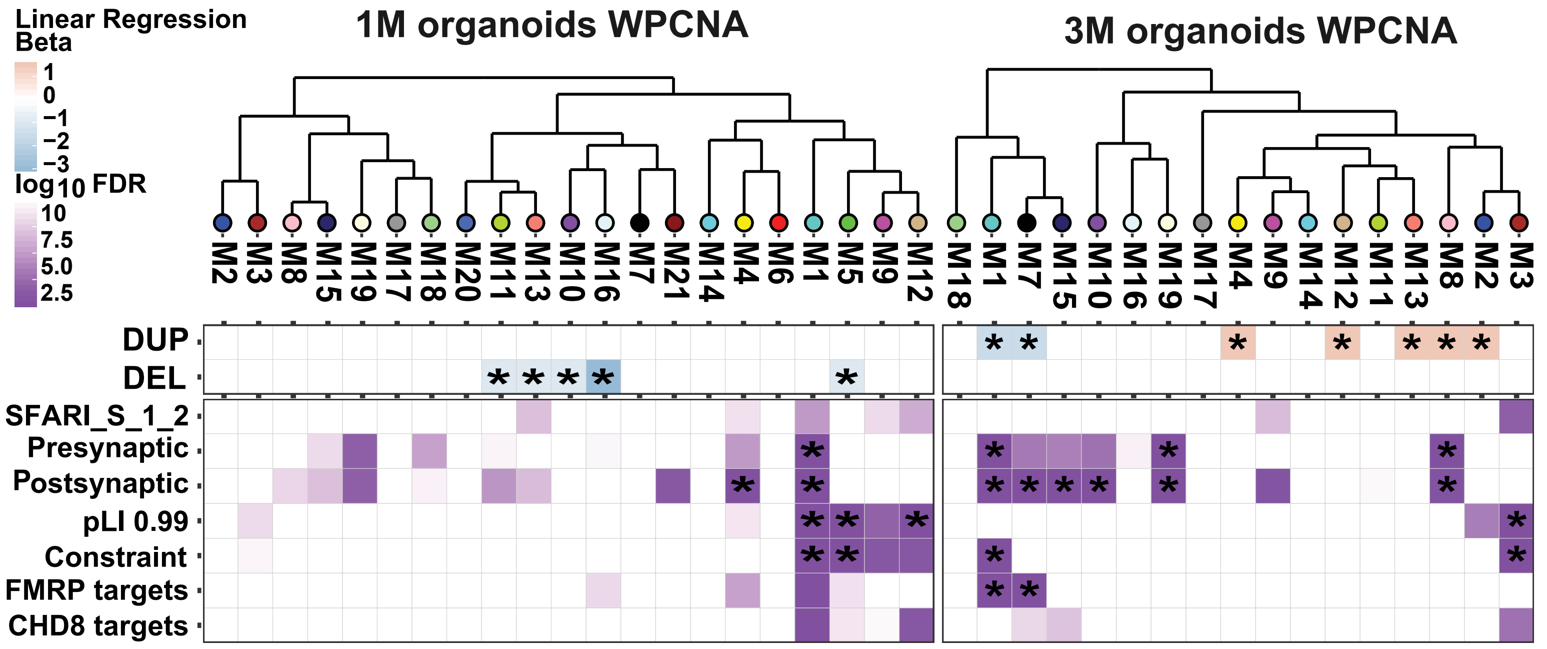

Supplementary Fig. S19

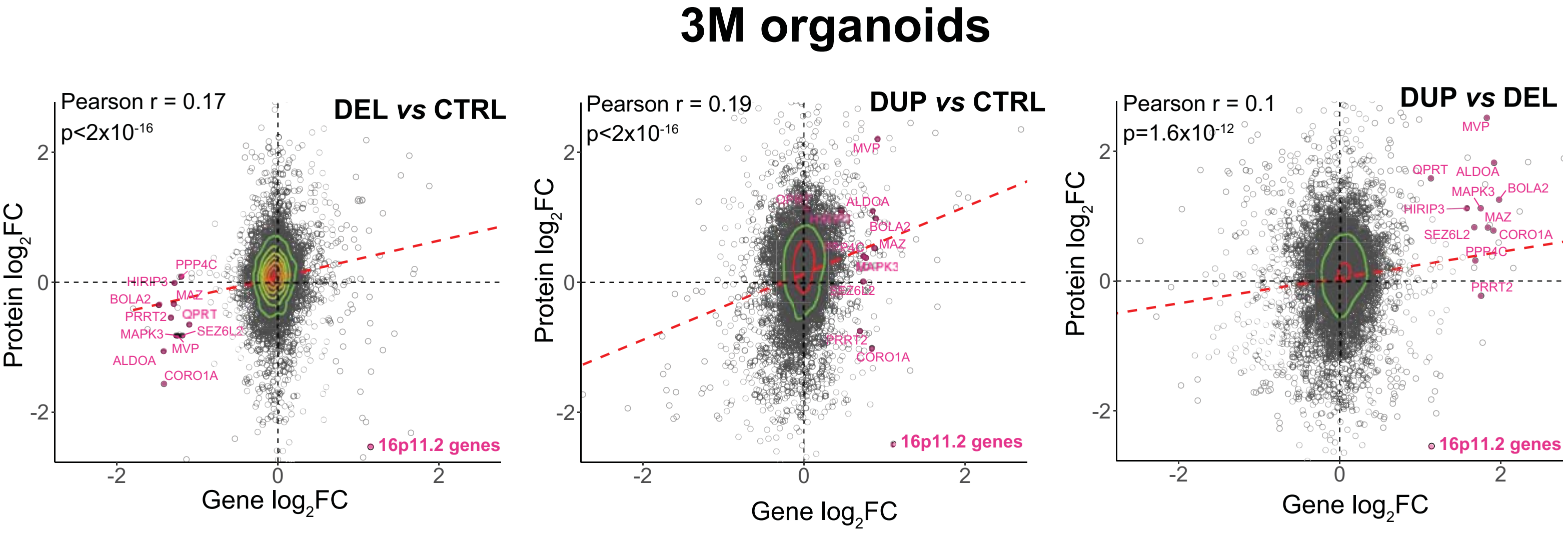

Supplementary Fig. S20

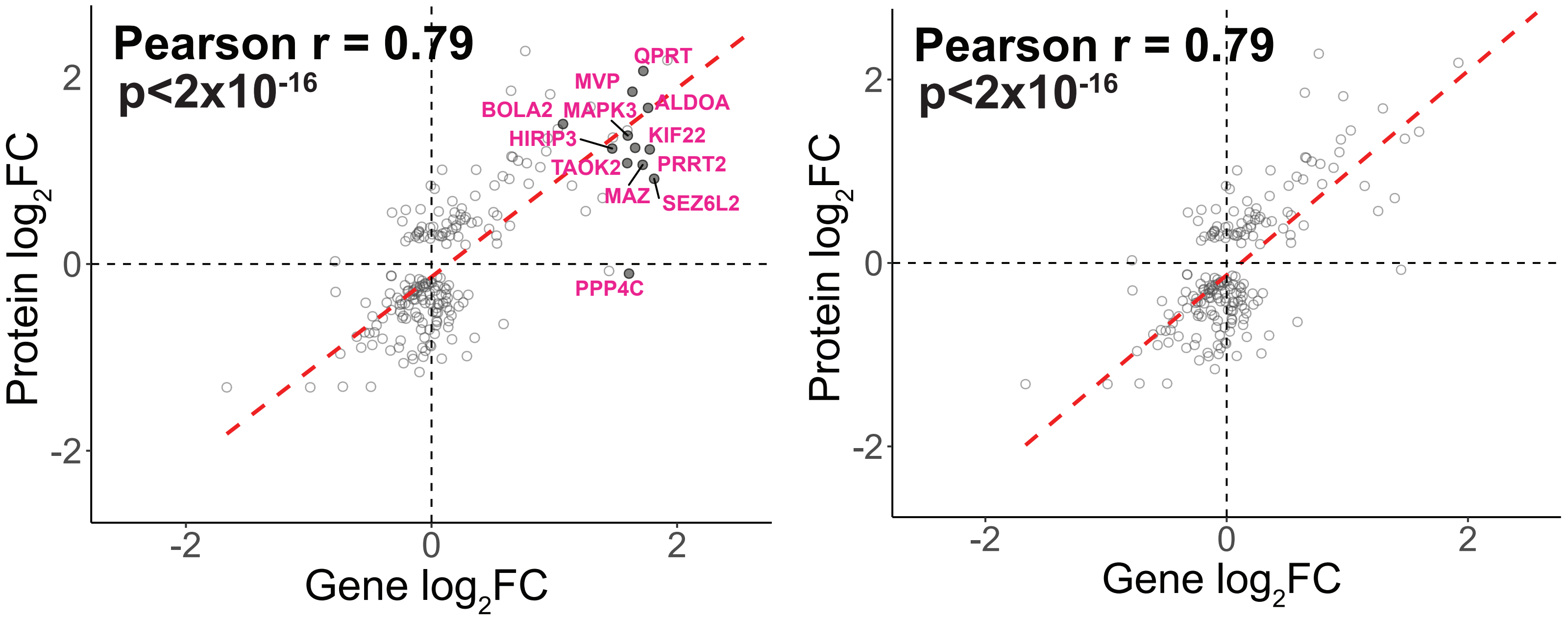

Supplementary Fig. S21

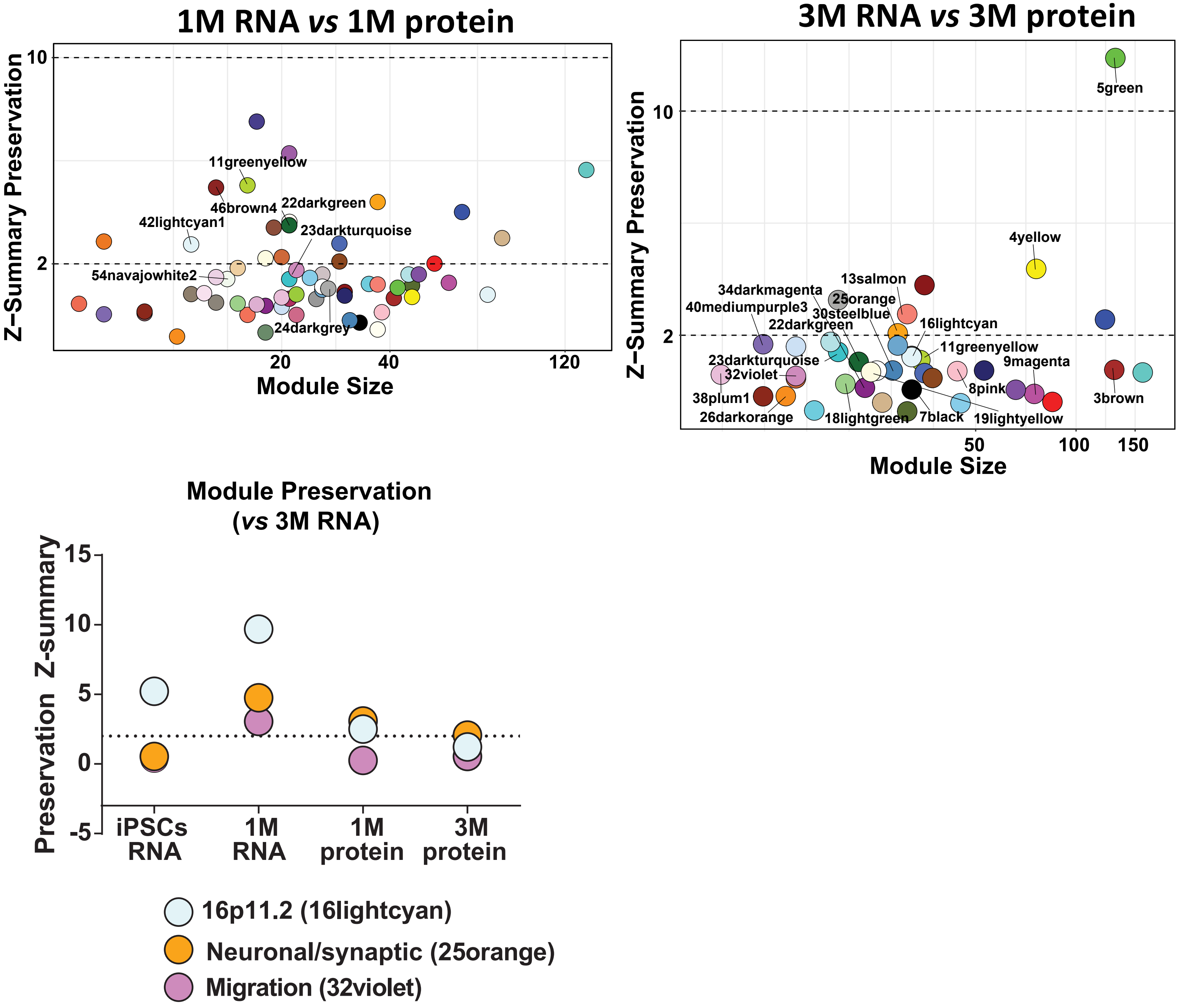

Supplementary Fig. S22

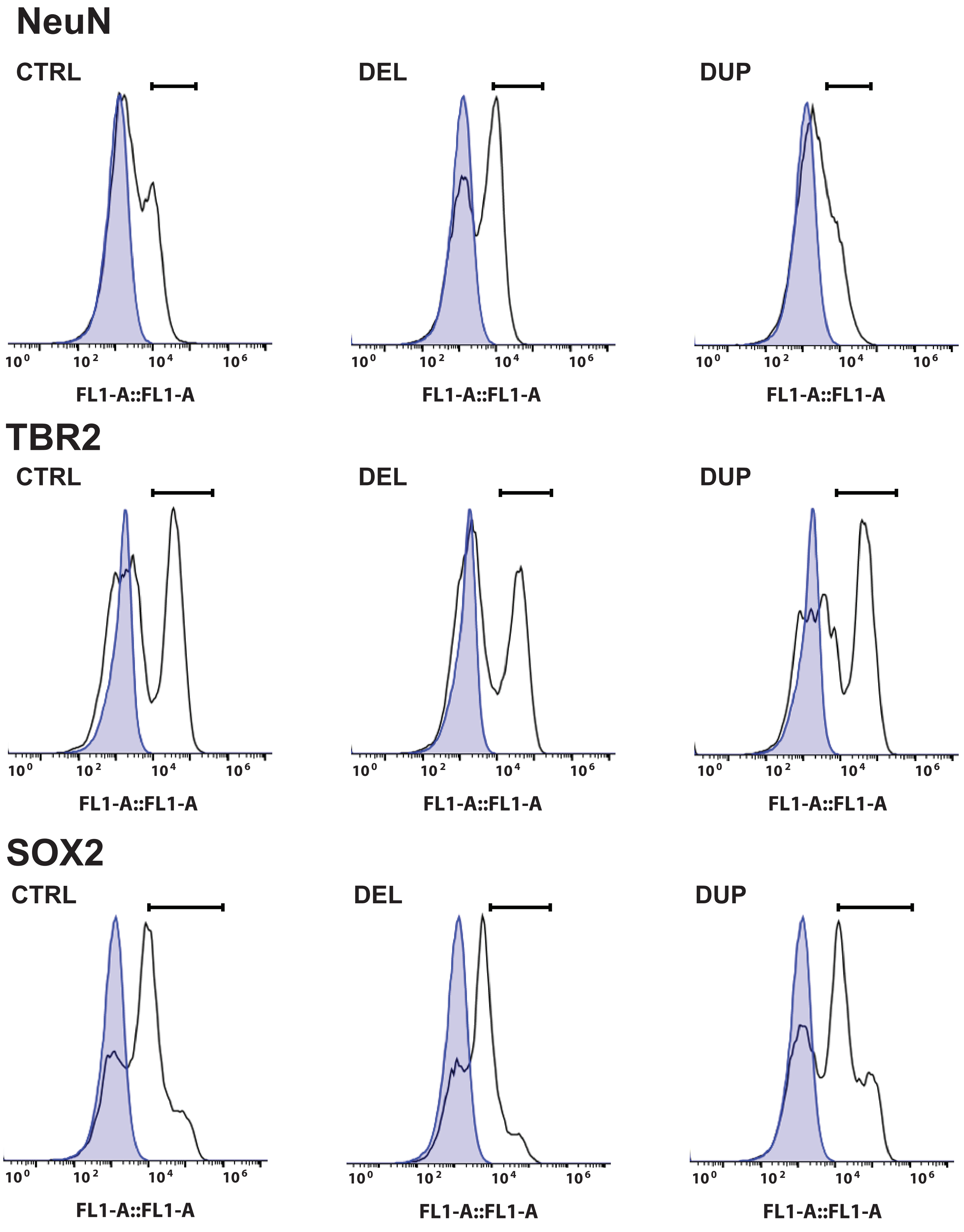

Supplementary Fig. S23

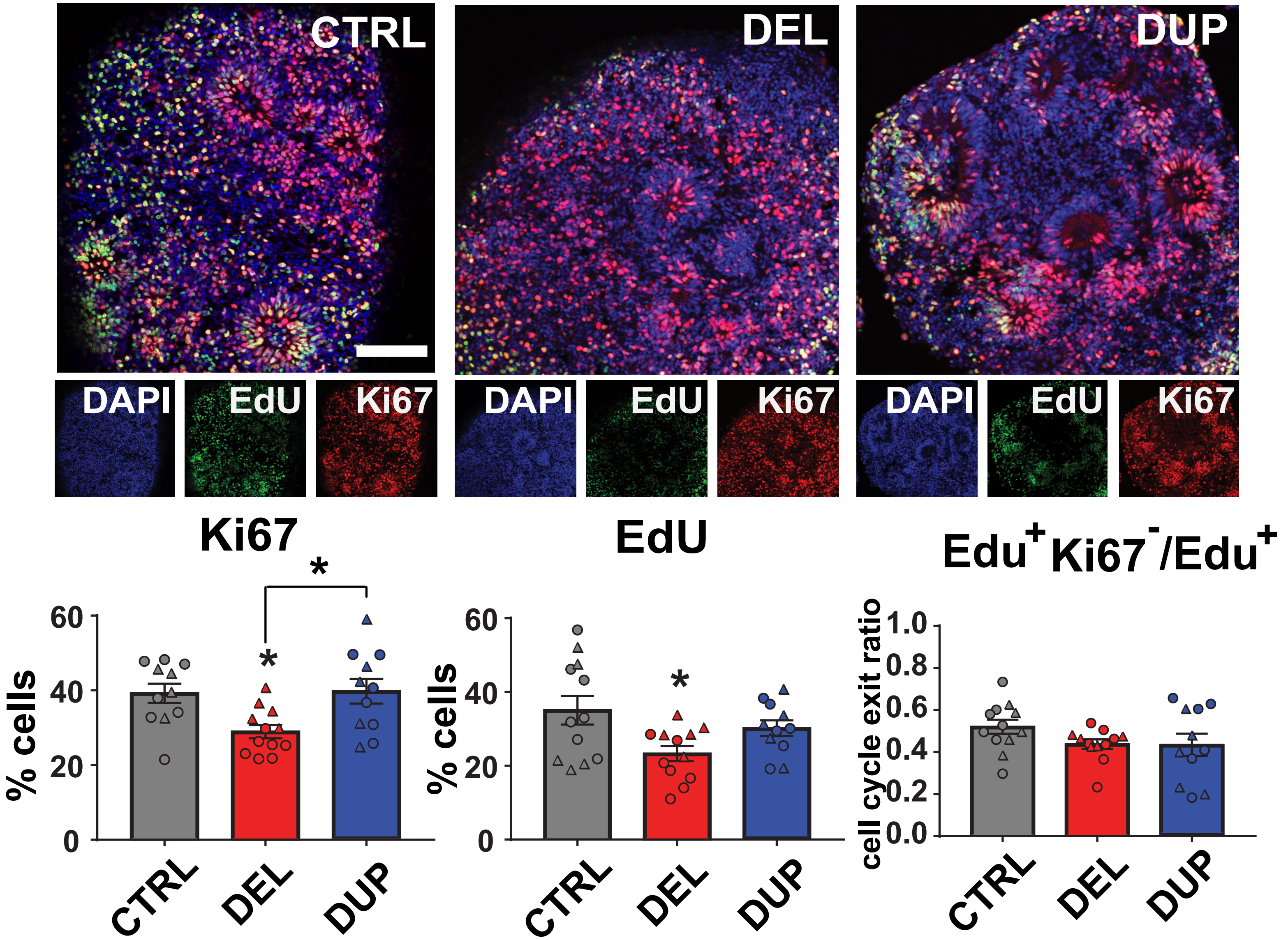

Supplementary Fig. S24

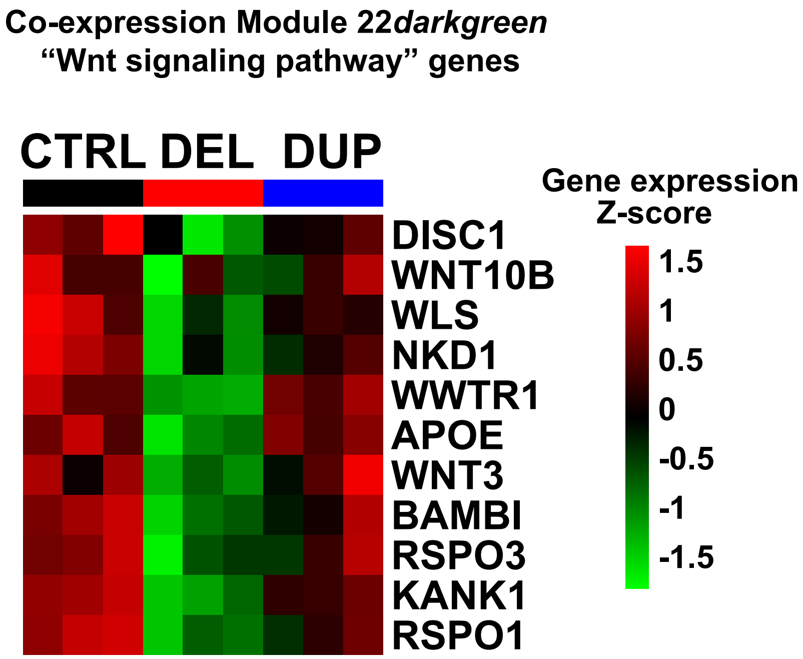

Supplementary Fig. S25

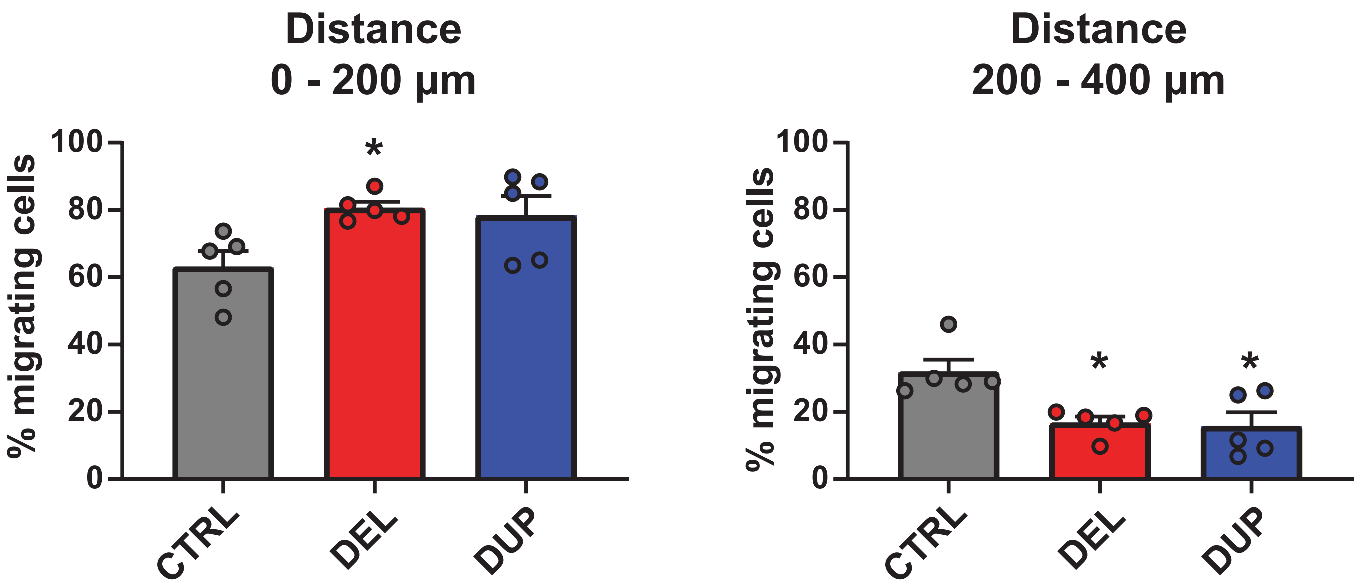

Supplementary Fig. S26

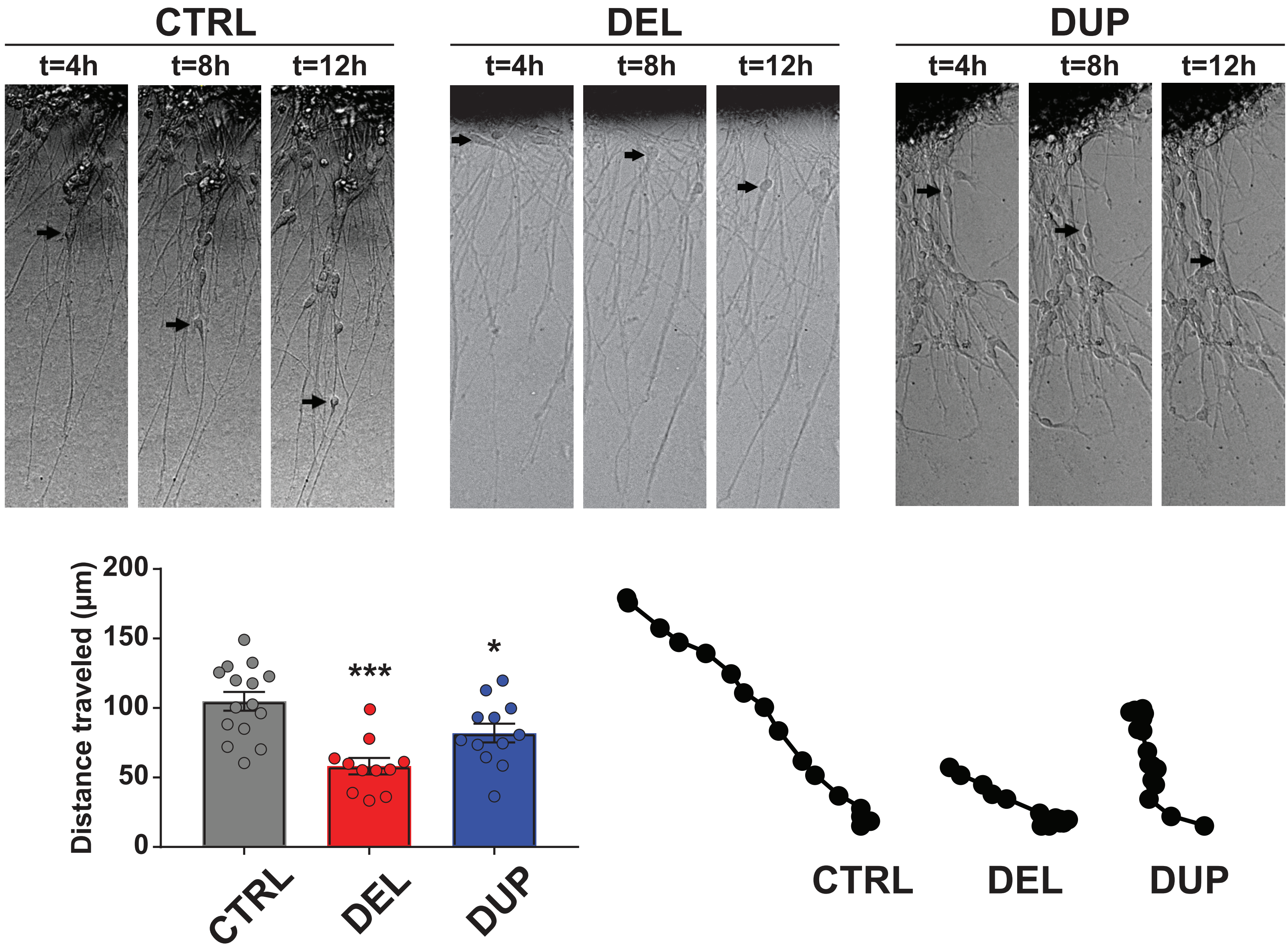

Supplementary Fig. S27

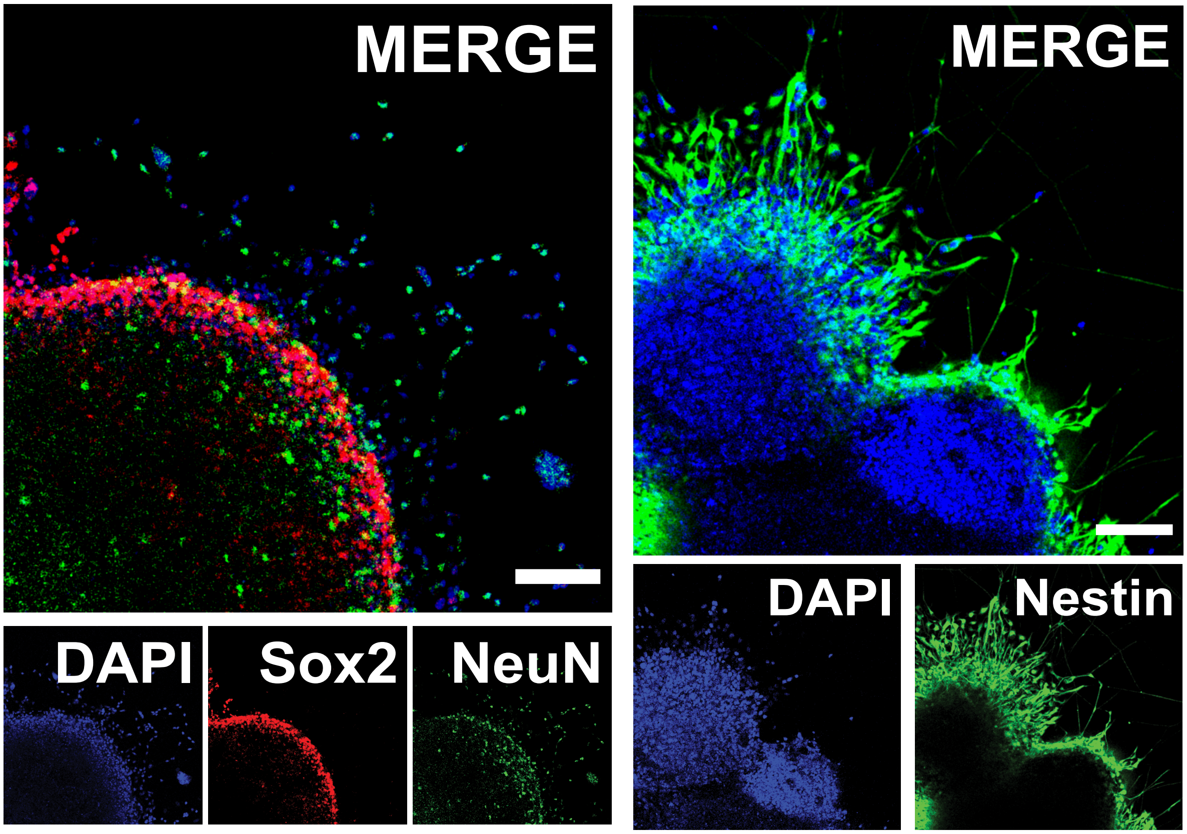

Supplementary Fig. S28

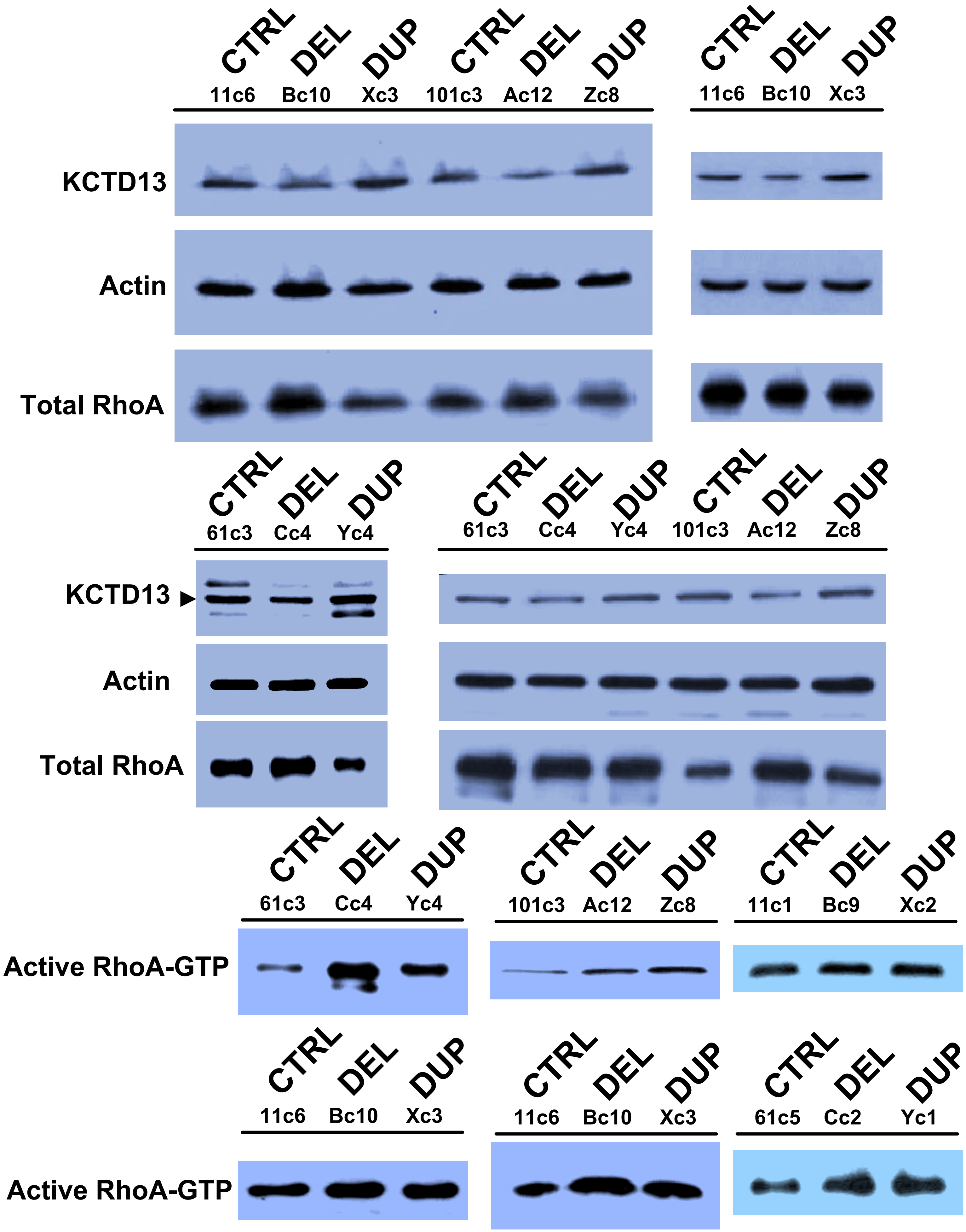

Supplementary Fig. S29

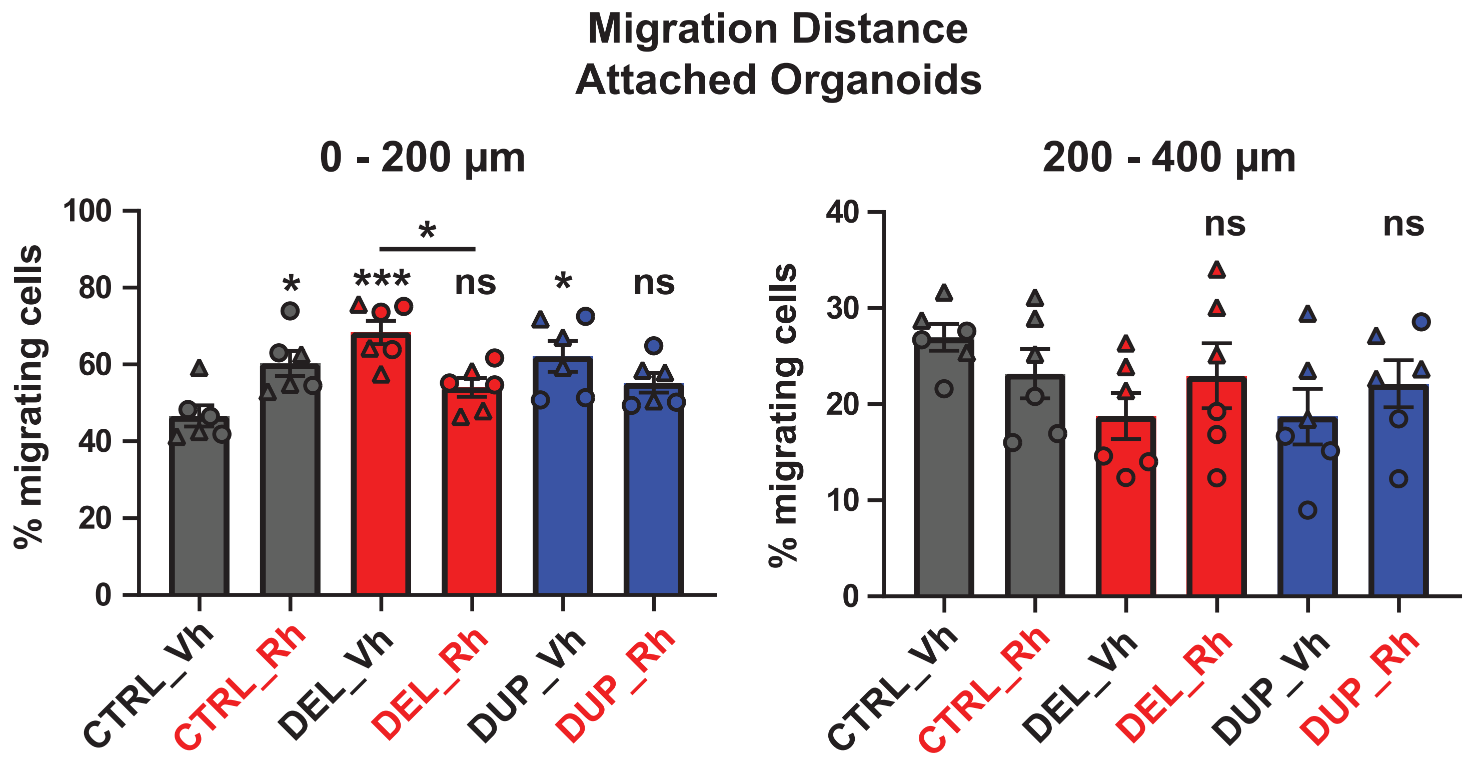

Supplementary Fig. S30

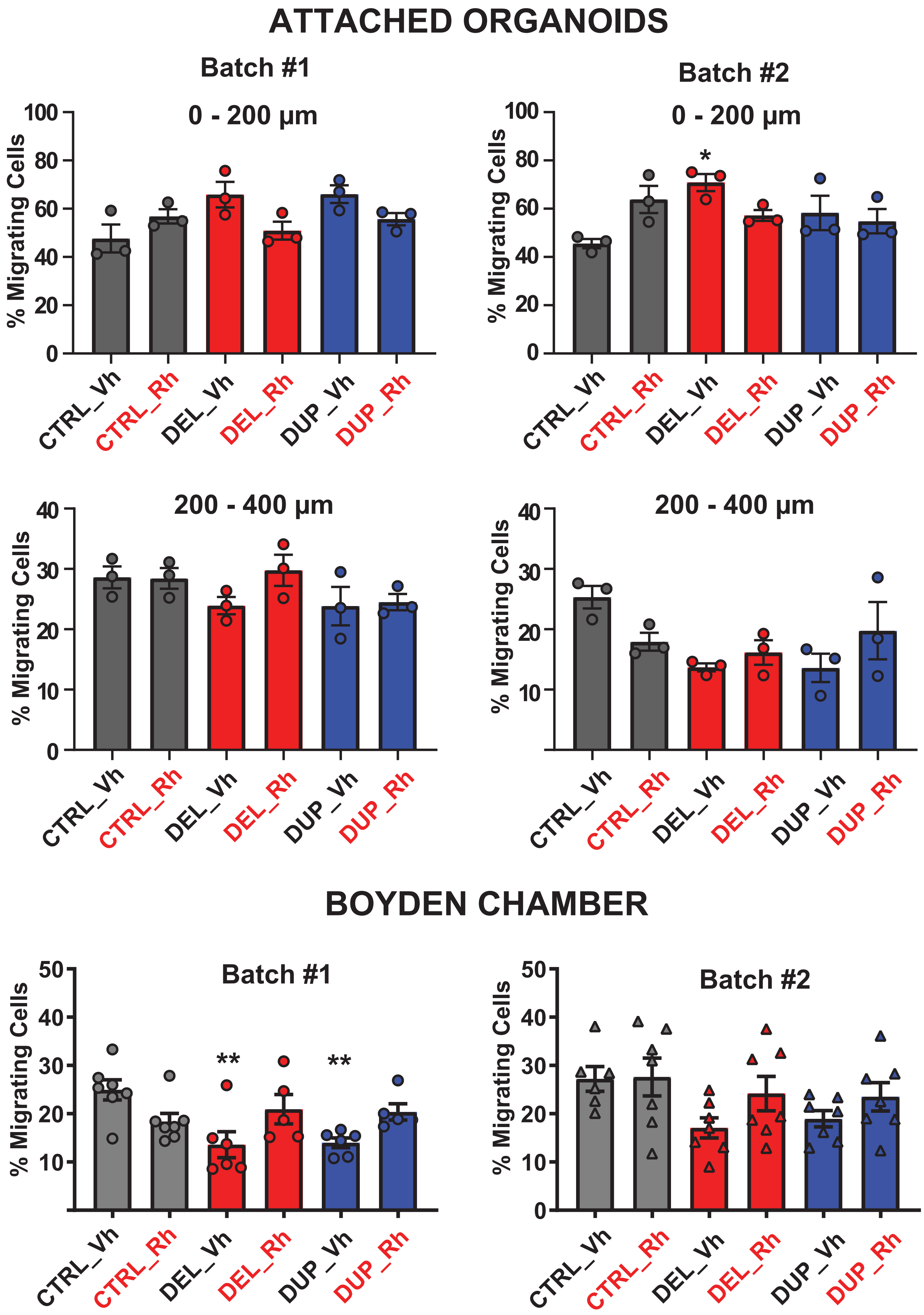

Supplementary Fig. S31

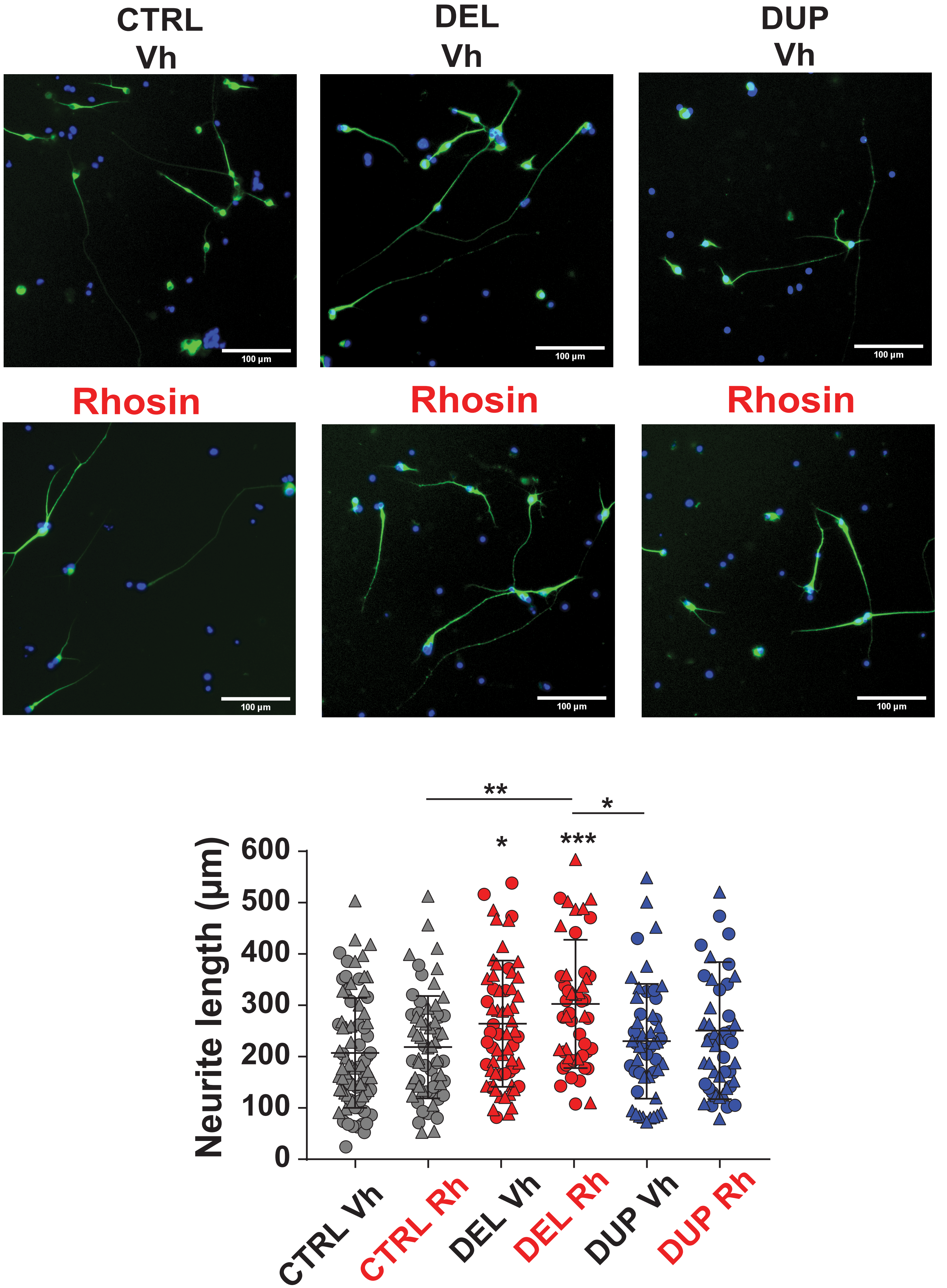

Supplement: Supplementary file 1 — Supplementary Figures [file 41380_2021_1243_MOESM1_ESM.pdf]
