# Supplementary material for: Cortical organoids model early brain development disrupted by 16p11.2 copy number variants in autism
Source: Mol Psychiatry. 2021 Aug 26;26(12):7560–80. doi: 10.1038/s41380-021-01243-6 (PMC8873019; doi:10.1038/s41380-021-01243-6)
Supplement: Supplementary file 16 — Suppl Figures and Tables legends [file 41380_2021_1243_MOESM16_ESM.docx]

Supplementary Figure legends

**Figure S1. Immunohistochemical validation of iPSC’s pluripotency.** Representative image of patient-derived iPSCs immunostained with DAPI, NANOG and Lin28 (left), or DAPI, Tra-1-60 and Oct4 (right). Scale bar: 100µm.

**Figure S2.** **Transcriptional validation of iPSCs pluripotency**. Six pluripotency markers were quantified in patient-derived fibroblasts and corresponding iPSCs by RT-qPCR. Graph shows the average of three biological replicates for each cell line. 16pA, 16pB and 16pC are lines derived from the 16p11.2 DEL patients; and 16pX, 16Y and 16pZ are lines derived from 16p11.2 DUP patients.

**Figure S3.** **CNV burden analysis of patient-derived iPSC clones**. Microarray genotyping of fibroblasts and iPSCs, and CNV burden analyses using PennCNV {Wang, 2007 #6951} is shown. The presence of 16p11.2 CNV in DELs and DUPs was confirmed in all fibroblast and iPSC clones, and 16p11.2 CNV was removed in subsequent burden analyses. Patients’ mean CNV burden was defined as the CNV burden in fibroblasts, and standard deviation as the burden in all iPSC clones from the same patient. CNV burden between fibroblasts and iPSC clones for each patient was compared. Only iPSC clones with a CNV burden score <1 SD *vs* patients fibroblasts are shown. Fibroblasts CNV burden score is shown for reference. Arrows point to the iPSC clones selected for cortical organoids production.

**Figure S4. Time course of the cell type markers.** Transcriptional analysis of three cell type markers (NANOG, PAX6 and MAP2) in 16p11.2 patient-derived iPSCs, 1M and 3M cortical organoids by RT-qPCR. Graph shows the average of all patient cell lines (*n*=6), with three biological replicates per patient.

**Figure S5. RNA-seq experimental design and data analysis workflow.** A total of 108 transcriptomes have been sequenced in this study. A rigorous quality control including principal component analyses, sample connectivity analyses, surrogate variable analyses and multivariate adaptive regression spline (MARS) for covariate selection has been performed. The Limma-voom model with duplicate correlation function has been applied to account for clone replicates derived from the same patient in order to avoid pseudo-replication in the differential gene expression analyses {Germain, 2017 #7057}.

**Figure S6. Predicted laminar transitions for 1M and 3M organoids compared to fetal neocortex**. The Figure was produced using TMAP {Stein, 2014 #6280} and the transcriptome of germinal zones of six 13–16 PCW human fetal neocortices {Fietz, 2012 #7009}. Rank-rank hypergeometric overlap (RRHO) maps for CTRL organoids (n=12 datasets) from 3 patients, 2 clones per patient, 2 replicates per clone are shown, with CTRL iPSCs (n=12 datasets) used as a second time point. Each pixel represents the overlap between fetal brain and organoids transcriptome, color-coded according to the -log_10_ p-value of a hypergeometric test. On each map, the extent of shared upregulated genes is displayed in the bottom left corner, whereas shared downregulated genes are displayed in the top right corners. Ventricular zone (VZ), Inner Subventricular zone (ISVZ), Outer Subventricular zone (OSVZ) and Cortical Plate (CP) are shown.

**Figure S7. Statistical analyses of organoid size measurements across time course of differentiation and maturation.** Individual bins for each size are plotted. The symbols represent individual batches (consisting of one replica from one clone from one patient or control), p-values were calculated using one-way ANOVA with Tukey’s multiple comparison, ***p<0.001, **p<0.01, *p<0.05. Stars on top of the bars represent comparison against CTRL. For size comparison, the “large” group was defined as a proportion of organoids with size higher than 1 Standard Deviation (SD) within the batch; the “small” group as a proportion of organoids with size lower than 1 SD within the batch, and the “medium” group comprised the remaining organoids. The proportions for each batch were averaged for final quantification. Statistical analyses details are shown in **Table S3**.

**Figure S8. Initial quality control metrics for RNA-seq data. (A)** Sequencing metrics from STAR (2.5.3a) for each group of samples (iPSCs, 1M and 3M old organoids). **(B)** Sequencing metrics from PicardTools (v2.12) for each group of samples (iPSC, 1M and 3M organoids). **(C)**  Sample outlier removal performed with WGCNA package in R for each group of samples (iPSCs, 1M and 3M old organoids) based on Z-scores of standardized network connectivity. Outliers were defined as samples with Z scores of <(-2).

**Figure S9. Quality control metrics for RNA-seq data. (A)** Correlation plots among the top seqPCs (PCs that summarize the RNA-Seq QC metrics), surrogate variables selected by MARS, and other potential covariates (Run, RIN, Individual, Lab, Clone, Replica, Genotype, Z score) for each group of samples (iPSCs, 1M and 3M old organoids) generated by corrplot package in R. The spearman correlation coefficients values correspond to the areas of the circles. The legend shows the spearman correlation coefficient ρ values. **(B)** Covariates for each group of samples (iPSCs, 1M and 3M old organoids) selected by MARS (implemented in earth package in R). **(C)** First two principal components (PCs) of gene expression values, calculated using “prcomp” function in R, are shown before (left panel) and after (right panel) covariate correction for the iPSC, 1M and 3M organoids. Colors represent samples (iPSCs, 1M and 3M organoids), and symbols represent genotypes: CTRL (circles), DEL (triangles) and DUP (squares).

**Figure S10. Differential gene expression and Gene Ontology annotations for iPSCs and 3M cortical organoids.** Volcano plots of differentially expressed genes in DEL *vs* CTRL (left), DUP *vs* CTRL (middle), and DUP *vs* DEL (right) for iPSCs (A) and 3M organoids (C). Genes within 16p11.2 CNV are colored in pink. Genes colored in orange are upregulated; genes colored in blue are downregulated. (B) GO terms for differentially expressed genes in iPSCs for DEL *vs* CTRL (left), DUP *vs* CTRL (middle), and DUP *vs* DEL (right). (D) GO terms for differentially expressed genes in 3M organoids for DEL *vs* CTRL (left), DUP *vs* CTRL (middle), and DUP *vs* DEL (right).

**Figure S11. Scale-free topology of gene co-expression networks for organoids.** The soft threshold power of 16 for 1M organoids and 19 for 3M organoids was chosen to correspond to the scale-free topology fit index of 0.8 or higher.

**Figure S12. Gene co-expression modules for iPSCs, 1M and 3M organoids.** WGCNA cluster dendrograms and module eigengene-genotype association are shown. The data from all samples (DEL, DUP and CTRL) were used for module detection. Comparisons were made between each genotype (DEL or DUP) and control (CTRL) for each module enrichment to calculate the significance. Rows are genotypes (relative to CTRL) and columns are modules. Number in each tile is a beta value from linear mixed effect model, and color of each tile indicates statistical significance (see **Materials and Methods**). A total of 7 modules were significantly associated with DEL or DUP genotypes in iPSCs, total of 6 modules in 1M old organoids, and a total of 21 modules in 3M old organoids.

**Figure S13. 16p11.2 gene modules in iPSCs, 1M and 3M old organoids.** The significantly associated with genotype 16p11.2 module was detected in all datasets (*10purple* in iPSCs, *11greenyellow* in 1M organoids, and *16lightcyan* in 3M old organoids). Left column of each panel: module-trait association. Middle column of each panel: module eigengene expression for DEL, DUP and CTRL datasets. Eigengene was quantified using the data from 12 data points (n=12) corresponding to replicates from 3 patients (CTRL, DEL or DUP), 2 clones per patient and 2 replicates per clone. Some replicates were removed before the analyses during outlier detection procedure (see **Materials and Methods**). Right column of each panel: top 20 hub genes (based on kME) from each module are shown. Edges represent co-expression.

**Figure S14. Proteomics experimental design and data analysis workflow.** A total of 72 proteomes have been processed in this study by LC-MS/MS with TMT 11-plex labeling. Protein Quantification was carried out by Census. A rigorous quality control including principal component analyses, sample connectivity analyses, surrogate variable analyses and multivariate adaptive regression spline (MARS) for covariate selection has been performed. Linear mixed effect model (LME) was implemented for differential protein expression analyses.

**Figure S15. Differential protein expression and Gene Ontology annotations for 1M and 3M cortical organoids. (A)** Volcano plots of differentially expressed proteins in DEL *vs* CTRL (left), DUP *vs* CTRL (middle), and DUP *vs* DEL (right) for 3M organoids. Proteins within 16p11.2 CNV are colored in pink. Proteins colored in orange are upregulated; proteins colored in blue are downregulated. **(B)** GO terms enrichment analyses for differentially expressed proteins in 3M organoids for DEL *vs* CTRL (left), DUP *vs* CTRL (middle), and DUP *vs* DEL (right).

**Figure S16. Scale-free topology of protein co-expression networks.** The soft threshold power of 13 for 1M organoids and 17 for 3M organoids was chosen to correspond to the scale-free topology fit index of 0.8 or higher.

**Figure S17.** **Protein co-expression modules for 1M and 3M organoids.** WPCNA cluster dendrogram and module eigengene-genotype association are shown. The data from all samples (DEL, DUP and CTRL) were used for module detection. Comparisons were made between each genotype (DEL or DUP) and control (CTRL) for each module enrichment to calculate the significance. Rows are genotypes (relative to CTRL) and columns are modules. Number in each tile is a beta value from linear mixed effect model, and color of each tile indicates statistical significance (see **Materials and Methods**). A total of 5 modules were significantly associated with DEL or DUP genotypes in 1M old organoids, and a total of 7 modules in 3M old organoids.

**Figure S18. Protein co-expression modules enrichment analyses.** Hierarchical clustering of protein co-expression modules by module eigengene is shown. Significant module-genotype associations (*) at FDR<0.1 are shown below each module. Significant module enrichment analyses (*) at FDR<0.05 against literature-curated gene lists with previous evidence for involvement in autism are shown at the bottom. The lists include syndromic and highly ranked (1 and 2) genes from SFARI Gene database (<https://gene.sfari.org/database/gene-scoring/>); pre- and post-synaptic genes from SynaptomeDB {Pirooznia, 2012 #6933}; genes with probability of loss-of-function intolerance (pLI)>0.99 as reported by the Exome Aggregation Consortium {Karczewski, 2020 #7030}; constrained genes {Samocha, 2014 #6369}; FMRP target genes {Darnell, 2011 #5916}, and CHD8 target genes {Wilkinson, 2015 #6932}. All detected modules are shown.

**Figure S19. Correlation of the entire transcriptome *vs* proteome in 3M organoids**. Correlation of entire transcriptomes *vs* proteomes in DEL *vs* CTRL (left), DUP *vs* CTRL (middle), and DUP vs DEL (right) comparisons for 3M organoids. Genes/proteins within 16p11.2 CNV *locus* are colored in pink.

**Figure S20. Correlation of significant differentially expressed genes or proteins in 1M organoids.** The correlation was calculated for a combined set of the DEGs and DEPs, with or without 16p11.2 genes. Genes/proteins within 16p11.2 CNV *locus* are colored in pink.

**Figure S21. Module preservation analyses for gene co-expression, protein co-expression and migration, neuronal/synaptic and 16p11.2 modules.** Module preservation scores of gene co-expression modules (*vs*. corresponding protein co-expression modules) for 1M old organoids (left) and 3M old organoids (right) are shown. Gene modules significantly associated with genotypes (DEL and DUP) are marked. Z-scores above 2 are considered to be conserved, and above 10 are highly conserved. Module preservation scores of 16p11.2 (*16lightcyan*), neuronal/synaptic (*25orange*) and migration (*32violet*) gene co-expression modules (vs. iPSC and 1M transcriptomic, and 1M and 3M proteomic modules) for 3M old organoids are shown (bottom). Z-scores above 2 are considered to be conserved.

**Figure S22. Flow cytometry of cerebral organoids.** Representative images of histograms used for the flow cytometry analysis of dissociated cerebral organoids. Events with higher fluorescence than background histograms (within the area delimited by bars) were considered positive and quantified.

**Figure S23. Proliferation rate in 1M old organoids.** Upper panel: **r**epresentative images of 1M organoid slices immunostained with DAPI, Ki67 and Edu. Scale bar: 100µm. Bottom panel: quantification of the percentage of positive cells for each marker in each genotype and cell cycle exit ratio. Symbols represent organoids from the same differentiation batch, where batch is defined as CTRL, DEL, DUP from one patient, one clone and one replica. Data is presented as mean ± SEM (*n=*2 patients per genotype, at least 4 organoids per patient). Significance was calculated using one-way ANOVA with Tukey’s multiple comparison; ****p*<0.001, ***p*<0.01, **p*<0.05. Significance above bars represents comparison against CTRL.

**Figure S24**. **Wnt signaling genes downregulated in 16p11.2 DEL organoids**. Heat plot represents TPM values of gene expression for Wnt signaling genes from *22darkgreen* module.

**Figure S25. Statistical analyses of migration in DEL and DUP organoids by distance.** The bins for 0-200 µm and 200-400 µm distances are shown. Significance was calculated using one-way ANOVA with Tukey’s multiple comparison; **p*<0.05.

**Figure S26. Impaired migration in DEL and DUP organoids.** Upper panel: **r**epresentative images of neurons migrating out of Matrigel-attached organoids at the indicated time points after the start of time-lapse. Arrows mark individual neurons. Bottom panel: quantification of total distance traveled by individual neurons (mean ± SEM; one-way ANOVA with Tukey’s multiple comparison, ***p<0.001, *p<0.05; n=4 neurons per organoid, 3-4 organoids per genotype). Tracing of cell movement of individual representative neurons for each genotype is shown on the right. Each dot represents location of the neuron after 1h time period.

**Figure S27.** **Immunostaining of 1M old organoids with neuronal, developmental and intermediate filament markers.** Representative images of 1M old organoid attached in Matrigel, stained with DAPI, Sox2 and NeuN (left), DAPI and Nestin (right). Scale bar: 100µm.

**Figure S28. Western Blots images of total and active RhoA.** Western Blot images of 1M organoids for KCTD13, actin as loading control, total RhoA, and active RhoA (RhoA-GTP). Organoids were grown in the batches of CTRL, DEL and DUP for each experiment. Six batches were grown for each experiment.

**Figure S29. Statistical analyses of migration rescue by Rhosin in DEL and DUP organoids by distance.** The bins for 0-200 µm and 200-400 µm distances are shown. Significance was calculated using two-way ANOVA with Tukey’s multiple comparison; ****p*<0.001, **p*<0.05.

**Figure S30. Migration rescue from intact organoids and from Boyden chamber experiments with Rhosin plotted by batch.** Graphs representing the quantification of the migration experiments plotted by batch. (Top panels) Quantification of cells migrating from the organoid plotted by batch. Batch #1 left column, Batch #2 right column. The number of cells migrating for shorter distances (0-200 µm) is increased and for longer distances (200-400 µm) is decreased in DEL_Vh and DUP_Vh from both batches. After Rhosin treatment, the number of cells migrating for shorter distances (0-200 µm) decreases and for longer distances (200-400 µm) increases in DEL_Rh and DUP_Rh from both batches, and becomes indistinguishable from CTRLs. (Bottom panels) Boyden Chamber experiments from the dissociated organoids are shown. Treatment with Rhosin increases the number of migrating cells in both, DELs and DUPs. Significance was calculated using two-way ANOVA with Tukey’s multiple comparison; ***p*<0.01, **p*<0.05.

**Figure S31. Rhosin does not rescue neurite length phenotype.** Representative images of neurons from dissociated organoids 8 days after dissociation, immunostained with DAPI (blue) and MAP2 (green). Quantification of total neurite length for each genotype with or without Rhosin treatment is shown. Symbols represent organoids from the same differentiation batch, where batch is defined as CTRL, DEL, DUP from one patient, one clone and one replica. Data is presented as mean ± SEM (n=2 patients per genotype, at least 15 neurons per patient). Significance is calculated using two-way ANOVA with Tukey’s multiple comparison; ***p<0.001, **p<0.01, *p<0.05. Significance above bars represents comparison against CTRL.

**Supplementary Table legends**

**Supplementary Table 1.** 16p11.2 patient-derived fibroblast selection and patients’ clinical information.

**Supplementary Table 2.** RNA-seq parameters and quality control metrics from Cutadapt, STAR, Picard, and RNA-SeQC for iPSCs, 1M and 3M old organoids representing 108 transcriptomes.

**Supplementary Table 3.** Statistical analyses of cortical organoids size measurements.

**Supplementary Table 4.** Differentially Expressed Genes (DEGs) in iPSCs, 1M and 3M cortical organoids.

**Supplementary Table 5.** Gene Ontology enrichment analysis of differentially expressed genes.

**Supplementary Table 6.** Module membership from gene co-expression (WGCNA) analysis in iPSCs, 1M and 3M cortical organoids.

**Supplementary Table 7.** Gene Ontology enrichment analyses of significantly genotype-associated gene co-expression modules from WGCNA.

**Supplementary Table 8.** Differentially Expressed Proteins (DEPs) in 1M and 3M cortical organoids detected by LC-MS/MS with TMT labeling.

**Supplementary Table 9.** Gene Ontology enrichment analysis of differentially expressed proteins.

**Supplementary Table 10.** Module membership from protein co-expression (WPCNA) analysis in 1M and 3M cortical organoids.

**Supplementary Table 11.** Gene Ontology enrichment analyses of significantly genotype-associated protein co-expression modules from WPCNA.

**Supplementary Table 12**. Summary of experiments by patients, clones and replicates.

**Supplementary Table 13**. Raw data for all experimental Figures.
